# The Influence of Malnutrition and Micronutrient Status on Anemic Risk in Children under 3 Years Old in Poor Areas in China

**DOI:** 10.1371/journal.pone.0140840

**Published:** 2015-10-21

**Authors:** Jie Wang, Hui Wang, Suying Chang, Liyun Zhao, Ping Fu, Wentao Yu, Qingqing Man, Robert Scherpbier, Lili Pan, Yifan Duan, Shi-an Yin

**Affiliations:** 1 Department of Maternal and Child Nutrition, National Institute for Nutrition and Health, Chinese Center for Disease Control and Prevention, Beijing, China; 2 Department of Population Research, China Population and Development Research Center, Beijing, China; 3 Section of Health and Nutrition and Water, Environment and Sanitation, United Nations Children’s Fund, Beijing, China; 4 Department of Nutrition Surveillance, National Institute for Nutrition and Health, Chinese Center for Disease Control and Prevention, Beijing, China; 5 Department of Nutrition on Aging, National Institute for Nutrition and Health, Chinese Center for Disease Control and Prevention, Beijing, China; Oklahoma State University, UNITED STATES

## Abstract

**Background:**

Malnutrition and anemia affect large numbers of young children living in poor areas of China. Multi-micronutrient deficiencies may be related to the prevalence of anemia in different populations, and identifying the risk factors that render children susceptible to anemia is the first step in combating anemia effectively.

**Methods:**

In this cross-sectional study, a total of 1370 children under 3 years old were selected based on probability proportional to size sampling principles from poor counties of China. Basic characteristics data were collected by questionnaire; then anthropometrics and hemoglobin were measured in the field and anemia prevalence evaluated. Venous blood was drawn from children aged 12–35 months (N = 553) to evaluate micronutrient status. Logistic regression was used to identify the risk factors for children’s anemia.

**Results:**

Among children aged 0–35 months, the prevalence of stunting, low body weight and wasting was 17.5%, 8.6% and 5.1%, respectively, and 25.6% of the children were affected by anemia, with more anemic infants and younger children than older children (*P* <0.01). There were 26.5%, 12.8%, 14.1% and 20.0% of the children aged 12–35 months affected by iron deficiency, vitamin D deficiency, folic acid deficiency and vitamin B_12_ deficiency, respectively. For children aged 0–11 months who were breastfed, the mothers’ anemic status was the only factor associated with the child’s anemia (OR = 2.6; 95% CI: 1.2–5.4, *P* < 0.05). For children aged 12–35 months, multivariate logistic regression indicated that anemia was significantly associated with iron and vitamin B_12_ deficiency (OR = 5.3; 95% CI: 1.9–14.5, *P* < 0.01) and monotonous diet (OR = 2.3; 95% CI: 1.1–4.7, *P* < 0.05) after adjusting for age and gender.

**Conclusion:**

The prevalence of anemia was higher in children under 2 years old and requires urgent intervention. An effective intervention strategy should include iron and vitamin B_12_ supplements_,_ improving dietary diversity and controlling breastfeeding mothers' anemia.

## Introduction

Anemia and iron deficiency are the most common disorders in young children. Worldwide, the estimated prevalence of anemia is 47.4% in preschool-aged children, and iron deficiency is likely higher than anemia [1]. In China, it was reported that 22% of children under 5 years old were anemic in poor areas, and approximately 40% of infants and 18%-32% of young children were affected by anemia. Furthermore, there was basically no change in the prevalence of anemia in children under 5 years old between 2005 and 2009 [2]. Anemia remains a public health problem for children, particularly in the poor areas of China. Identifying the risk factors of anemia susceptibility in children is the first step in developing an effective and feasible strategy to combat anemia and decrease the harm anemia causes to human health.

Anemia is harmful to people at all stages of life, but particularly for infants and young children. Anemia has been conclusively determined to delay psychomotor development and impair the cognitive performance of infants; children who were anemic during infancy scored 5–10 points lower on intelligence tests and other cognitive performance indicators upon entering school than children who were not anemic during infancy. The effects of iron deficiency anemia in infancy and early childhood are likely not correctable by subsequent iron therapy. Morbidity from infectious diseases increases in iron-deficient populations, and iron supplementation among deficient children reduces morbidity because of a reduced capacity of leukocytes to kill ingested microorganisms and a decreased ability to replicate lymphocytes when stimulated by a mitogen [3].

Excessive blood loss, increased blood cell destruction and deficient red blood cell production are the three primary causes of anemia. Of these reasons, deficiencies such as in iron, vitamin B_12_ and folic acid for hemoglobin and DNA synthesis during red blood cell production are the primary reason for anemia [4]. These deficiencies may lead to two types of anemia, megaloblastic anemia because of vitamin B_12_ or folic acid deficiency and iron deficiency anemia resulting from deficient heme synthesis. A study in Turkish adolescents concluded that 59% of anemic children were iron deficient, 41% of those children were iron and vitamin B_12_ deficient, and none was deficient in folic acid [5]. In elderly individuals in the Netherlands, anemia was associated with folate deficiency and elevated homocysteine levels but not associated with vitamin B_12_ deficiency [6].

Deficits in other micronutrients may increase the risk of anemia. One randomized clinical trial in children to compare the treatment effects of iron supplement (IS), iron plus folic acid supplement (IFS) and multiple micronutrient supplement (MMS) concluded that the prevalence of anemia decreased more significantly in the MMS (14%) and IFS (11%) groups than in the IS group [7], which suggests that other micronutrient deficiencies may contribute to the prevalence of anemia. The association between anemia and vitamin A or vitamin D deficiency was not consistent [8, 9]. Improvement in vitamin A status has generally been shown to reduce the prevalence of anemia; however, the actual public health effect on anemia has yet to be demonstrated [10].

Furthermore, previous studies have identified other risk factors for anemia in children under 5 years such as a young age [11–13], retarded growth [11–13], disease status [11–14], diet [10, 15] and mother’s low hemoglobin level [2, 11, 16].

However, limited evidence was available to evaluate the association between anemia and micronutrient status in infants and young children, the most vulnerable population for anemia. Multi-micronutrients were usually administered to achieve an effective intervention, which, although affordable, is difficult to extend to a large population over time. This study was conducted to understand the risk factors associated with anemia, including age, gender, growth, micronutrient status, breastfeeding, diet, disease, parental education and maternal anemia, in children under 3 years old in remote and poor rural areas of China. The goals of the study were to provide scientific evidence and cost-effective approaches to combating anemia in the target population.

## Subjects and Methods

### Participants

The cross-sectional survey was conducted in 3 national poverty counties in China that were selected by the Millennium Development Goals project. Forty-one villages were selected from among all of the villages in the 3 counties by probability proportional to size (PPS, [17]) sampling methods based on 2010 population estimates. Cluster sampling was utilized for household selection, and 1336 households having at least one child aged 0–35 months were involved in this study. There were 1370 children and 34 sibling pairs participating in the study, and all of the questionnaires, anthropometry and blood samples were collected between August 21 and September 9 of 2010.

This study was approved by the Ethical Review Committee of the National Institute for Nutrition and Health at the Chinese Center for Disease Control and Prevention. Written informed consent was obtained from all children’s parents or caregivers.

### Questionnaire

The field survey was conducted by health workers in county medical care centers. Health workers were well trained and supervised by qualified and experienced experts before and during data collection. A questionnaire was used to collect data on children’s birth information (from birth certificates), diarrhea and respiratory disease during the previous two weeks, 24-hour dietary intake (WHO IYCF) [18] and parental education and occupational experience. Children’s diets were defined as diverse if the children consumed foods from 4 of 7 food categories (grains, roots and tubers; legumes and nuts; dairy products [milk, yogurt, cheese]; flesh foods [meat, fish, poultry and liver/organ meats]; eggs; vitamin A rich fruits and vegetables; other fruits and vegetables); otherwise their diets were designated monotonous. The questions were answered by the children’s mothers or caregivers.

### Anthropometry

Body weight was measured and recorded to the nearest 0.01 kg with platform weighing scales (TC100KA, 0–100 kg of capacity and 10 g of accuracy, Huatec Company, China). Children wore only underwear. The length of children less than 24 months of age and the height of children older than 24 months were measured to the nearest 0.1 cm using a body length scale (YSC-2, Beijing Guowangxingda Weight Scale Company, China) and a body height scale (TZG, Jiangyin Medical Equipment Company, China). The scales were calibrated before each examination.

### Blood samples for hemoglobin and micronutrients tests

Three to five milliliters of blood were drawn from the children aged 12–35 months (N = 553) and breastfeeding mothers (N = 382), and one drop of venous blood was used to measure hemoglobin at the field site. Blood samples were covered by aluminum foil to protect them from light. Serum was separated and stored at -20°C at county hospitals, then shipped on ice within one month of fieldwork to Beijing and stored at -70°C until analysis.

For children younger than one year old or for participants who did not voluntarily give venous blood, finger blood was used to measure hemoglobin level by HemoCueHb 301 (Angelholm, Sweden). Hemoglobin results were similar in finger and venous blood samples [19]. Anemia was defined as hemoglobin < 11 g/dL for children aged 6–35 months and < 12 g/dL for the mothers, and all the cutoffs were adjusted for altitude [3]. Because there was no hemoglobin cut-off for children younger than 6 months, we used the same cut-off as for children aged 6–35 months to assess anemia.

Serum retinol was measured by HPLC (Waters 600E) [20]. Serum 25-OH-vitamin D was analyzed with the DiaSorin 25-OH-vitamin D ^125^I RIA KIT (Stillwater, Minnesota 55082–0285, U.S.A) and XH6080 Radioimmunoassay (Xi’an Nuclear Instrument Factory, China). Serum ferritin was measured by the ^125^I Ferritin Radioimmunoassay Kit (Beijing North Institute of Biological Technology, China) and XH6080 Radioimmunoassay. Folic and vitamin B_12_ were measured by the Simui TRAC-SNB Radioassay Kit (MP Biomedicals LLC, Germany) and XH6080 Radioimmunoassay. C reactive protein was measured by Nanopia CRP (Sekisui Medical Co., Ltd., Japan) using Toshiba 120 Automatic Biochemistry Analyzers (Toshiba, Japan).

Iron deficiency was defined as ferritin levels of less than 12 μg/L [3], and the analysis was restricted to children with C-reactive protein of less than 5 mg/L [21]. Cutoff values were 0.7 μmol/L for vitamin A deficiency and 1.05 μmol/L for vitamin A insufficiency [22], 50 nmol/L for vitamin D deficiency [23], 4 ng/mL for folic acid deficiency [24], and 203 pg/mL for vitamin B_12_ deficiency [25].

### Statistical analysis

Statistical analysis was conducted using SAS software (version 9.1; SAS Institute, Inc., Cary, North Carolina). Data were expressed as the mean ± SD or median (range) for continuous variables and as frequencies for categorical variables. WHO Anthro software was used to calculate the children’s growth status (HAZ/LAZ, height/length for age Z score; WAZ, weight for age Z score; WHZ/WLZ, weight for height/length Z score; BAZ, BMI for age Z score). Stunting (HAZ/LAZ < -2), low weight (WAZ < -2), wasting (WHZ/WLZ < -2) and overweight (BAZ > +2) were estimated [26]. We used the general linear models and Chi-square test to compare the means of variances and the differences in contingency tables. Univariate logistic regression was used to analyze the potential risk factors for anemia; the risk factors (*P* < 0.2) were then fed into stepwise multivariate logistic regression models with sls = 0.15 and sle = 0.1 to assess the odds ratios (OR) and 95% confidence intervals (CI). *P* value < 0.05 is considered to be statistically significantly different.

## Results

### Characteristics and malnutrition prevalence

A total of 1370 children with a median age of 15.6 (35.5) months were enrolled in the study, and 51.9% were boys. At birth, the mean weight of the children was 3.2 ± 0.4 kg, and boys were 0.1 kg heavier than girls (*P* < 0.05). At measuring time, the boys were taller and heavier than the girls, as shown in Table 1. However, according to the WHO child growth standard, more boys were affected by stunting than girls (20.5% and 14.3%, *P* < 0.05). The overall prevalence of stunting, low body weight, wasting and overweight were 17.5%, 8.6%, 5.1% and 2.5%, respectively. The stunting prevalence was lowest in children under 6 months and highest in children aged 18–23 months (6.4% and 24.3%, *P* < 0.05). The prevalence of low weight was lowest in children under 6 months and highest in children aged 12–17 months (3.4% and 12.9%, *P* < 0.05).

**Table 1:** Anthropometry and Z scores of children aged 0–35 months^1^.

Anthropometry/Z scores	Boys	Girls	Total
Height/length (cm)	76.81 ± 9.50 *	75.42 ± 9.68	76.14 ± 9.61
Weight (kg)	9.71 ± 2.20 *	9.13 ± 2.23	9.43 ± 2.23
Height/length for age z score	-0.89 ± 1.34 *	-0.65 ± 1.24	-0.77 ± 1.30
Weight for age z score	-0.62 ± 1.12 *	-0.50 ± 1.04	-0.57 ± 1.08
Weight for height/length z score	-0.21 ± 1.15	-0.21 ± 1.06	-0.21 ± 1.11
BMI for age z score	-0.12 ± 1.13	-0.15 ± 1.06	-0.14 ± 1.10

^1^ The results were expressed as the mean ± SD

*P < 0.05 for comparisons between boys and girls

### Micronutrient status

The mean concentrations of serum ferritin, retinol, vitamin D, folic acid and vitamin B_12_ in children aged 12–35 months are shown in Table 2. The concentrations of ferritin and vitamin B_12_ were slightly higher in girls than in boys; however, the difference was not significant (*P* > 0.05). The proportions of the children with iron deficiency, vitamin A deficiency, vitamin A insufficiency, vitamin D deficiency, folic acid deficiency and vitamin B_12_ deficiency accounted for 26.9%, 0.0%, 54.1%, 12.8%, 14.1% and 20.0% of the children aged 12–35 months, respectively. Of the children, 1.8% showed abnormal C-reactive protein.

**Table 2:** Micronutrient status in children aged 12–35 months ^1^.

Micronutrients	N	Boys	Girls	Total
Hemoglobin (g/L)	1370	117.5±12.7*	119.4±12.5	118.4±12.7
Vitamin A (μmol/L)	418	1.0±0.2	1.1±0.2	1.0±0.2
Vitamin D (nmol/L)	538	68.0±20.5	68.3±21.0	68.1±20.7
Ferritin (μg/L) ^2^	513	20.5±12.4	22.6±18.7	21.5±15.7
Folic acid (ng/mL)	469	10.7±9.4	10.8±10.5	10.8±10.0
Vitamin B_12_ (pg/mL)	446	332.5±158.3	361.9±170.4	346.9±164.8

^1^ The results were expressed as the mean ± SD

^2^ for children with C-reactive protein less than 5 mg/L

*P < 0.05 for comparisons between boys and girls

### Prevalence of anemia

The mean hemoglobin concentration of the children was 118.4 ± 12.7 g/L, and the prevalence of anemia was 25.6%, with more boys facing the risk of anemia than girls (*P* < 0.05). The prevalence of anemia decreased by age, as shown in Table 3. The prevalence of anemia was 41.4%, 31.6% and 7.6% in children younger than 1 year, younger than 2 years and older than 24 months, respectively. Anemia attacked 12.1% of the mothers who were breastfeeding their infants. The proportions of the children with anemia were 66.7% and 43.5% in the anemic mothers group and non-anemic mothers group (*P* < 0.05).

**Table 3:** Prevalence of anemia in children by age group (%).

Age (months)	Boys	Girls	Total
0–5	54.9*	37.2	46.1
6–11	39.4	37.2	38.3
12–17	27.5	24.4	26.1
18–23	20.2	13.7	16.9
24–29	10.8	7.9	9.3
30–35	9.1	1.4	5.6
Total	28.4*	22.5	25.6

*P < 0.05 for comparisons between boys and girls

Siblings (N = 68, 34 pairs) composed 5% of the children. The prevalence of anemia was 25.0% in sibling pairs and 25.6% in the other children. Among the siblings, 38.2% (N = 26) were twins, and the prevalence of anemia was 21.4% for the boys and 16.7% for the girls (*P* = 0.76). For non-twin births in the same families, the younger children were at greater risk of anemia (47.6%) than the older children (9.5%) (*P* < 0.05).

### Micronutrient status in anemic and non-anemic children

Because of the greater prevalence of anemia in children aged 12–23 months than in children 24–35 months, we divided all children aged 12–35 months with micronutrient status into four groups, as shown in Table 4. We compared the micronutrient status of these four groups and observed that only hemoglobin levels were significantly different between non-anemic and anemic children in the same age group (*P* < 0.05, Table 4). Moreover, mean vitamin B_12_ concentration was higher in non-anemic children than in anemic children in the same age group (*P* > 0.05). For anemic children, the vitamin D level was higher; however, the ferritin level was lower for children aged 12–23 months than for children aged 24–35 months (*P* < 0.05).

**Table 4:** Micronutrient status in anemic and non-anemic children by different age groups^1^.

Indicators	Non-anemia				Anemia			
	N	12–23 months	N	24–35 months	N	12–23 months	N	24–35 months
Hemoglobin (g/L)	238	125.5 ± 8.0 b	261	127.1 ± 7.6 a	41	107.8 ± 6.6 c	13	103.9 ± 9.8 c
Vitamin A (μmol/L)	186	1.0 ± 0.2	189	1.0 ± 0.2	33	1.0 ± 0.1	10	1.0 ± 0.1
Vitamin D (nmol/L)	229	71.3 ± 18.6 a	256	64.9±22.6 ab	40	74.4±16.8 a	13	57.0±13.7 b
Ferritin (μg/L)	216	19.0 ± 13.4 bc	246	24.2±17.0 ab	38	15.4±14.4 c	13	28.0±18.1 a
Folic (ng/mL)	194	10.8 ± 8.5	225	10.7±10.9	37	12.5±12.5	13	8.0±4.5
Vitamin B_12_(pg/mL)	184	364.7 ± 177.6	213	342.5±154.3	36	295.0±154.7	13	309.1±145.9

^1^ The results were expressed as the mean ± SD, and values in the same row without the same letter were different, *P* < 0.05.

### Risk factors of anemia

In univariate logistic regression analyses, anemia was significantly associated with younger age, male gender, iron deficiency, vitamin B12 deficiency, monotonous diet, and diarrhea in the previous two weeks and breastfeeding mothers’ anemia (*P* < 0.05). Anemia was associated with vitamin A insufficiency and migrant mothers who migrated to cities for work and left children at home (Table 5). We did not observe an association between anemia and folic acid deficiency, vitamin D deficiency, low birth weight, stunting, low body weight, wasting, whether children were ever breastfed and early initiation of breastfeeding.

**Table 5:** Risk factors of anemia by univariate logistic regression.

Risk factors	N	OR (95% CI)	*P*
Age < 24 months	1370	5.6 (3.7, 8.6)	< 0.01
Boys	1370	1.4 (1.1, 1.8)	0.01
Low birth weight	1271	1.2 (0.6, 2.2)	0.58
Stunting	1370	1.0 (0.8, 1.4)	0.78
Underweight	1370	0.9 (0.5, 1.3)	0.49
Wasting	1370	1.3 (0.7, 2.1)	0.38
Iron deficiency	513	2.3 (1.3, 4.1)	< 0.01
Vitamin B_12_ deficiency	446	3.0 (1.6, 5.6)	< 0.01
Folic acid deficiency	469	1.0 (0.4, 2.3)	0.99
Vitamin A insufficiency	418	1.7 (0.9, 3.2)	0.13
Vitamin D deficiency	538	1.0 (0.4, 2.4)	0.93
Monotonous diet (over 6 months old)	1062	1.6 (1.2, 2.2)	< 0.01
Ever breastfed	1292	1.3 (0.8, 2.1)	0.25
Early initiation of breastfeeding	1125	1.0 (0.7, 1.5)	0.81
Respiratory disease	1291	0.9 (0.7, 1.3)	0.60
Diarrhea	1290	1.4 (1.0, 2.0)	0.04
Migrant mothers	1290	0.6 (0.4, 1.1)	0.08
Maternal anemia	365	2.4 (1.3, 4.6)	< 0.01
Mother’s education (years)			
0	53	1.7 (0.9, 3.2)	0.08
1–6	348	1.0 (0.7, 1.3)	0.91
7–9	677	1.1 (0.7, 1.8)	0.56
10–12	141	1.1 (0.6, 2.0)	0.73
12+	72	1.0	-
Father’s education (years)			
0	11	0.6 (0.1, 3.1)	0.57
1–6	240	0.7 (0.4, 1.2)	0.17
7–9	736	1.0 (0.6, 1.6)	0.98
10–12	194	1.1 (0.6, 1.8)	0.75
12+	111	1.0	-

The factors associated with anemia derived from univariate logistic regression (*P* < 0.2) were included in the multivariate logistic model (Table 6). For infants (Model 1), maternal anemia was the only risk factor associated with their breastfed children’s anemia; in children aged 12–35 months (Model 2), anemia was significantly associated with vitamin B_12_ deficiency and monotonous diet after adjusting for age, gender and diarrhea. Children aged 12–35 months were categorized into sub-groups to analyze the interaction of iron deficiency and vitamin B_12_ deficiency (Model 3), and the results showed that the monotonous diet and deficiency of both iron and vitamin B_12_ were associated with anemia after adjusting for age and gender. Risk factors for anemia were consistent in the regression analyses with or without the 34 sibling pairs.

**Table 6:** Risk factors of anemia by stepwise multivariate logistics.

Risk factors	OR (95% CI)	*P*
Model 1: For currently breastfed children aged 0–11 months		
Maternal anemia	2.6 (1.2, 5.4)	0.01
Model 2: For children aged 12–35 months		
Younger children (12–23 months)	2.9 (1.3, 6.3)	< 0.01
Boys	2.1 (1.0, 4.4)	< 0.05
Vitamin B_12_ deficiency	2.5 (1.2, 5.3)	0.02
Monotonous diet	2.1 (1.1, 4.4)	0.04
Diarrhea	2.6 (0.9, 7.3)	0.08
Model 3: For children aged 12–35 months with interaction		
Younger children (12–23 months)	2.5 (1.1, 5.6)	0.02
Boys	2.3 (1.1, 4.7)	0.03
Monotonous diet	2.3 (1.1, 4.7)	0.02
Interaction of ferritin and vitamin B_12_		
Ferritin deficiency	1.0 (0.4, 2.5)	0.99
Vitamin B_12_ deficiency	1.1 (0.4, 3.3)	0.85
Iron and B_12_ deficiency	5.3 (1.9, 14.5)	< 0.01
Without iron and B_12_ deficiency	1	-

## Discussion

### High prevalence of malnutrition

The present study provided a systematic analysis of the risk factors of susceptibility to anemia in children in poor and remote areas of China, including the child’s gender, age, rate of growth, disease, micronutrient status, breastfeeding, diet, parents’ education and mother’s anemia. The children’s average birth weight (3206 g) in the present study was lower than the national mean level of 3372 g. The prevalence of stunting and underweight (17.5% and 8.6%) was higher than the rate of the national rural level (12.6% and 4.6%) [2]. Boys were more at risk for stunting in the present study, consistent with previous studies [27–30].

### Children aged 0–24 months with greater susceptibility to anemia

The prevalence of anemia in children under 3 years was 25.6%, which was slightly higher than the rate of 22.0% in children under 5 years old in poor Chinese rural areas [2]. Both the present study and several previous reports have observed that boys and younger children are more susceptible to anemia [31–34]. The prevalence of anemia in children aged 0–11 months (41.4%) in this study was similar to the data in poor rural areas in 2009 (41.8%); however, the prevalence of anemia in children aged 12–23 months and 24–35 months (21.8% and 7.6%) was less than the surveyed results in 2009 (32.1% and 18.3%) [2]. These data indicate that anemia in children requires intervention in poor rural areas in China. When combating anemia, the key population is children under 2 years old. China implemented a Child Nutrition Improvement Strategy of Micronutrient Supplementation for children aged 6–24 months in one-sixth of the poor counties in 2012. This strategy must be expanded to the extensive poor areas to benefit more susceptible children. Furthermore, the strategy should include how to improve the anemia status of children younger than 6 months who are breastfed.

### Anemia primarily associated with deficiency of iron and vitamin B_12_ but not with other micronutrients

Iron deficiency and vitamin B_12_ deficiency were the primary micronutrient problems. Comparing the prevalence of anemia, we observed that more children with iron deficiency were affected by anemia than children with normal iron levels (15.9% and 7.7%, *P* < 0.01), and more children with vitamin B_12_ deficiency were anemic than children with normal vitamin B_12_ levels (21.4% and 8.4%, *P* < 0.01). By the multivariate logistic regression model, we identified the primary risk factor for anemia as deficiencies of iron and vitamin B_12_ after adjustment for age, gender and monotonous diet in children aged 12–35 months. These findings could indicate the improved intervention results of multiple micronutrient supplements compared with an intervention of only iron supplementation [7].

The present study concluded that folic acid was not a risk factor for children’s anemia, which was consistent with the results that indicated no benefit from a folic acid supplement to prevent anemia in pregnant [35] and postpartum women [36]. For the anemic populations with little or no folic acid deficiency, fortification or supplementation of folic acid could not help fight anemia.

A previous study observed that vitamin A insufficiency (serum retinol < 1.05 μmol/L) was a risk factor for anemia (OR 1.83, 95% CI: 1.10–3.05, *P* < 0.05) in children aged 12–72 months in Vietnam [37], which is inconsistent with the present study. When comparing the retinol results in these two studies, we observed that the mean retinol concentration was the same as 1.0 μmol/L; however, 10% of the children were retinol deficient (serum retinol < 0.7μmol/L) in the previous study, and no child was retinol deficient in the present study. The different retinol levels in different groups of children may explain the different findings.

The present study observed that there was no association between vitamin D and hemoglobin levels in children aged 12–35 months (mean age, 22.5 months), which is consistent with one previous study on children (mean age, 21.7 months) [38]. Some studies in adults (mean age, 65 years) [39], in HIV-infected women [40] and pre- and post-menopausal women [41] reported that vitamin D deficiency was a risk factor for anemia. The effect of vitamin D on anemia in different populations must be studied further.

### Anemic mothers’ infants at greater risk for anemia

Breast milk is the primary nutrient source for infants, and the high prevalence of anemia in infants may be ascribed to the poor nutrition of their mothers. The present study reveals that currently breastfed infants whose mothers were affected by anemia were at greater risk for anemia than breastfed infants whose mothers were not anemic. Similar findings were reported in previous studies indicating that the mother’s anemia was a risk factor for child anemia in children aged 6–59 months in Haiti (OR = 1.8; 95% CI: 1.2–2.9; *P* = 0.011) [11] and in children aged 6–36 months in Burma (OR = 11.8; 95% CI: 7.6–18.5; *P* < 0.001) [12]. The correlation between a lactating mother’s vitamin D status and her child's status has been identified [42, 43]. Therefore, anemia prevention and intervention for infants and young children should target both breastfeeding mothers and their babies to achieve an optimum effect.

### Diverse diet as a protective factor against anemia in young children

It has been reported that food insecurity is associated with anemia. Among adolescents aged 12–15 years, the odds of iron deficiency anemia were 2.95 times (95% CI: 1.18, 7.37) higher for children in households with food insecurity than for children in households with food security [44]. Among pregnant women, those who lived less than 0.25 miles from a healthy food source were less likely to be anemic than pregnant women who lived farther (adjusted OR = 0.65, 95% CI: 0.48, 0.88) [45]. In adult Mexican women, the prevalence of anemia was 31%-43% higher among those living in mild to severe food-insecure households than among adult women residing in food-secure households (P < 0.05) [46]. The present study observed that a monotonous diet is associated with anemia in children over 1 year (OR = 2.3, 95% CI: 1.1, 4.7, P < 0.05). Based on the above evidence, nutrition advocacy for food choice for both the mother and the child over 1-year-old may be an effective and relatively easy manner in which to improve micronutrient deficiency and fight anemia over the long term.

### Strategies for combating anemia

This cross-sectional study identified the risk factors for anemia in children in poor areas in China. Our findings have important programmatic implications. First, the most vulnerable population for anemia is children under 24 months. Second, multiple micronutrient supplements, including iron and vitamin B_12,_, may help combat anemia. Third, preventing anemia in breastfeeding mothers would promote the prevention of anemia in children. Fourth, good-quality diets for children will guarantee children’s good nutrition and good health.

### Limitations

This study was a cross-sectional study conducted in 3 similar poor, remote, mountainous, and difficult-to-reach counties to identify situational and potential risk factors of anemia in infants and young children. However, the correlation between children’s micronutrients and anemia was observed in children older than 1 year, although not all families consented to blood collection. Therefore, the association of micronutrients with anemia for infants requires further study to be substantiated.

### Conclusions

The present findings indicate that children under 2 years old are at high risk of anemia in these poor areas and that interventions are urgently required. The risk factors analysis indicates that breastfed infants whose mothers were anemic and young children with iron and vitamin B_12_ deficiency and lack of diversity in their diets are at greater risk for anemia. Therefore, an effective intervention strategy in this population should include iron and vitamin B_12_ supplements, improvements in dietary diversity, and controlling breastfeeding mothers' anemia.

## Supporting Information

S1 File Malnutrition, micronutrients and anemia in children. (DOCX)
