# Supplementary material for: The Influence of Malnutrition and Micronutrient Status on Anemic Risk in Children under 3 Years Old in Poor Areas in China
Source: PLoS One. 2015 Oct 21;10(10):e0140840. doi: 10.1371/journal.pone.0140840 (PMC4619061; doi:10.1371/journal.pone.0140840)
Supplement: S1 File — (DOCX) [file pone.0140840.s001.docx]

| seq | age | gender | bw | bl | edu_father | edu_mom | respi | diarr | length | wt | hb | B12 | folic | ferr | vd2 | va | food |
| --- | --- | --- | --- | --- | --- | --- | --- | --- | --- | --- | --- | --- | --- | --- | --- | --- | --- |
| 1 | 20.96 | 2 | 3750 |  | 3 | 3 | 1 | 1 | 81.5 | 9.51 | 12 |  |  |  |  |  | 6 |
| 2 | 29.83 | 1 | 3000 | 48 | 3 | 2 | 2 | 1 | 85.5 | 10.1 | 11.9 | 291.15 | 11.77 | 33.68 | 41.71 |  | 2 |
| 3 | 24.74 | 2 | 3900 |  | 4 | 4 | 1 | 1 | 84 | 11.26 | 10 |  |  |  |  |  | 5 |
| 4 | 22.54 | 2 | 3400 | 48 | 4 | 4 | 1 | 1 | 95.5 | 13.525 | 13.8 | 231.13 | 10.75 | 29.51 | 62.72 | 0.99 | 5 |
| 5 | 9.95 | 2 | 3800 |  | 5 | 5 | 2 | 1 | 71.5 | 9.605 | 9.9 |  |  |  |  |  | 6 |
| 6 | 23.46 | 1 | 2550 | 49 | 3 | 2 | 1 | 1 | 81 | 8.89 | 13.8 | 186.96 | 11.10 | 24.37 | 79.27 |  | 2 |
| 7 | 12.25 | 1 | 3750 |  | 6 | 4 | 2 | 2 | 75.5 | 8.945 | 10.6 |  |  |  |  |  | 4 |
| 8 | 9.76 | 1 | 3500 | 50 | 3 | 2 | 1 | 1 | 70 | 8.105 | 10.1 | 257.01 | 16.22 | 5.24 | 81.23 | 0.94 | 0 |
| 9 | 34.4 | 1 | 3700 | 49 | 3 | 3 | 1 | 1 | 95 | 14.555 | 13 | 425.11 | 14.76 | 44.17 | 73.86 |  | 4 |
| 10 | 16.16 | 1 | 3250 | 51 | 1 | 1 | 2 | 1 | 76 | 10.42 | 12.1 | 130.29 | 7.82 | 13.38 | 53.17 | 1.05 | 2 |
| 11 | 7 | 1 | 3350 |  | 6 | 6 | 1 | 1 | 70 | 7.99 | 11 |  |  |  |  |  | 5 |
| 12 | 2.69 | 2 | 2650 | 50 | 5 | 3 | 1 | 2 | 60.5 | 5.66 | 11.6 |  |  |  |  |  | 0 |
| 13 | 18.89 | 1 | 3600 | 50 | 4 | 6 | 1 | 1 | 80.5 | 10.3 | 13.1 | 368.63 | 36.81 | 32.43 | 77.70 | 0.88 | 5 |
| 14 | 3.84 | 1 | 3135 | 51 | 4 | 4 | 2 | 1 | 65 | 6.805 | 8.2 |  |  |  |  |  | 0 |
| 15 | 16.59 | 1 | 4000 | 51 | 4 | 3 | 1 | 1 | 80 | 9.821 | 11.3 | 160.47 | 11.74 | 8.42 | 68.29 | 1.00 | 2 |
| 16 | 32.92 | 2 | 3050 |  | 2 | 3 | 1 | 1 | 91 | 13.385 | 12.4 | 266.26 | 13.11 | 39.19 | 100.98 |  | 3 |
| 17 | 2.96 | 1 | 2600 |  | 3 | 4 | 1 | 1 | 59.5 | 5.18 | 11.1 |  |  |  |  |  | 1 |
| 18 | 2.96 | 1 | 2650 |  | 3 | 4 | 1 | 2 | 59.5 | 5.58 | 13.4 |  |  |  |  |  | 0 |
| 19 | 3.02 | 2 | 2800 | 52 | 5 | 6 | 1 | 1 | 61.5 | 5.75 | 10.8 |  |  |  |  |  | 0 |
| 20 | 17.54 | 2 | 3050 | 50 | 3 | 2 | 1 | 1 | 78 | 8.84 | 12.6 | 537.37 | 8.46 | 11.89 | 73.86 | 1.00 | 4 |
| 21 | 13.54 | 1 | 3000 | 50 | 3 | 3 | 1 | 1 | 76.6 | 9.5 | 11 |  |  |  |  |  | 6 |
| 22 | 8.9 | 2 | 3400 | 50 | 3 | 3 | 2 | 1 | 71.1 | 8.385 | 11.9 |  |  |  |  |  | 5 |
| 23 | 32.85 | 1 | 3000 | 50 | 6 | 5 | 2 | 1 | 89 | 12.295 | 9.9 | 572.19 | 17.48 | 39.38 | 55.97 |  | 5 |
| 24 | 24.21 | 1 | 3300 | 50 | 6 | 2 | 1 | 1 | 84.3 | 10.19 | 11.8 | 200.36 | 6.57 | 16.98 | 76.19 |  | 1 |
| 25 | 35.22 | 2 |  |  | 3 | 3 | 1 | 1 | 93 | 14.6 | 13.6 | 190.95 | 7.96 | 21.98 | 61.44 | 0.79 | 3 |
| 26 | 0.49 | 2 | 3400 | 51 | 6 | 4 | 1 | 1 | 53 | 4.41 | 15.9 |  |  |  |  |  | 0 |
| 27 | 25.59 | 2 | 3000 | 50 | 4 | 3 | 1 | 1 | 93 | 10.85 | 11.1 | 239.30 | 32.75 | 44.90 | 63.37 |  | 1 |
| 28 | 20.01 | 1 | 3300 | 50 | 5 | 4 | 2 | 1 | 81 | 9.87 | 11.9 |  |  | 50.83 | 45.78 |  | 4 |
| 29 | 4.01 | 1 | 2900 |  | 2 | 3 | 1 | 1 | 62 | 5.915 | 8.3 |  |  |  |  |  | 0 |
| 30 | 6.83 | 1 | 3800 | 52 | 4 | 2 | 1 | 1 | 71 | 9.3 | 11.5 |  |  |  |  |  | 2 |
| 31 | 7.95 | 1 | 3200 | 50 | 4 | 6 | 1 | 1 | 70.5 | 7.45 | 12.1 |  |  |  |  |  | 4 |
| 32 | 1.84 | 2 | 2750 | 50 | 4 | 2 | 2 | 2 | 55.5 | 4.505 | 9.6 |  |  |  |  |  | 0 |
| 33 | 10.25 | 1 | 3600 | 52 | 5 | 6 | 1 | 1 | 77 | 9.8 | 11.6 |  |  |  |  |  | 3 |
| 34 | 8.21 | 1 | 3770 | 52 | 6 | 6 | 2 | 2 | 72.5 | 8.79 | 11.7 |  |  |  |  |  | 4 |
| 35 | 18.73 | 1 | 3000 | 50 | 4 | 5 | 1 | 1 | 82 | 10.35 | 11 | 270.91 | 5.84 | 9.50 | 72.20 |  | 5 |
| 36 | 19.91 | 2 | 3100 | 50 | 4 | 5 | 1 | 1 | 85.5 | 9.86 | 10 | 442.62 | 30.64 | 48.41 | 94.10 |  | 1 |
| 37 | 17.28 | 2 | 3200 | 50 | 3 | 3 | 1 | 2 | 80 | 10.51 | 12.3 | 294.91 | 5.58 | 19.46 | 76.19 |  | 4 |
| 38 | 14.55 | 1 | 3300 | 51 | 4 | 4 | 2 | 1 | 80 | 9.201 | 13.1 |  |  |  |  |  | 1 |
| 39 | 10.22 | 2 | 2800 | 50 | 6 | 6 | 1 | 1 | 72 | 8.86 | 10.7 |  |  |  |  |  | 3 |
| 40 | 27.76 | 1 | 3750 | 53 | 5 | 6 | 1 | 1 | 88.5 | 12.15 | 13.4 | 709.50 | 32.49 | 47.44 | 78.96 |  | 4 |
| 41 | 25.92 | 2 | 3200 | 50 | 6 | 6 | 1 | 1 | 89 | 11.73 | 14.4 | 465.15 | 35.73 | 22.91 | 65.64 | 1.07 | 6 |
| 42 | 15.54 | 2 | 2800 | 50 | 6 | 6 | 1 | 1 | 79.5 | 9.812 | 14 | 474.58 | 16.29 | 87.19 | 77.70 |  | 4 |
| 43 | 24.02 | 1 | 2250 | 48 | 3 | 3 | 1 | 1 | 81 | 11.27 | 12.6 | 188.18 | 9.54 | 10.32 | 46.60 |  | 4 |
| 44 | 15.64 | 1 | 3000 | 50 | 3 | 3 | 1 | 1 | 79.2 | 8.99 | 12.7 |  |  | 12.06 | 67.80 |  | 3 |
| 45 | 26.15 | 1 | 2950 |  | 3 | 3 | 1 | 1 | 84.2 | 10.16 | 13.1 |  |  |  |  |  | 2 |
| 46 | 7.06 | 2 | 3500 | 50 | 3 | 5 | 1 | 2 | 68 | 7.4 | 11.3 |  |  |  |  |  | 2 |
| 47 | 33.08 | 2 | 3000 | 50 | 4 | 3 | 1 | 1 | 92 | 12.94 | 12.1 | 214.38 | 6.06 | 46.19 | 52.67 |  | 5 |
| 48 | 5.88 | 2 | 2800 | 50 | 3 | 3 | 1 | 2 | 64 | 7.485 | 10.8 |  |  |  |  |  | 3 |
| 49 | 11.86 | 2 | 3500 | 42 | 6 | 5 | 1 | 1 | 73 | 8.505 | 12.8 | 336.11 | 15.86 | 136.57 | 65.64 |  | 6 |
| 50 | 9.23 | 2 | 3150 | 50 | 5 | 4 | 1 | 2 | 72 | 10.15 | 12 |  |  |  |  |  | 2 |
| 51 | 31.01 | 1 |  |  | 3 | 2 | 1 | 1 | 87 | 12 | 11.4 | 579.11 | 5.86 | 61.14 | 68.79 |  | 3 |
| 52 | 10.12 | 2 | 2500 | 49 | 4 | 3 | 2 | 1 | 71 | 6.68 | 11.6 |  |  |  |  |  | 3 |
| 53 | 18.92 | 2 | 3100 | 52 | 6 | 2 | 1 | 1 | 77 | 9.65 | 13.9 | 451.02 | 28.74 | 18.87 | 78.64 |  | 4 |
| 54 | 25.76 | 2 | 3200 |  | 4 | 4 | 1 | 1 | 86.5 | 11.7 | 13.6 | 179.44 | 6.42 | 14.65 | 65.87 |  | 5 |
| 55 | 10.97 | 2 | 2600 | 51 | 3 | 2 | 1 | 1 | 69 | 6.94 | 11.1 |  |  |  |  |  | 5 |
| 56 | 8.57 | 2 | 3700 | 51 | 3 | 3 | 1 | 1 | 75.5 | 8.28 | 10.7 |  |  |  |  |  | 3 |
| 57 | 21.49 | 1 | 3500 | 48 | 5 | 6 | 1 | 1 | 85.5 | 11.74 | 11.5 |  |  |  |  |  | 7 |
| 58 | 12.52 | 2 | 3200 | 50 | 6 | 6 | 1 | 1 | 74.2 | 8.88 | 11.8 | 800.15 | 42.01 | 28.29 | 72.47 |  | 2 |
| 59 | 3.58 | 2 | 3100 | 50 | 4 | 3 | 1 | 2 | 61 | 5.395 | 12.7 |  |  |  |  |  | 1 |
| 60 | 13.9 | 1 | 3000 | 50 | 5 | 3 | 1 | 2 | 74.8 | 8.53 | 12.3 |  |  |  |  |  | 5 |
| 61 | 14.29 | 1 | 2700 | 50 | 4 | 3 | 1 | 1 | 75 | 9.2 | 11.3 |  |  |  |  |  | 2 |
| 62 | 30.13 | 2 | 2900 |  | 4 | 6 | 1 | 1 | 86 | 10.325 | 13.6 | 666.72 | 10.04 | 32.46 | 59.00 |  | 4 |
| 63 | 2.5 | 2 | 2900 |  | 5 | 4 | 1 | 2 | 60.2 | 6 | 10.5 |  |  |  |  |  | 1 |
| 64 | 8.28 | 1 | 3000 | 50 | 4 | 5 | 1 | 1 | 69 | 9.09 | 11.6 |  |  |  |  |  | 6 |
| 65 | 3.88 | 1 | 3500 | 50 | 6 | 6 | 1 | 2 | 67 | 7.16 | 9.7 |  |  |  |  |  | 1 |
| 66 | 28.42 | 2 |  |  | 4 | 3 | 2 | 1 | 83.5 | 10.87 | 12.8 | 528.13 | 7.98 | 46.50 | 68.54 | 0.79 | 4 |
| 67 | 10.61 | 1 | 3250 | 50 | 4 | 4 | 2 | 2 | 72 | 7.36 | 10.9 |  |  |  |  |  | 3 |
| 68 | 24.31 | 2 | 3700 |  | 6 | 6 | 1 | 1 | 86 | 13.03 | 12.5 | 280.89 | 34.34 | 42.19 | 69.30 |  | 7 |
| 69 | 16 | 1 |  |  | 3 | 3 | 2 | 2 | 79.4 | 11.36 | 10.4 | 474.58 | 12.27 | 37.61 | 83.27 | 0.93 | 3 |
| 70 | 27.76 | 1 | 3050 | 50 | 4 | 5 | 1 | 1 | 88.5 | 13.14 | 14.6 |  |  |  |  |  | 6 |
| 71 | 8.54 | 2 | 1500 |  | 4 | 4 | 2 | 1 | 68 | 7.405 | 7.3 |  |  |  |  |  | 2 |
| 72 | 16.99 | 1 | 3200 |  | 4 | 3 | 2 | 1 | 80 | 10.18 | 11.8 | 227.99 | 5.56 | 23.40 | 63.37 |  | 3 |
| 73 | 32.16 | 1 | 3500 |  | 6 | 3 | 2 | 1 | 97 | 15.355 | 13.5 | 255.88 | 5.05 | 68.86 | 59.00 |  | 0 |
| 74 | 18.92 | 1 | 3200 | 50 | 6 | 3 | 1 | 1 | 80 | 9.185 | 10.9 |  |  |  |  |  | 4 |
| 75 | 13.34 | 1 | 3000 |  | 6 | 5 | 1 | 1 | 78 | 9.5 | 11.7 |  |  |  |  |  | 5 |
| 76 | 6.51 | 1 | 3000 | 52 | 3 | 4 | 1 | 2 | 65 | 8.75 | 10.2 |  |  |  |  |  | 2 |
| 77 | 17.12 | 2 | 3300 |  | 3 | 3 | 1 | 1 | 78 | 10.1 | 11.5 |  |  |  |  |  | 6 |
| 78 | 32.2 | 2 | 2000 |  | 3 | 3 | 1 | 1 | 83.3 | 10.665 | 12.1 |  |  |  |  |  | 3 |
| 79 | 11.6 | 1 | 3000 | 50 | 4 | 5 | 1 | 1 | 76 | 8.54 | 12.7 |  |  |  |  |  | 6 |
| 80 | 34.53 | 2 | 2650 | 50 | 6 | 5 | 1 | 1 | 98 | 12.37 | 12.2 | 253.62 | 11.49 | 63.32 | 71.65 |  | 6 |
| 81 | 5.09 | 1 | 3100 |  | 4 | 3 | 1 | 1 | 66 | 6.64 | 12.2 |  |  |  |  |  | 4 |
| 82 | 10.78 | 1 | 3300 |  | 5 | 6 | 1 | 2 | 74 | 8.7 | 13.1 |  |  |  |  |  | 5 |
| 83 | 26.22 | 1 | 3900 |  | 4 | 3 | 1 | 1 | 88 | 12.95 | 13.3 |  |  |  |  |  | 3 |
| 84 | 12.09 | 2 | 3400 | 55 | 6 | 6 | 1 | 2 | 75 | 9.9 | 9.1 |  |  |  |  |  | 5 |
| 85 | 29.47 | 2 | 3550 | 50 | 6 | 6 | 1 | 1 | 89 | 13.7 | 13 | 409.34 | 9.70 | 61.74 | 44.33 |  | 5 |
| 86 | 11.14 | 1 | 2650 |  | 3 | 3 | 1 | 1 | 71 | 10.715 | 9.8 |  |  |  |  |  | 6 |
| 87 | 9.99 | 2 | 2350 |  | 6 | 4 | 1 | 2 | 71 | 7.9 | 9.7 |  |  |  |  |  | 4 |
| 88 | 11.89 | 1 | 2400 |  | 6 | 6 | 2 | 2 | 74.2 | 8.445 | 11.4 |  |  |  |  |  | 5 |
| 89 | 18.56 | 2 | 3000 |  | 6 | 4 | 1 | 2 | 80 | 9.275 | 13.7 |  |  |  |  |  | 3 |
| 90 | 29.27 | 2 | 2350 | 49 | 3 | 3 | 1 | 1 | 82 | 9.9 | 13.4 | 374.22 | 14.92 | 43.81 | 44.19 |  | 3 |
| 91 | 28.68 | 2 | 3000 |  | 3 | 3 | 2 | 1 | 81.1 | 10.02 | 11.7 | 325.97 | 19.06 | 107.81 | 66.34 |  | 3 |
| 92 | 10.28 | 2 | 4150 | 53 | 3 | 4 | 1 | 1 | 72 | 9.23 | 11 |  |  |  |  |  | 3 |
| 93 | 12.62 | 1 | 4050 | 52 | 6 | 4 | 1 | 1 | 76 | 8.61 | 11 | 855.15 | 41.28 | 25.67 | 73.30 | 1.10 | 4 |
| 94 | 35.94 | 2 | 2800 |  | 4 | 4 | 1 | 1 | 87 | 12.545 | 13.2 | 510.11 | 13.39 | 46.72 | 67.55 | 1.08 | 7 |
| 95 | 26.74 | 1 | 2700 |  | 4 | 4 | 1 | 1 | 87.5 | 11.62 | 12.6 |  |  |  |  |  | 5 |
| 96 | 24.97 | 2 | 2700 |  | 4 | 3 | 1 | 1 | 81.5 | 9.06 | 13.2 |  |  |  |  |  | 6 |
| 97 | 10.71 | 1 | 2600 | 48 | 6 | 4 | 2 | 1 | 75 | 8.165 | 12.6 |  |  |  |  |  | 5 |
| 98 | 21.16 | 1 | 3400 |  | 6 | 6 | 2 | 1 | 91 | 13.86 | 14.2 |  |  |  |  |  | 4 |
| 99 | 9.82 | 1 | 3400 |  | 3 | 3 | 1 | 1 | 71 | 8.025 | 11.2 |  |  |  |  |  | 4 |
| 100 | 29.37 | 1 | 4000 | 44 | 5 | 6 | 1 | 1 | 91 | 14.185 | 11.1 |  |  |  |  |  | 7 |
| 101 | 22.97 | 2 | 3600 |  | 6 | 6 | 1 | 1 | 89 | 11.61 | 12.7 | 402.20 | 12.14 | 23.83 | 63.15 | 1.23 | 7 |
| 102 | 13.4 | 2 | 3450 |  | 3 | 4 | 1 | 2 | 75.5 | 9.46 | 11.4 |  |  |  |  |  | 5 |
| 103 | 32.59 | 1 | 4100 |  | 6 | 4 | 1 | 1 | 92.5 | 14.01 | 11.5 | 348.91 | 6.95 | 30.79 | 61.86 |  | 4 |
| 104 | 16.16 | 1 | 3300 |  | 6 | 4 | 1 | 1 | 80.4 | 10.5 | 11.9 | 506.03 | 24.83 | 29.98 | 87.63 | 1.37 | 5 |
| 105 | 7.56 | 2 | 3000 | 50 | 6 | 4 | 1 | 1 | 66.9 | 7.24 | 12.4 |  |  |  |  |  | 5 |
| 106 | 9.53 | 2 | 2700 | 50 | 3 | 3 | 1 | 1 | 67 | 7.505 | 11.7 |  |  |  |  |  | 4 |
| 107 | 9.2 | 1 | 2650 | 53 | 5 | 6 | 1 | 1 | 70.5 | 9.27 | 12.2 |  |  |  |  |  | 6 |
| 108 | 27.24 | 1 | 3300 | 54 | 5 | 5 | 1 | 1 | 87.5 | 13.35 | 12.2 | 604.44 | 17.35 | 23.05 | 64.72 |  | 3 |
| 109 | 18.66 | 2 | 3400 | 50 | 5 | 5 | 2 | 1 | 81.5 | 10.65 | 13 |  |  |  |  |  | 4 |
| 110 | 16.07 | 2 | 3000 |  | 6 | 6 | 1 | 1 | 81 | 9.605 | 12.1 |  |  |  |  |  | 5 |
| 111 | 34.63 | 1 |  |  | 6 | 4 | 1 | 1 | 101.1 | 16 | 13.3 | 390.90 | 3.17 | 24.48 | 68.05 |  | 5 |
| 112 | 2.07 | 2 | 3300 | 52 | 6 | 6 | 1 | 1 | 60 | 4.91 | 10 |  |  |  |  |  | 1 |
| 113 | 3.78 | 1 | 3800 | 50 | 6 | 5 | 1 | 1 | 62 | 6.485 | 11.1 |  |  |  |  |  | 2 |
| 114 | 21.59 | 2 | 3000 |  | 3 | 3 | 2 | 1 | 82 | 10.672 | 11.3 |  |  |  |  |  | 2 |
| 115 | 1.81 | 2 | 3000 | 50 | 3 | 3 | 2 | 1 | 56 | 4.388 | 12 |  |  |  |  |  | 0 |
| 116 | 6.7 | 2 | 3000 | 50 | 3 | 3 | 2 | 1 | 68 | 8.672 | 10.3 |  |  |  |  |  | 0 |
| 117 | 8.61 | 1 | 3200 |  | 3 | 3 | 1 | 1 | 66 | 7.715 | 10.3 |  |  |  |  |  | 4 |
| 118 | 26.35 | 1 |  |  | 3 | 3 | 1 | 1 | 82 | 10.205 | 11.8 |  |  |  |  |  | 3 |
| 119 | 7 | 1 | 3750 | 63 | 4 | 4 | 1 | 2 | 76 | 9.45 | 11.3 |  |  |  |  |  | 2 |
| 120 | 1.94 | 1 | 3900 | 50 | 4 | 3 | 1 | 2 | 56.4 | 6.08 | 10.6 |  |  |  |  |  | 1 |
| 121 | 14.03 | 1 | 3000 | 50 | 4 | 3 | 2 | 1 | 73.4 | 7.96 | 10.6 |  |  |  |  |  | 4 |
| 122 | 8.51 | 2 |  |  | 3 | 3 | 2 | 1 | 67 | 8.25 | 10.6 |  |  |  |  |  | 3 |
| 123 | 12.06 | 1 | 3400 |  | 2 | 3 | 1 | 1 | 74.4 | 6.85 | 12.9 |  |  |  |  |  | 5 |
| 124 | 14.95 | 2 | 3150 | 50 | 3 | 3 | 1 | 1 | 78.3 | 7.99 | 10.7 |  |  |  |  |  | 4 |
| 125 | 23.79 | 2 |  |  | 3 | 4 | 2 | 1 | 78.4 | 9.105 | 10.6 |  |  |  |  |  | 2 |
| 126 | 5.85 | 2 |  |  | 3 | 4 | 1 | 1 | 81.3 | 9.825 | 12.3 |  |  |  |  |  | 3 |
| 127 | 10.32 | 1 | 3600 | 51 | 3 | 3 | 1 | 1 | 73 | 8.75 | 10.6 |  |  |  |  |  | 5 |
| 128 | 6.08 | 1 | 3100 | 50 | 3 | 3 | 1 | 2 | 66.4 | 6.81 | 8.8 |  |  |  |  |  | 0 |
| 129 | 11.6 | 2 |  |  | 3 | 3 | 1 | 1 | 71.5 | 8.27 | 11.3 |  |  |  |  |  | 3 |
| 130 | 25.46 | 1 | 3500 | 50 | 3 | 3 | 1 | 1 | 82 | 11.265 | 11 | 463.28 | 18.59 | 10.77 | 60.97 | 0.91 | 6 |
| 131 | 30.16 | 2 | 3800 | 52 | 3 | 3 | 1 | 1 | 64 | 9.005 | 11.3 |  |  |  |  |  | 0 |
| 132 | 17.38 | 2 | 3500 |  | 6 | 3 | 1 | 1 | 79 | 9.575 | 10.2 |  |  |  |  |  | 6 |
| 133 | 14.03 | 2 | 3500 | 50 | 3 | 3 | 1 | 1 | 74 | 8.2 | 9.8 |  |  |  |  |  | 4 |
| 134 | 16.89 | 1 | 3300 |  | 4 | 6 | 1 | 1 | 81 | 11.785 | 12.8 |  | 32.70 | 74.72 | 86.23 | 0.91 | 2 |
| 135 | 32.59 | 1 |  |  | 4 | 3 | 1 | 1 | 82 | 11.9 | 11.7 |  |  |  |  |  | 3 |
| 136 | 16.66 | 2 |  |  | 3 | 3 | 1 | 1 | 76 | 7.565 | 9.6 |  |  |  |  |  | 5 |
| 137 | 15.74 | 1 | 4000 |  | 3 | 3 | 2 | 1 | 78 | 9.945 | 11.3 | 302.12 | 11.03 | 24.45 | 53.69 | 1.08 | 1 |
| 138 | 26.81 | 1 | 3400 |  | 4 | 5 | 1 | 1 | 91.5 | 10.98 | 13.8 |  | 17.25 | 36.39 | 73.37 |  | 5 |
| 139 | 16.99 | 2 | 3000 |  | 3 | 3 | 1 | 1 | 86 | 11.93 | 10.8 | 180.03 | 16.05 | 8.47 | 76.46 | 1.13 | 3 |
| 140 | 0.85 | 1 |  |  | 3 | 3 | 1 | 1 | 53.5 | 4.115 | 13.7 |  |  |  |  |  | 0 |
| 141 | 19.78 | 1 |  |  | 3 | 3 | 1 | 1 | 77 | 9.48 | 11.2 |  |  |  |  |  | 6 |
| 142 | 20.11 | 2 | 3600 | 50 | 4 | 3 | 1 | 1 | 78.5 | 9.715 | 11.4 |  |  |  |  |  | 4 |
| 143 | 2.2 | 2 | 3200 | 50 | 4 | 3 | 1 | 1 | 56.4 | 5.85 | 10.5 |  |  |  |  |  | 0 |
| 144 | 30.59 | 2 | 3000 |  | 4 | 3 | 1 | 1 | 85.3 | 10.885 | 12.9 |  |  |  |  |  | 5 |
| 145 | 7.72 | 2 | 2400 |  | 6 | 3 | 1 | 1 | 67.3 | 7.48 | 11.9 |  |  |  |  |  | 3 |
| 146 | 31.87 | 2 | 3300 |  | 4 | 3 | 2 | 1 | 87 | 13.225 | 12.6 |  |  |  |  |  | 3 |
| 147 | 11.43 | 1 | 3000 |  | 2 | 3 | 2 | 1 | 75 | 9.505 | 11.8 |  |  |  |  |  | 3 |
| 148 | 31.7 | 2 | 3500 |  | 2 | 4 | 1 | 1 | 90 | 11.5 | 13.2 | 344.60 | 7.61 | 24.65 | 55.38 | 1.09 | 1 |
| 149 | 28.42 | 1 | 3000 |  | 2 | 2 | 1 | 1 | 85.2 | 10.735 | 10.9 | 135.04 | 5.16 | 35.51 | 52.47 |  | 1 |
| 150 | 7.13 | 2 | 3900 |  | 4 | 3 | 1 | 1 | 67 | 7.462 | 12 |  |  |  |  |  | 2 |
| 151 | 36.01 | 1 | 3500 |  | 3 | 3 | 1 | 1 | 86.4 | 10.885 | 11.6 | 258.53 | 19.48 | 14.76 | 45.42 | 1.11 | 0 |
| 152 | 29.6 | 2 | 3000 |  | 5 | 5 | 2 | 1 | 83 | 11.95 | 11.2 |  |  |  |  |  | 1 |
| 153 | 30.55 | 1 | 3300 |  | 2 | 2 | 1 | 1 | 82 | 11.435 | 12.6 |  |  |  |  |  | 2 |
| 154 | 16.95 | 2 | 3100 |  | 5 | 5 | 2 | 1 | 82 | 9.87 | 11.3 |  |  |  |  |  | 4 |
| 155 | 13.77 | 2 | 2600 | 47 | 3 | 3 | 1 | 1 | 70.4 | 7.725 | 11.9 |  |  |  |  |  | 5 |
| 156 | 13.77 | 2 | 2600 | 47 | 3 | 3 | 1 | 1 | 70 | 7.075 | 10.2 |  |  |  |  |  | 5 |
| 157 | 29.77 | 1 | 2800 |  | 6 | 6 | 2 | 1 | 86.3 | 12.835 | 10.8 |  |  |  |  |  | 5 |
| 158 | 28.25 | 2 | 3250 |  | 2 | 3 | 1 | 1 | 84 | 10.75 | 14 |  |  |  |  |  | 4 |
| 159 | 8.08 | 2 | 2750 | 46 | 4 | 2 | 1 | 1 | 65.8 | 6.43 | 11.7 |  |  |  |  |  | 3 |
| 160 | 32.26 | 2 | 3000 | 50 | 3 | 4 | 1 | 1 | 84.2 | 10.65 | 13.1 | 269.73 | 12.81 | 25.68 | 56.93 |  | 5 |
| 161 | 21.22 | 1 | 3600 | 50 | 3 | 1 | 1 | 1 | 79.3 | 10.625 | 9.2 |  |  |  |  |  | 2 |
| 162 | 20.44 | 2 | 3000 | 50 | 3 | 3 | 1 | 1 | 76 | 9.03 | 12 |  |  |  |  |  | 4 |
| 163 | 20.21 | 1 | 3500 | 41 | 2 | 1 | 1 | 1 | 78 | 10.295 | 10.5 |  |  |  |  |  | 4 |
| 164 | 12.81 | 1 | 3200 | 40 | 3 | 3 | 1 | 1 | 72.8 | 8.95 | 10.6 |  |  |  |  |  | 3 |
| 165 | 10.61 | 2 | 2800 |  | 3 | 2 | 2 | 1 | 70.5 | 8.945 | 9.6 |  |  |  |  |  | 5 |
| 166 | 16.82 | 2 |  |  | 3 | 2 | 1 | 1 | 75 | 8.93 | 11.1 |  |  |  |  |  | 3 |
| 167 | 10.15 | 2 | 3000 | 50 | 4 | 3 | 1 | 2 | 68 | 7.035 | 9.5 |  |  |  |  |  | 3 |
| 168 | 19.98 | 2 | 3500 |  | 2 | 3 | 1 | 1 | 80 | 9.74 | 11.9 | 187.88 | 8.14 | 16.61 | 34.80 |  | 3 |
| 169 | 11.01 | 2 | 4000 |  | 5 | 4 | 1 | 1 | 71.6 | 8.92 | 11.7 |  |  |  |  |  | 5 |
| 170 | 13.11 | 2 | 3150 | 49 | 3 | 3 | 2 | 1 | 77 | 9.25 | 10.2 |  |  |  |  |  | 7 |
| 171 | 33.64 | 1 |  |  | 2 | 2 | 1 | 1 | 83.4 | 10.07 | 12.4 | 229.38 | 4.82 | 15.57 | 43.83 |  | 3 |
| 172 | 19.48 | 1 |  |  | 3 | 2 | 1 | 1 | 81.3 | 10.355 | 9.5 |  |  |  |  |  | 6 |
| 173 | 20.21 | 2 | 3300 | 50 | 2 | 3 | 1 | 1 | 78 | 9.28 | 11.6 |  |  |  |  |  | 4 |
| 174 | 20.67 | 1 | 3800 |  | 3 | 2 | 1 | 1 | 80 | 10.09 | 10.2 |  |  |  |  |  | 2 |
| 175 | 7 | 2 | 3250 | 50 | 4 | 4 | 1 | 1 | 68 | 8.1 | 11.8 |  |  |  |  |  | 1 |
| 176 | 22.41 | 2 | 2900 |  | 5 | 4 | 1 | 1 | 75 | 8.045 | 13.5 |  |  |  |  |  | 5 |
| 177 | 20.27 | 1 |  |  | 6 | 3 | 1 | 1 | 81 | 9.846 | 11.7 |  |  |  |  |  | 6 |
| 178 | 11.01 | 2 | 2750 |  | 6 | 6 | 2 | 1 | 72.1 | 8.525 | 8.3 |  |  |  |  |  | 0 |
| 179 | 10.41 | 2 | 3250 |  | 4 | 2 | 1 | 1 | 70 | 8.505 | 9.7 |  |  |  |  |  | 4 |
| 180 | 11.79 | 1 | 3700 | 51 | 5 | 5 | 1 | 1 | 73 | 9.61 | 10.9 |  |  |  |  |  | 4 |
| 181 | 7.69 | 1 | 3000 |  | 3 | 3 | 1 | 1 | 69 | 8.53 | 11.7 |  |  |  |  |  | 3 |
| 182 | 1.64 | 1 | 3600 | 55 | 4 | 2 | 1 | 1 | 57 | 4.805 | 11.8 |  |  |  |  |  | 0 |
| 183 | 11.53 | 2 | 2700 |  | 3 | 3 | 1 | 1 | 71.1 | 7.895 | 12.4 |  |  |  |  |  | 2 |
| 184 | 13.7 | 1 | 3400 |  | 6 | 4 | 2 | 2 | 78 | 11.245 | 12.4 |  |  |  |  |  | 5 |
| 185 | 7.66 | 1 | 4100 |  |  |  |  |  | 68 | 7.959 | 12.4 |  |  |  |  |  | 0 |
| 186 | 18.14 | 1 |  |  | 4 | 4 | 1 | 1 | 85 | 10.95 | 13.7 |  |  |  |  |  | 5 |
| 187 | 7.69 | 1 | 4000 |  | 3 | 3 | 1 | 1 | 65 | 9.06 | 11.9 |  |  |  |  |  | 3 |
| 188 | 20.67 | 2 |  |  | 2 | 2 | 1 | 1 | 78 | 9.075 | 11.5 |  |  |  |  |  | 5 |
| 189 | 15.15 | 2 | 3250 |  | 3 | 2 | 1 | 1 | 79 | 10.4 | 10.6 |  |  |  |  |  | 4 |
| 190 | 13.27 | 1 | 3250 |  |  |  |  |  | 73.2 | 9.335 | 11 |  |  |  |  |  | 0 |
| 191 | 16.16 | 1 | 3450 |  | 3 | 3 | 1 | 1 | 78.5 | 10.785 | 10.6 |  |  |  |  |  | 4 |
| 192 | 3.65 | 1 | 3400 | 51 | 3 | 2 | 1 | 1 | 58 | 6.65 | 10.7 |  |  |  |  |  | 0 |
| 193 | 4.83 | 2 | 3200 | 50 | 4 | 3 | 1 | 1 | 65 | 6.665 | 11.1 |  |  |  |  |  | 2 |
| 194 | 1.68 | 2 | 3400 | 51 | 3 |  | 1 | 1 | 56 | 5.235 | 9.8 |  |  |  |  |  | 1 |
| 195 | 4.3 | 1 | 3000 |  | 3 | 3 | 1 | 1 | 64 | 7.235 | 11.1 |  |  |  |  |  | 1 |
| 196 | 14.06 | 1 | 3350 |  | 2 | 2 | 1 | 1 | 75 | 9.985 | 11.5 |  |  |  |  |  | 4 |
| 197 | 7.03 | 1 | 2150 |  | 3 | 2 | 1 | 1 | 63 | 6.59 | 11.4 |  |  |  |  |  | 2 |
| 198 | 7.03 | 1 | 2000 |  | 3 | 2 | 1 | 1 | 63 | 6.48 | 11.8 |  |  |  |  |  | 2 |
| 199 | 29.96 | 2 | 3500 | 50 |  |  |  |  | 84.5 | 10.015 | 12.3 |  |  |  |  |  | 0 |
| 200 | 14.42 | 1 | 3500 | 50 | 2 | 1 | 1 | 1 | 75 | 9.12 | 11 |  |  |  |  |  | 2 |
| 201 | 15.34 | 2 | 3750 |  | 4 | 4 | 1 | 2 | 78 | 9.95 | 11.1 |  |  |  |  |  | 2 |
| 202 | 6.01 | 1 | 3500 |  | 3 | 3 | 1 | 1 | 67 | 8.86 | 11.1 |  |  |  |  |  | 2 |
| 203 | 16.13 | 2 | 3250 |  | 3 | 2 | 1 | 1 | 77.5 | 9 | 10.7 |  |  |  |  |  | 5 |
| 204 | 10.61 | 1 |  |  | 3 | 2 | 1 | 1 | 69 | 8.23 | 11.5 |  |  |  |  |  | 3 |
| 205 | 31.87 | 2 | 2750 |  |  |  |  |  | 83.5 | 10.43 | 13.5 |  |  |  |  |  | 0 |
| 206 | 11.93 | 1 |  |  | 3 | 3 | 1 | 1 | 68 | 7.895 | 10.3 |  |  |  |  |  | 4 |
| 207 | 31.57 | 1 |  |  |  |  |  |  | 93 | 11.5 | 11.8 |  |  |  |  |  | 0 |
| 208 | 3.71 | 1 | 2500 |  | 2 | 2 | 1 | 1 | 59 | 4.515 | 10.5 |  |  |  |  |  | 1 |
| 209 | 16.69 | 2 | 3050 |  | 3 | 2 | 1 | 1 | 80 | 10.41 | 10.4 |  |  |  |  |  | 4 |
| 210 | 1.54 | 2 | 3200 | 50 | 3 | 3 | 1 | 1 | 58.5 | 6.35 | 11.4 |  |  |  |  |  | 0 |
| 211 | 23.62 | 1 | 3700 | 51 | 3 | 3 | 1 | 1 | 86 | 11.55 | 12.1 |  |  |  |  |  | 4 |
| 212 | 17.58 | 1 | 3450 | 50 | 2 | 2 | 1 | 2 | 77 | 9.245 | 10.7 |  |  |  |  |  | 4 |
| 213 | 10.48 | 1 | 3000 |  | 2 | 2 | 1 | 1 | 65 | 8.48 | 11.1 |  |  |  |  |  | 3 |
| 214 | 15.31 | 1 | 3500 |  | 3 | 2 | 1 | 1 | 75 | 9.4 | 12.1 |  |  |  |  |  | 3 |
| 215 | 17.18 | 2 | 2400 |  | 3 | 3 | 1 | 1 | 74.1 | 8.37 | 11.9 |  |  |  |  |  | 5 |
| 216 | 23.69 | 2 | 3000 |  |  |  |  |  | 81 | 9.66 | 12.2 |  |  |  |  |  | 0 |
| 217 | 33.41 | 1 | 3550 | 51 |  |  |  |  | 92 | 13.01 | 11.8 |  |  |  |  |  | 0 |
| 218 | 12.98 | 2 | 3000 |  | 3 | 3 | 2 | 1 | 74 | 8.615 | 10.3 |  |  |  |  |  | 2 |
| 219 | 13.86 | 2 |  |  | 3 | 1 | 1 | 1 | 70 | 8.395 | 11.4 |  |  |  |  |  | 3 |
| 220 | 27.86 | 2 | 4750 |  |  |  |  |  | 91 | 14.03 | 11.9 | 248.78 | 3.37 | 14.85 | 50.84 | 0.89 | 0 |
| 221 | 9.53 | 1 | 3600 |  | 3 | 3 | 2 | 1 | 68 | 9.09 | 9.6 |  |  |  |  |  | 5 |
| 222 | 28.71 | 2 | 3500 |  |  |  |  |  | 84.5 | 10.71 | 11.5 |  |  |  |  |  | 0 |
| 223 | 10.02 | 2 | 3300 |  | 4 | 3 | 1 | 1 | 69 | 7.755 | 10.7 |  |  |  |  |  | 5 |
| 224 | 1.08 | 2 | 3900 |  | 2 | 2 | 1 | 1 | 56 | 4.565 | 10.1 |  |  |  |  |  | 0 |
| 225 | 11.43 | 2 | 3100 |  | 3 | 2 | 1 | 1 | 67 | 7.95 | 10.5 |  |  |  |  |  | 3 |
| 226 | 19.22 | 1 | 4200 |  | 3 | 3 | 1 | 2 | 82 | 10.465 | 12.3 |  |  |  |  |  | 2 |
| 227 | 10.35 | 1 | 3350 |  | 3 | 3 | 1 | 1 | 70 | 8.15 | 9.8 |  |  |  |  |  | 5 |
| 228 | 11.5 | 2 | 3300 |  | 4 | 4 | 1 | 1 | 74 | 9.555 | 10 |  |  |  |  |  | 2 |
| 229 | 20.76 | 2 | 3100 | 50 | 3 | 2 | 1 | 1 | 81 | 9.985 | 12.6 |  |  |  |  |  | 5 |
| 230 | 23.59 | 1 | 4100 |  | 3 | 2 | 1 | 1 | 80 | 9.945 | 9 |  |  |  |  |  | 5 |
| 231 | 30.55 | 1 | 2700 |  |  |  |  |  | 88 | 11.21 | 11.1 |  |  |  |  |  | 0 |
| 232 | 13.14 | 1 | 3800 |  | 3 | 3 | 1 | 1 | 77 | 9.77 | 10.7 |  |  |  |  |  | 4 |
| 233 | 19.65 | 1 | 3750 | 51 | 3 | 3 | 2 | 1 | 79 | 9.2 | 12.5 |  |  |  |  |  | 6 |
| 234 | 20.07 | 1 | 3750 |  | 3 | 2 | 1 | 1 | 81 | 10.5 | 13.3 |  |  |  |  |  | 4 |
| 235 | 22.11 | 2 | 2750 |  | 3 | 3 | 1 | 1 | 76 | 9.075 | 10.4 |  |  |  |  |  | 2 |
| 236 | 5.78 | 2 | 3400 | 51 | 4 | 3 | 1 | 1 | 70 | 7.595 | 12.8 |  |  |  |  |  | 2 |
| 237 | 18.6 | 1 | 3400 |  | 2 | 1 | 1 | 1 | 83 | 12.885 | 11.2 |  |  |  |  |  | 1 |
| 238 | 6.8 | 1 | 3600 |  | 3 | 2 | 1 | 1 | 63 | 5.615 | 8.3 |  |  |  |  |  | 1 |
| 239 | 10.09 | 2 | 3000 |  | 4 | 2 | 1 | 1 | 64 | 6.365 | 13.9 |  |  |  |  |  | 5 |
| 240 | 14.42 | 1 | 3600 |  | 2 | 2 | 1 | 1 | 76 | 9.995 | 14 |  |  |  |  |  | 4 |
| 241 | 10.05 | 2 | 2900 |  | 3 | 3 | 1 | 1 | 69 | 7.675 | 9.8 |  |  |  |  |  | 4 |
| 242 | 5.22 | 1 | 3350 | 50 | 3 | 3 | 1 | 2 | 64 | 7.395 | 11.5 |  |  |  |  |  | 0 |
| 243 | 17.97 | 1 | 3350 | 50 | 2 | 2 | 1 | 1 | 78 | 9.18 | 13.1 | 195.62 | 14.21 | 12.23 | 104.58 |  | 3 |
| 244 | 5.88 | 2 | 3000 | 50 | 3 | 3 | 2 | 2 | 65 | 7.975 | 10.7 |  |  |  |  |  | 0 |
| 245 | 20.86 | 1 | 3350 | 50 | 3 | 2 | 1 | 1 | 85 | 12.555 | 12.8 |  |  |  |  |  | 4 |
| 246 | 3.71 | 2 | 3150 | 52 | 3 | 2 | 2 | 1 | 60.5 | 5.935 | 11.7 |  |  |  |  |  | 0 |
| 247 | 12.16 | 2 | 3250 |  | 3 | 3 | 1 | 2 | 73 | 9.215 | 11.3 |  |  |  |  |  | 3 |
| 248 | 1.97 | 2 | 2500 |  | 3 | 3 | 1 | 1 | 54 | 4.148 | 9.2 |  |  |  |  |  | 0 |
| 249 | 14.49 | 1 | 2500 | 50 | 3 | 3 | 2 | 1 | 73 | 8.535 | 11.7 |  |  |  |  |  | 5 |
| 250 | 6.57 | 2 | 3000 | 50 | 5 | 2 | 2 | 2 | 62 | 6.585 | 9.6 |  |  |  |  |  | 0 |
| 251 | 2.76 | 1 | 2500 | 50 | 3 | 2 | 2 | 1 | 60 | 5.16 | 9.2 |  |  |  |  |  | 1 |
| 252 | 4.96 | 1 | 3000 |  | 4 | 2 | 1 | 1 | 64 | 8 | 13.7 |  |  |  |  |  | 1 |
| 253 | 5.06 | 1 | 3250 | 51 | 2 | 1 | 1 | 1 | 64 | 7.505 | 11.8 |  |  |  |  |  | 0 |
| 254 | 6.97 | 1 | 3000 |  | 2 | 2 | 1 | 1 | 63.5 | 7.13 | 10.8 |  |  |  |  |  | 3 |
| 255 | 15.34 | 2 | 2750 | 50 | 3 | 2 | 2 | 1 | 77 | 9.235 | 12 |  |  |  |  |  | 4 |
| 256 | 9 | 2 | 3600 | 53 | 2 | 1 | 1 | 1 | 72 | 9.625 | 11.8 |  |  |  |  |  | 2 |
| 257 | 20.04 | 1 | 3200 | 50 | 4 | 3 | 1 | 1 | 82 | 10.01 | 10.5 | 148.78 | 13.59 | 6.20 | 69.66 | 0.89 | 2 |
| 258 | 2.92 | 1 | 2400 | 45 | 3 | 3 | 1 | 1 | 58 | 5.685 | 11 |  |  |  |  |  | 0 |
| 259 | 2.92 | 1 | 1800 | 42 | 3 | 3 | 1 | 1 | 57 | 4.85 | 8.9 |  |  |  |  |  | 0 |
| 260 | 10.51 | 1 | 3600 |  | 2 | 3 | 1 | 1 | 75 | 9.915 | 10 |  |  |  |  |  | 1 |
| 261 | 8.9 | 1 | 2900 | 50 | 2 | 2 | 1 | 1 | 67 | 8.315 | 10.5 |  |  |  |  |  | 2 |
| 262 | 23.98 | 1 | 3800 |  | 3 | 2 | 2 | 1 | 82 | 11.09 | 8.9 | 324.15 | 16.92 |  |  |  | 4 |
| 263 | 15.31 | 2 | 2500 | 51 | 3 | 3 | 1 | 1 | 75.5 | 9.015 | 11.2 |  |  |  |  |  | 5 |
| 264 | 16.26 | 2 | 3750 | 50 | 3 | 3 | 1 | 1 | 77 | 8.515 | 10.7 |  |  |  |  |  | 4 |
| 265 | 10.48 | 1 | 3350 | 50 | 3 | 3 | 1 | 1 | 73.6 | 9.03 | 11 |  |  |  |  |  | 4 |
| 266 | 9.76 | 2 | 3000 |  | 3 | 3 | 2 | 1 | 72.5 | 9.32 | 11.6 |  |  |  |  |  | 5 |
| 267 | 21.19 | 2 | 3100 | 50 | 3 | 3 | 1 | 1 | 80 | 11.24 | 13.2 | 396.78 | 3.98 | 16.11 | 91.95 | 1.39 | 4 |
| 268 | 13.08 | 1 | 3650 |  | 2 | 2 | 1 | 1 | 74 | 9.64 | 9 |  |  |  |  |  | 3 |
| 269 | 10.28 | 1 | 3150 | 51 | 4 | 3 | 1 | 2 | 75 | 9.605 | 11.3 |  |  |  |  |  | 1 |
| 270 | 17.97 | 1 | 3300 | 50 | 3 | 2 | 2 | 2 | 76 | 8.7 | 11.2 |  |  |  |  |  | 1 |
| 271 | 17.68 | 1 | 3800 | 50 | 3 | 2 | 1 | 1 | 75.1 | 8.99 | 11.1 |  |  |  |  |  | 4 |
| 272 | 21.19 | 1 | 3150 | 50 | 3 | 3 | 1 | 1 | 71.5 | 8.92 | 11.4 |  |  |  |  |  | 4 |
| 273 | 7.79 | 2 | 3500 |  | 3 | 3 | 1 | 1 | 64 | 6.98 | 10.8 |  |  |  |  |  | 6 |
| 274 | 16.62 | 2 |  |  | 3 | 3 | 2 | 1 | 77 | 9.495 | 12 |  |  |  |  |  | 3 |
| 275 | 3.81 | 1 |  |  | 3 | 3 | 1 | 1 | 58.5 | 6.71 | 10.5 |  |  |  |  |  | 0 |
| 276 | 14.09 | 1 | 3500 | 50 | 2 | 1 | 1 | 1 | 75 | 8.425 | 10.6 |  |  |  |  |  | 2 |
| 277 | 4.24 | 2 | 3000 | 50 | 3 | 3 | 1 | 1 | 62 | 6.25 | 10.3 |  |  |  |  |  | 0 |
| 278 | 18.5 | 1 | 2300 | 48 | 3 | 3 | 1 | 1 | 78 | 9.218 | 12.9 |  |  |  |  |  | 1 |
| 279 | 18.43 | 2 | 3250 | 50 | 3 | 3 | 1 | 1 | 75 | 8.785 | 11.7 |  |  |  |  |  | 5 |
| 280 | 25 | 2 | 2750 | 50 | 3 | 3 | 1 | 1 | 82.5 | 9.56 | 13.1 | 326.42 | 15.20 | 19.14 | 89.18 |  | 4 |
| 281 | 21.72 | 2 | 3000 |  | 3 | 2 | 1 | 1 | 77 | 9.125 | 11.4 |  |  |  |  |  | 5 |
| 282 | 20.04 | 1 |  |  | 2 | 1 | 1 | 1 | 80 | 9.985 | 11.5 |  |  |  |  |  | 4 |
| 283 | 26.45 | 1 |  |  | 3 | 3 | 1 | 1 | 85.5 | 12.6 | 13.1 | 382.48 | 7.42 | 18.01 | 52.67 | 1.00 | 3 |
| 284 | 6.28 | 2 | 3000 | 50 | 3 | 3 | 2 | 1 | 65 | 7.35 | 9.6 |  |  |  |  |  | 1 |
| 285 | 27.01 | 1 | 3550 |  | 4 | 3 | 1 | 1 | 83 | 11.15 | 12.6 | 261.59 | 4.36 | 26.24 | 75.06 | 1.05 | 5 |
| 286 | 26.32 | 2 | 3250 |  | 3 | 3 | 1 | 1 | 84 | 11.17 | 12.1 | 261.59 | 5.26 | 16.11 | 57.73 | 0.79 | 5 |
| 287 | 14.26 | 2 | 3000 | 50 | 4 | 3 | 1 | 1 | 74 | 7.85 | 11.1 |  |  |  |  |  | 4 |
| 288 | 15.7 | 2 | 3700 | 48 | 3 | 3 | 1 | 1 | 80.5 | 9.225 | 11 |  |  |  |  |  | 3 |
| 289 | 2.4 | 1 | 3800 |  | 3 | 2 | 1 | 1 | 59 | 6.045 | 10.9 |  |  |  |  |  | 0 |
| 290 | 12.22 | 2 | 3250 |  | 5 | 4 | 1 | 1 | 73 | 8.245 | 13 |  |  |  |  |  | 5 |
| 291 | 7.39 | 2 | 3500 |  | 3 | 2 | 1 | 1 | 65 | 7.065 | 11.6 |  |  |  |  |  | 3 |
| 292 | 15.64 | 2 |  |  | 2 | 2 | 1 | 1 | 66.2 | 6.13 | 9.1 |  |  |  |  |  | 2 |
| 293 | 8.05 | 1 | 3000 |  | 4 | 1 | 1 | 1 | 63 | 7.505 | 12 |  |  |  |  |  | 0 |
| 294 | 29.6 | 2 | 2800 | 50 | 3 | 3 | 1 | 1 | 83.5 | 10.29 | 10.1 |  |  |  |  |  | 2 |
| 295 | 21.19 | 1 | 2750 |  | 2 | 2 | 1 | 1 | 80 | 9.49 | 12.4 |  |  |  |  |  | 4 |
| 296 | 11.1 | 1 | 3600 |  | 3 | 3 | 1 | 1 | 84 | 13.31 | 10.4 |  |  |  |  |  | 4 |
| 297 | 9.46 | 1 | 3000 |  | 2 | 3 | 1 | 1 | 67 | 7.3 | 10.5 |  |  |  |  |  | 2 |
| 298 | 19.58 | 1 | 2250 | 42 | 2 | 3 | 1 | 1 | 82 | 10.17 | 10.9 |  |  |  |  |  | 5 |
| 299 | 22.97 | 2 | 2750 | 50 | 4 | 3 | 1 | 1 | 79 | 9.255 | 11.3 |  |  |  |  |  | 4 |
| 300 | 32.3 | 1 | 3000 | 49 | 3 | 2 | 1 | 1 | 87.7 | 11.81 | 11.6 | 219.41 | 14.47 | 7.33 | 73.45 | 1.01 | 3 |
| 301 | 24.21 | 1 | 3700 | 50 | 2 | 2 | 1 | 1 | 81 | 9.828 | 11.8 |  |  |  |  |  | 2 |
| 302 | 5.22 | 1 |  |  | 3 | 2 | 1 | 1 | 63 | 7.78 | 10.5 |  |  |  |  |  | 0 |
| 303 | 13.37 | 1 |  |  | 3 | 1 | 1 | 1 | 74 | 8.52 | 10.7 |  |  |  |  |  | 1 |
| 304 | 15.54 | 2 | 3900 | 49 | 3 | 3 | 1 | 1 | 70 | 8.41 | 11.6 |  |  |  |  |  | 4 |
| 305 | 32.92 | 2 | 3300 |  | 4 | 3 | 1 | 1 | 92.1 | 14.6 | 11.1 | 195.62 | 2.54 | 16.15 | 52.67 | 1.04 | 2 |
| 306 | 18.69 | 1 | 3000 |  | 3 | 3 | 2 | 1 | 79.5 | 9.27 | 11.9 |  |  |  |  |  | 4 |
| 307 | 5.91 | 1 | 3400 |  | 6 | 4 | 1 | 2 | 71.3 | 8.205 | 11.2 |  |  |  |  |  | 2 |
| 308 | 5.65 | 1 | 4100 |  | 4 | 4 | 1 | 1 | 65 | 7.96 | 11.5 |  |  |  |  |  | 2 |
| 309 | 25.2 | 2 |  |  | 5 | 3 | 2 | 1 | 85 | 10.63 | 13.2 | 280.49 | 9.50 | 14.87 | 62.64 |  | 4 |
| 310 | 26.15 | 1 |  |  | 3 | 3 | 1 | 1 | 81 | 9.64 | 12.4 | 218.40 | 7.76 | 21.52 | 61.59 | 0.84 | 5 |
| 311 | 22.87 | 1 | 2550 | 47 | 3 | 2 | 1 | 1 | 84 | 10.86 | 11.5 |  |  |  |  |  | 3 |
| 312 | 19.12 | 2 | 3450 |  | 4 | 2 | 1 | 1 | 70.5 | 8.18 | 10.1 |  |  |  |  |  | 4 |
| 313 | 8.11 | 1 | 3000 | 48 | 3 | 2 | 2 | 1 | 71 | 9.65 | 12.4 |  |  |  |  |  | 2 |
| 314 | 26.74 | 1 | 4100 |  | 3 | 2 | 1 | 1 | 93 | 15.16 | 12.7 | 306.00 | 9.44 | 35.80 | 42.50 | 1.13 | 5 |
| 315 | 24.11 | 2 | 3500 |  | 3 | 3 | 1 | 1 | 83 | 11.93 | 9 | 312.98 | 9.35 | 13.30 | 44.77 | 0.93 | 3 |
| 316 | 35.61 | 1 | 2700 |  | 4 | 3 | 1 | 1 | 93.2 | 11.24 | 7.7 |  |  |  |  |  | 2 |
| 317 | 13.73 | 1 | 3500 |  | 5 | 3 | 2 | 1 | 73.3 | 8.355 | 11.4 |  |  |  |  |  | 2 |
| 318 | 22.8 | 1 | 3400 | 55 | 3 | 4 | 1 | 1 | 81.5 | 10.816 | 14.5 |  | 14.71 | 20.36 | 58.62 | 0.75 | 5 |
| 319 | 28.35 | 1 | 2500 |  | 5 | 3 | 1 | 1 | 85.8 | 10.57 | 8.2 | 401.11 | 3.79 | 1.08 | 85.82 | 0.88 | 3 |
| 320 | 14.88 | 1 | 3250 |  | 4 | 4 | 2 | 1 | 79 | 11.345 | 13 |  | 16.85 | 20.88 | 63.28 | 1.13 | 5 |
| 321 | 1.48 | 2 | 3000 |  | 4 | 3 | 1 | 1 | 56.7 | 4.285 | 11.7 |  |  |  |  |  | 0 |
| 322 | 10.97 | 1 | 3100 |  | 3 | 3 | 1 | 2 | 70.5 | 8.255 | 9.3 |  |  |  |  |  | 2 |
| 323 | 33.38 | 2 |  |  | 3 | 2 | 2 | 1 | 85.7 | 11.335 | 12.3 | 300.41 | 8.12 | 23.39 | 47.20 | 1.06 | 2 |
| 324 | 3.52 | 2 | 2900 | 50 | 4 | 3 | 1 | 1 | 63.5 | 5.805 | 12 |  |  |  |  |  | 0 |
| 325 | 21.91 | 2 | 3000 |  | 3 | 3 | 1 | 1 | 83.6 | 11.265 | 12.3 |  |  |  |  |  | 4 |
| 326 | 13.8 | 2 | 4000 |  | 3 | 3 | 2 | 1 | 76 | 10.425 | 11.2 | 184.53 | 15.12 | 8.89 | 58.62 | 1.08 | 4 |
| 327 | 10.18 | 1 | 4000 | 50 | 3 | 1 | 1 | 1 | 72.2 | 8.335 | 10.3 |  |  |  |  |  | 0 |
| 328 | 30.82 | 2 | 3750 | 52 | 3 | 1 | 1 | 2 | 85.5 | 12.595 | 11.9 | 326.88 | 9.46 | 37.89 | 56.21 | 0.88 | 4 |
| 329 | 34.69 | 1 | 2900 |  | 5 | 5 | 1 | 1 | 86.8 | 11.125 | 12 | 472.68 | 6.10 | 28.43 | 77.70 | 1.43 | 4 |
| 330 | 10.71 | 2 | 2650 | 48 | 2 | 3 | 1 | 1 | 68 | 7.37 | 10.5 |  |  |  |  |  | 2 |
| 331 | 6.37 | 2 | 3250 | 52 | 3 | 3 | 2 | 2 | 71 | 7.375 | 11 |  |  |  |  |  | 5 |
| 332 | 27.33 | 1 | 4250 |  | 3 | 6 | 2 | 1 | 88.5 | 13.045 | 13.2 | 408.23 | 6.78 | 14.19 | 62.22 | 1.56 | 5 |
| 333 | 7.59 | 1 |  |  | 3 | 2 | 1 | 2 | 78.8 | 10.105 | 12.3 | 509.42 | 8.18 | 19.60 | 51.19 |  | 6 |
| 334 | 34.99 | 1 | 3600 |  | 3 | 3 | 1 | 2 | 95.4 | 15.17 | 9.7 | 237.87 | 11.77 | 72.70 | 55.51 | 0.98 | 4 |
| 335 | 26.84 | 1 | 3750 |  | 3 | 2 | 1 | 1 | 82.1 | 11.29 | 11.7 |  |  |  |  |  | 3 |
| 336 | 34.04 | 1 | 3500 |  | 2 | 2 | 1 | 1 | 87 | 12.24 | 11.9 |  |  |  |  |  | 3 |
| 337 | 30.32 | 2 | 2600 |  | 4 | 4 | 1 | 1 | 92 | 12.785 | 12.9 | 521.82 | 6.47 | 7.31 | 62.85 | 1.13 | 5 |
| 338 | 10.81 | 2 | 3600 |  | 3 | 3 | 1 | 1 | 70 | 8.145 | 10.7 |  |  |  |  |  | 2 |
| 339 | 15.93 | 2 | 3100 |  | 3 | 3 | 2 | 1 | 75.5 | 8.105 | 12 |  |  |  |  |  | 4 |
| 340 | 5.49 | 1 | 3750 | 50 | 3 | 4 | 2 | 1 | 68 | 7.715 | 11.9 |  |  |  |  |  | 1 |
| 341 | 29.04 | 1 | 3600 | 50 | 3 | 4 | 1 | 1 | 85.3 | 11.5 | 11.1 | 345.55 | 6.46 | 49.53 | 75.08 | 1.03 | 3 |
| 342 | 17.84 | 1 | 3100 |  | 3 | 3 | 1 | 1 | 80 | 8.865 | 12.5 | 288.25 | 12.98 | 6.99 | 57.49 | 1.00 | 4 |
| 343 | 6.77 | 2 | 2600 |  | 4 | 3 | 2 | 2 | 63 | 6.55 | 12.5 |  |  |  |  |  | 4 |
| 344 | 8.34 | 1 | 3700 | 51 | 3 | 3 | 1 | 2 | 72 | 9.715 | 11.5 |  |  |  |  |  | 3 |
| 345 | 24.25 | 1 | 3800 | 52 | 2 | 2 | 2 | 2 | 84 | 10.495 | 12.2 |  |  |  |  |  | 3 |
| 346 | 15.64 | 1 | 3000 |  | 2 | 2 | 1 | 2 | 76 | 9.418 | 11.8 | 224.53 | 9.77 | 21.26 | 70.27 | 0.82 | 6 |
| 347 | 28.45 | 1 |  |  | 2 | 3 | 1 | 1 | 86 | 12.95 | 12.7 |  |  |  |  |  | 2 |
| 348 | 1.74 | 2 | 2650 |  | 3 | 3 | 1 | 1 | 56.7 | 3.718 | 11.7 |  |  |  |  |  | 0 |
| 349 | 18.33 | 1 | 3500 | 53 | 3 | 2 | 1 | 1 | 89.2 | 14.25 | 13.4 |  |  |  |  |  | 5 |
| 350 | 2.2 | 1 | 2900 | 50 | 3 | 4 | 1 | 1 | 59 | 5.185 | 11.1 |  |  |  |  |  | 0 |
| 351 | 10.41 | 1 | 3100 |  | 3 | 3 | 1 | 1 | 67 | 7.37 | 10.3 |  |  |  |  |  | 4 |
| 352 | 19.81 | 2 |  |  | 3 | 3 | 1 | 1 | 77.5 | 9.345 | 11.6 | 151.45 | 9.54 | 22.18 | 44.63 |  | 4 |
| 353 | 26.22 | 1 | 3500 |  | 4 | 3 | 1 | 1 | 85.1 | 11.585 | 12.2 | 598.81 | 7.48 | 18.91 | 64.58 | 1.10 | 4 |
| 354 | 18.14 | 2 | 3550 |  | 3 | 2 | 1 | 1 | 74.3 | 9.078 | 10.5 | 160.75 | 15.60 | 11.06 | 69.77 | 0.85 | 4 |
| 355 | 11.96 | 1 | 4000 |  | 3 | 2 | 1 | 1 | 72.5 | 8.75 | 9.7 |  |  |  |  |  | 5 |
| 356 | 9.36 | 2 | 3250 |  | 3 | 3 | 2 | 1 | 72.2 | 7.776 | 10.1 |  |  |  |  |  | 1 |
| 357 | 10.58 | 2 | 2700 |  | 3 | 2 | 1 | 1 | 67 | 7.755 | 11 |  |  |  |  |  | 4 |
| 358 | 33.41 | 2 | 3150 |  |  |  |  |  | 88 | 12.205 | 11.2 | 369.14 | 12.08 | 12.30 | 41.66 | 0.95 | 0 |
| 359 | 18.86 | 1 | 3000 |  | 3 | 2 | 1 | 1 | 77 | 9.21 | 9.8 |  |  |  |  |  | 6 |
| 360 | 20.21 | 2 |  |  | 2 | 2 | 1 | 1 | 75 | 8.94 | 11.6 |  |  |  |  |  | 6 |
| 361 | 16.1 | 1 | 4000 |  | 3 | 3 | 1 | 1 | 80.5 | 10.22 | 13.1 |  |  |  |  |  | 3 |
| 362 | 15.28 | 1 | 3500 |  | 4 | 2 | 1 | 1 | 76.5 | 9.65 | 12.8 | 344.12 | 3.56 | 12.43 | 60.58 | 0.84 | 4 |
| 363 | 28.52 | 1 | 3000 |  | 2 | 1 | 1 | 1 | 75.1 | 8 | 10.9 |  |  |  |  |  | 2 |
| 364 | 8.44 | 2 |  |  | 6 | 4 | 1 | 1 | 79 | 9.735 | 11.1 |  |  |  |  |  | 5 |
| 365 | 22.08 | 1 |  |  | 3 | 2 | 1 | 1 | 77.7 | 9.18 | 11.3 | 254.75 | 13.07 | 21.08 | 35.14 | 0.86 | 4 |
| 366 | 10.61 | 1 | 3700 | 53 | 4 | 3 | 1 | 1 | 72 | 8.48 | 10.4 |  |  |  |  |  | 1 |
| 367 | 24.97 | 1 | 3200 |  | 2 | 1 | 1 | 1 | 88 | 11.37 | 11.1 | 159.91 | 10.28 | 28.70 | 60.58 | 0.78 | 5 |
| 368 | 18.6 | 1 | 3500 |  | 3 | 3 | 2 | 1 | 81.8 | 9.86 | 12.2 |  |  |  |  |  | 4 |
| 369 | 13.9 | 1 |  |  | 3 | 3 | 1 | 1 | 85 | 11.26 | 11.5 | 120.82 | 10.10 | 8.97 | 54.12 | 0.97 | 4 |
| 370 | 10.25 | 1 | 3350 |  | 3 | 2 | 1 | 1 | 70 | 8.575 | 10.1 |  |  |  |  |  | 3 |
| 371 | 34.63 | 1 | 3600 |  |  |  |  |  | 88.5 | 12.865 | 12.2 |  |  |  |  |  | 0 |
| 372 | 22.44 | 2 |  |  | 3 | 2 | 2 | 1 | 79 | 9.689 | 11.1 |  |  |  |  |  | 5 |
| 373 | 12.45 | 2 |  |  | 2 | 1 | 2 | 1 | 69 | 7.332 | 9.9 |  |  |  |  |  | 3 |
| 374 | 26.12 | 2 |  |  | 3 | 2 | 1 | 1 | 82 | 9.97 | 11.1 | 192.81 | 6.89 | 19.15 | 55.51 | 1.01 | 4 |
| 375 | 15.8 | 1 | 4000 |  | 2 | 3 | 1 | 2 | 74.4 | 8.36 | 12.5 |  |  |  |  |  | 4 |
| 376 | 16.79 | 2 | 2200 |  | 3 | 3 | 1 | 1 | 76 | 8.71 | 12.7 |  | 8.74 | 37.81 | 56.21 |  | 3 |
| 377 | 8.21 | 1 |  |  | 3 | 3 | 1 | 1 | 78 | 10.595 | 9.4 |  |  |  |  |  | 3 |
| 378 | 34.92 | 1 |  |  |  |  |  |  | 89.3 | 12.621 | 10.4 |  |  |  |  |  | 0 |
| 379 | 32.43 | 2 |  |  |  |  |  |  | 84 | 11.023 | 13.6 |  |  |  |  |  | 0 |
| 380 | 12.35 | 2 |  |  |  |  |  |  | 70.5 | 8.065 | 11.5 |  |  |  |  |  | 0 |
| 381 | 19.68 | 2 |  |  |  |  |  |  | 76 | 8.365 | 11.3 |  |  |  |  |  | 0 |
| 382 | 20.24 | 2 | 3100 | 50 |  |  |  |  | 82 | 10.39 | 9.3 |  |  |  |  |  | 0 |
| 383 | 19.81 | 1 | 3300 |  | 4 | 4 | 1 | 1 | 75 | 8.745 | 9.6 |  |  |  |  |  | 5 |
| 384 | 22.28 | 1 | 3450 |  | 3 | 2 | 1 | 1 | 85 | 12.355 | 11.8 | 287.84 | 2.83 | 9.10 | 89.60 | 0.98 | 4 |
| 385 | 2.92 | 1 | 3350 |  | 3 | 1 | 1 | 1 | 59 | 5.505 | 8.7 |  |  |  |  |  | 1 |
| 386 | 20.93 | 2 | 4000 |  | 4 | 4 | 1 | 1 | 76 | 9.355 | 12.7 |  |  |  |  |  | 7 |
| 387 | 5.98 | 2 | 3000 |  | 3 | 3 | 2 | 1 | 64 | 6.785 | 11.5 |  |  |  |  |  | 3 |
| 388 | 6.37 | 1 | 3300 | 50 | 3 | 3 | 1 | 2 | 67 | 7.505 | 9.4 |  |  |  |  |  | 1 |
| 389 | 17.15 | 1 | 3750 | 50 | 3 | 4 | 2 | 1 | 80 | 10.395 | 10.4 |  |  |  |  |  | 4 |
| 390 | 9.46 | 2 | 3000 |  | 3 | 3 | 1 | 1 | 71 | 9.105 | 11.5 |  |  |  |  |  | 2 |
| 391 | 17.45 | 2 | 1500 |  | 3 | 3 | 1 | 2 | 73 | 7.205 | 12.7 |  |  |  |  |  | 2 |
| 392 | 19.61 | 2 | 2700 |  | 3 | 3 | 1 | 1 | 76 | 8.575 | 12.2 |  |  |  |  |  | 3 |
| 393 | 3.06 | 2 | 2600 | 50 | 3 | 3 | 2 | 1 | 60 | 5.485 | 10.9 |  |  |  |  |  | 0 |
| 394 | 5.06 | 1 | 2250 |  | 3 | 2 | 1 | 1 | 61 | 6.24 | 10 |  |  |  |  |  | 3 |
| 395 | 13.54 | 1 | 2600 | 48 | 3 | 3 | 1 | 1 | 69 | 6.84 | 12 |  |  |  |  |  | 4 |
| 396 | 8.48 | 1 | 3600 | 53 | 3 | 3 | 2 | 1 | 70 | 8.64 | 11.9 |  |  |  |  |  | 2 |
| 397 | 2.5 | 2 | 3650 |  | 3 | 3 | 1 | 1 | 60 | 5.68 | 13.8 |  |  |  |  |  | 0 |
| 398 | 24.48 | 2 | 3400 |  |  |  |  |  | 80 | 9.46 | 13.9 | 505.36 | 7.72 | 39.01 | 243.53 | 1.58 | 0 |
| 399 | 15.31 | 1 |  |  | 2 | 2 | 1 | 1 | 77 | 9.55 | 14 |  |  |  |  |  | 5 |
| 400 | 22.64 | 2 | 2900 |  | 4 | 3 | 2 | 1 | 84 | 12.84 | 13.1 |  |  |  |  |  | 3 |
| 401 | 3.52 | 1 | 3800 |  | 3 | 3 | 1 | 2 | 65 | 7.905 | 10.9 |  |  |  |  |  | 0 |
| 402 | 1.54 | 1 | 2700 |  | 4 | 3 | 1 | 1 | 53 | 4.395 | 11.2 |  |  |  |  |  | 0 |
| 403 | 29.27 | 1 | 4200 |  |  |  |  |  | 91 | 12.495 | 13.6 | 215.38 | 8.67 | 13.34 | 92.90 | 0.93 | 0 |
| 404 | 1.97 | 1 | 3400 | 50 | 3 | 3 | 1 | 1 | 61 | 6.045 | 13.4 |  |  |  |  |  | 0 |
| 405 | 5.95 | 1 | 3000 |  | 3 | 3 | 2 | 1 | 64 | 7.315 | 11.3 |  |  |  |  |  | 1 |
| 406 | 17.77 | 1 | 4000 |  | 3 | 3 | 1 | 1 | 83 | 10.24 | 9.5 |  |  |  |  |  | 3 |
| 407 | 12.52 | 2 | 3300 | 49 | 3 | 4 | 2 | 1 | 71.5 | 8.225 | 12 |  |  |  |  |  | 2 |
| 408 | 20.86 | 2 | 3000 |  | 4 | 3 | 1 | 1 | 85 | 11.05 | 10.6 |  |  |  |  |  | 2 |
| 409 | 2.73 | 2 | 3100 |  | 3 | 3 | 1 | 1 | 60 | 5.57 | 8.3 |  |  |  |  |  | 2 |
| 410 | 22.7 | 2 | 2900 |  | 3 | 3 | 1 | 1 | 80 | 9.68 | 11.4 | 282.51 | 5.94 | 6.99 | 72.81 | 1.08 | 5 |
| 411 | 11.76 | 1 | 3900 |  | 4 | 3 | 2 | 1 | 67 | 7.975 | 9.9 |  |  |  |  |  | 2 |
| 412 | 13.11 | 1 | 3000 |  | 3 | 3 | 1 | 1 | 72 | 8.95 | 12.3 |  |  |  |  |  | 2 |
| 413 | 6.18 | 1 | 4000 | 52 | 3 | 2 | 1 | 1 | 70 | 8.565 | 11.3 |  |  |  |  |  | 1 |
| 414 | 18.79 | 2 | 3000 |  | 3 | 3 | 1 | 1 | 75 | 7.335 | 10.2 |  |  |  |  |  | 3 |
| 415 | 3.42 | 2 | 4500 |  | 3 | 4 | 1 | 1 | 61 | 6.235 | 10 |  |  |  |  |  | 1 |
| 416 | 29.08 | 1 | 3000 |  |  |  |  |  | 92 | 13.48 | 12.9 | 298.71 | 7.86 | 9.72 | 81.65 | 0.97 | 0 |
| 417 | 8.41 | 1 | 2750 |  | 2 | 3 | 2 | 1 | 68 | 8.115 | 11.1 |  |  |  |  |  | 1 |
| 418 | 8.34 | 1 | 3150 |  | 3 | 3 | 1 | 2 | 70 | 9.06 | 8.9 |  |  |  |  |  | 4 |
| 419 | 9.23 | 2 | 2900 |  | 1 | 1 | 1 | 2 | 66 | 6.48 | 10.7 |  |  |  |  |  | 2 |
| 420 | 2 | 1 | 3000 |  | 3 | 4 | 1 | 1 | 59 | 5.69 | 10.5 |  |  |  |  |  | 0 |
| 421 | 3.84 | 1 | 2500 |  | 3 | 3 | 1 | 1 | 59 | 6.42 | 10.4 |  |  |  |  |  | 0 |
| 422 | 10.05 | 1 |  |  | 3 | 3 | 1 | 1 | 75 | 9.37 | 12.9 |  |  |  |  |  | 3 |
| 423 | 7.56 | 2 | 2700 | 54 | 4 | 4 | 1 | 1 | 66 | 7.735 | 12.4 |  |  |  |  |  | 5 |
| 424 | 9.86 | 1 | 3550 | 50 | 4 | 3 | 1 | 1 | 71 | 9.135 | 9.6 |  |  |  |  |  | 2 |
| 425 | 5.26 | 1 | 2700 | 50 | 3 | 3 | 1 | 1 | 65 | 7.57 | 11.7 |  |  |  |  |  | 0 |
| 426 | 8.21 | 2 | 4100 |  | 3 | 3 | 1 | 1 | 66 | 8.65 | 11.4 |  |  |  |  |  | 4 |
| 427 | 2.23 | 1 | 3700 |  | 5 | 5 | 1 | 1 | 62 | 6.45 | 9.2 |  |  |  |  |  | 0 |
| 428 | 20.83 | 2 | 4400 |  |  |  |  |  | 80 | 9.995 | 11.6 |  |  |  |  |  | 0 |
| 429 | 5.16 | 2 | 3150 | 48 | 3 | 2 | 1 | 2 | 64.5 | 7.43 | 11.9 |  |  |  |  |  | 2 |
| 430 | 30.95 | 1 | 3500 |  | 2 | 3 | 1 | 2 | 90 | 13.05 | 12.4 | 138.09 | 5.30 | 11.78 | 71.29 | 1.03 | 3 |
| 431 | 13.37 | 1 | 3700 |  | 2 | 3 | 1 | 2 | 74.5 | 8.28 | 11.8 |  |  |  |  |  | 2 |
| 432 | 19.75 | 2 | 2250 |  | 3 | 3 | 1 | 1 | 76 | 9.64 | 11.4 |  |  |  |  |  | 5 |
| 433 | 10.74 | 2 |  |  | 2 | 2 | 2 | 1 | 68 | 7.805 | 11.1 |  |  |  |  |  | 3 |
| 434 | 14.36 | 2 | 3000 | 50 | 6 | 3 | 1 | 1 | 76 | 9.51 | 12.3 |  |  |  |  |  | 5 |
| 435 | 16.33 | 2 | 3200 | 50 | 6 | 5 | 1 | 1 | 80 | 9.99 | 11.4 | 596.41 | 12.35 | 14.73 | 102.74 | 1.31 | 4 |
| 436 | 14.85 | 2 |  |  | 4 | 3 | 1 | 1 | 77.5 | 9.515 | 11.8 |  |  |  |  |  | 6 |
| 437 | 3.29 | 1 | 3600 |  | 4 | 1 | 1 | 1 | 61 | 7.3 | 10.5 |  |  |  |  |  | 0 |
| 438 | 14.46 | 2 |  |  | 2 | 2 | 1 | 1 | 73 | 8.755 | 13.5 |  |  |  |  |  | 5 |
| 439 | 21.36 | 2 | 3000 |  | 3 | 3 | 1 | 1 | 79 | 10.18 | 12.3 | 118.93 | 13.75 | 4.66 | 66.36 | 0.84 | 3 |
| 440 | 18.92 | 2 | 3000 |  | 3 | 3 | 1 | 1 | 81 | 9.205 | 12.1 | 444.41 | 12.19 | 30.91 | 83.06 | 1.01 | 4 |
| 441 | 10.22 | 2 | 2900 |  | 2 | 2 | 1 | 1 | 71 | 7.92 | 8.2 |  |  |  |  |  | 2 |
| 442 | 2.63 | 1 | 3000 |  | 3 | 3 | 1 | 1 | 60 | 6.405 | 9.3 |  |  |  |  |  | 0 |
| 443 | 20.7 | 1 | 3000 | 50 | 2 | 3 | 1 | 1 | 81 | 10.455 | 14.2 |  |  |  |  |  | 4 |
| 444 | 11.63 | 2 | 3000 | 50 | 2 | 2 | 1 | 1 | 71 | 8.715 | 11.4 |  |  |  |  |  | 4 |
| 445 | 13.5 | 1 |  |  | 2 | 1 | 2 | 2 | 70 | 7.55 | 9.9 |  |  |  |  |  | 3 |
| 446 | 1.61 | 1 | 3400 |  | 4 | 2 | 1 | 1 | 57 | 4.97 | 9.2 |  |  |  |  |  | 0 |
| 447 | 14.09 | 2 | 2950 |  | 4 | 4 | 1 | 1 | 74 | 8.725 | 11.4 |  |  |  |  |  | 4 |
| 448 | 19.15 | 1 | 3250 |  | 4 | 3 | 1 | 2 | 76 | 8.935 | 10.6 | 156.33 | 18.09 | 6.37 | 73.58 | 1.02 | 4 |
| 449 | 23.59 | 1 | 3400 |  |  |  |  |  | 84 | 10.77 | 9.5 |  |  |  |  |  | 0 |
| 450 | 1.94 | 2 | 3150 |  | 2 | 2 | 1 | 1 | 56 | 4.765 | 9.8 |  |  |  |  |  | 0 |
| 451 | 7.06 | 2 |  |  | 4 | 1 | 1 | 1 | 65 | 8.575 | 9.1 |  |  |  |  |  | 3 |
| 452 | 10.12 | 1 | 3150 |  |  |  |  |  | 69 | 8.45 | 12.4 |  |  |  |  |  | 0 |
| 453 | 2.3 | 2 | 2300 |  | 3 | 3 | 1 | 1 | 58 | 4.74 | 10.1 |  |  |  |  |  | 0 |
| 454 | 7.72 | 1 |  |  | 2 | 2 | 1 | 2 | 66 | 7.51 | 12.3 |  |  |  |  |  | 1 |
| 455 | 4.73 | 1 |  |  | 2 | 3 | 1 | 1 | 68 | 7.84 | 11 |  |  |  |  |  | 1 |
| 456 | 21.52 | 2 | 3200 |  |  |  |  |  | 81.2 | 9.38 | 10.6 |  |  |  |  |  | 0 |
| 457 | 22.14 | 1 | 3150 |  |  |  |  |  | 79 | 9.25 | 10.8 |  |  |  |  |  | 0 |
| 458 | 14.85 | 1 | 3000 | 45 |  |  |  |  | 73 | 8.975 | 10.3 |  |  |  |  |  | 0 |
| 459 | 9.63 | 1 | 3300 |  |  |  |  |  | 71 | 8.99 | 11.2 |  |  |  |  |  | 0 |
| 460 | 8.61 | 1 | 2950 |  |  |  |  |  | 80 | 9.295 | 12.6 |  |  |  |  |  | 0 |
| 461 | 24.67 | 1 | 3400 |  |  |  |  |  | 83 | 11.615 | 11.6 |  |  |  |  |  | 0 |
| 462 | 27.86 | 2 | 3250 |  |  |  |  |  | 80 | 9.88 | 11.2 |  |  |  |  |  | 0 |
| 463 | 9.49 | 2 | 3000 |  |  |  |  |  | 76 | 8.918 | 12.1 |  |  |  |  |  | 0 |
| 464 | 15.01 | 1 | 2500 |  |  |  |  |  | 74 | 9.605 | 11.2 |  |  |  |  |  | 0 |
| 465 | 14.36 | 1 | 3000 |  |  |  |  |  | 70 | 7.845 | 10.9 |  |  |  |  |  | 0 |
| 466 | 10.71 | 1 | 2500 |  |  |  |  |  | 66 | 7.435 | 11.5 |  |  |  |  |  | 0 |
| 467 | 14.95 | 1 | 3100 |  |  |  |  |  | 75 | 9.94 | 8.8 |  |  |  |  |  | 0 |
| 468 | 15.74 | 2 | 3250 |  |  |  |  |  | 74 | 9.745 | 9.8 |  |  |  |  |  | 0 |
| 469 | 24.05 | 1 | 3100 | 49 |  |  |  |  | 79 | 9.255 | 12.5 |  |  |  |  |  | 0 |
| 470 | 15.9 | 1 | 4300 |  |  |  |  |  | 74 | 9.335 | 12.5 |  |  |  |  |  | 0 |
| 471 | 4.76 | 1 | 3250 |  |  |  |  |  | 70 | 7.42 | 10.4 |  |  |  |  |  | 0 |
| 472 | 11.7 | 2 | 2500 |  |  |  |  |  | 73 | 8.655 | 10.8 |  |  |  |  |  | 0 |
| 473 | 20.76 | 2 |  |  |  |  |  |  | 75 | 8.12 | 11.4 |  |  |  |  |  | 0 |
| 474 | 2.43 | 1 |  |  |  |  |  |  | 62 | 6.075 | 10.3 |  |  |  |  |  | 0 |
| 475 | 4.57 | 2 | 3850 | 50 |  |  |  |  | 59 | 6.05 | 9.2 |  |  |  |  |  | 0 |
| 476 | 1.58 | 1 | 3000 | 50 |  |  |  |  | 57 | 4.98 | 11.6 |  |  |  |  |  | 0 |
| 477 | 7.13 | 1 | 3800 |  |  |  |  |  | 70 | 7.915 | 10.2 |  |  |  |  |  | 0 |
| 478 | 8.94 | 2 |  |  |  |  |  |  | 64 | 6.185 | 11.8 |  |  |  |  |  | 0 |
| 479 | 9.4 | 2 | 4250 |  |  |  |  |  | 78 | 9.66 | 10.3 |  |  |  |  |  | 0 |
| 480 | 13.6 | 2 | 2800 | 50 | 3 | 3 | 1 | 1 | 75 | 8.3 | 8.3 |  |  |  |  |  | 1 |
| 481 | 21.75 | 1 | 3000 |  | 3 | 3 | 2 | 1 | 79.5 | 11.3 | 10.5 |  |  |  |  |  | 3 |
| 482 | 21.75 | 1 | 3000 |  | 3 | 3 | 2 | 1 | 79.5 | 9.7 | 13.2 |  |  |  |  |  | 3 |
| 483 | 33.12 | 1 | 3300 |  | 3 | 2 | 2 | 1 | 88 | 11.85 | 10.2 |  |  |  |  |  | 6 |
| 484 | 13.83 | 2 | 3000 |  | 2 | 2 | 2 | 1 | 75 | 8.3 | 12.7 |  |  |  |  |  | 3 |
| 485 | 31.8 | 1 | 2500 | 49 | 3 | 3 | 1 | 1 | 87.5 | 11.2 | 10.7 |  |  |  |  |  | 5 |
| 486 | 21.06 | 2 | 3100 |  | 3 | 3 | 1 | 1 | 78 | 7.8 | 12.3 | 231.48 | 17.93 | 16.09 | 63.55 | 1.12 | 2 |
| 487 | 21.26 | 2 |  |  | 3 | 1 | 1 | 2 | 82 | 9.7 | 11.5 |  |  |  |  |  | 3 |
| 488 | 11.56 | 1 | 3600 | 55 | 4 | 2 | 1 | 1 | 70 | 7.2 | 11.6 |  |  |  |  |  | 4 |
| 489 | 12.16 | 1 | 3100 |  | 2 | 2 | 1 | 1 | 77 | 9.7 | 10.3 |  |  |  |  |  | 2 |
| 490 | 29.9 | 1 | 3200 |  | 3 | 2 | 1 | 1 | 93 | 13.2 | 12.9 | 244.37 | 9.61 | 35.79 | 54.95 | 0.94 | 1 |
| 491 | 9.53 | 2 | 3900 |  | 3 | 3 | 1 | 2 | 68.5 | 8.8 | 12.1 |  |  |  |  |  | 4 |
| 492 | 13.54 | 1 | 3100 |  | 2 | 3 | 1 | 1 | 75 | 9.8 | 10.1 |  |  |  |  |  | 2 |
| 493 | 12.55 | 1 | 3300 | 50 | 3 | 3 | 2 | 1 | 82.5 | 11.5 | 9.4 |  |  |  |  |  | 3 |
| 494 | 10.78 | 2 | 3100 |  | 3 | 2 | 1 | 1 | 69 | 8.7 | 11.5 |  |  |  |  |  | 3 |
| 495 | 5.52 | 1 | 3700 | 55 | 4 | 3 | 1 | 2 | 65.5 | 7.68 | 13 |  |  |  |  |  | 3 |
| 496 | 18.04 | 1 | 3100 |  | 2 | 2 | 1 | 1 | 82 | 11.5 | 11.5 |  |  |  |  |  | 3 |
| 497 | 31.77 | 1 | 3500 | 55 | 4 | 2 | 2 | 1 | 94.1 | 12.7 | 12.6 | 416.03 | 8.79 | 35.04 | 51.87 |  | 4 |
| 498 | 23.95 | 2 | 2100 |  | 2 | 2 | 1 | 1 | 76 | 9.8 | 11.3 |  |  |  |  |  | 5 |
| 499 | 25.56 | 1 | 3200 |  | 2 | 1 | 1 | 1 | 86.5 | 11.7 | 12.9 |  |  |  |  |  | 1 |
| 500 | 10.15 | 1 | 3000 |  | 3 | 3 | 2 | 1 | 67.5 | 7.7 | 11.8 |  |  |  |  |  | 3 |
| 501 | 9.17 | 1 | 3100 |  | 3 | 2 | 1 | 1 | 70 | 9.5 | 11.2 |  |  |  |  |  | 0 |
| 502 | 12.09 | 2 | 3650 |  | 4 | 2 | 2 | 2 | 68 | 5.8 | 9.4 |  |  |  |  |  | 5 |
| 503 | 4.99 | 1 | 3600 | 54 | 2 | 1 | 1 | 2 | 62 | 7.8 | 10.3 |  |  |  |  |  | 3 |
| 504 | 12.45 | 2 | 2750 | 48 | 3 | 3 | 1 | 1 | 71 | 7.8 | 12.1 |  |  |  |  |  | 4 |
| 505 | 11.4 | 1 |  |  | 3 | 2 | 1 | 1 | 73 | 8 | 11 |  |  |  |  |  | 2 |
| 506 | 6.67 | 2 | 3600 | 55 | 3 | 3 | 1 | 1 | 66 | 5.8 | 10.2 |  |  |  |  |  | 4 |
| 507 | 28.81 | 2 | 3000 |  |  |  |  |  | 85 | 10.5 | 12.7 |  |  |  |  |  | 0 |
| 508 | 33.35 | 1 | 3400 |  |  |  |  |  | 89 | 12.5 | 11.9 |  |  |  |  |  | 0 |
| 509 | 28.52 | 1 |  |  |  |  |  |  | 88 | 11.7 | 11.6 |  |  |  |  |  | 0 |
| 510 | 10.68 | 1 | 3000 |  | 3 | 3 | 1 | 1 | 72 | 7.7 | 11.2 |  |  |  |  |  | 1 |
| 511 | 7.43 | 1 | 3250 |  | 4 | 3 | 1 | 2 | 67 | 8.7 | 10.6 |  |  |  |  |  | 2 |
| 512 | 20.07 | 1 |  |  | 3 | 2 | 1 | 1 | 78 | 9.5 | 11.6 |  |  |  |  |  | 3 |
| 513 | 18.1 | 2 | 3100 |  | 2 | 2 | 2 | 1 | 77.5 | 8.9 | 11.3 |  |  |  |  |  | 2 |
| 514 | 19.35 | 1 | 3500 | 55 | 2 | 2 | 1 | 1 | 82 | 9.5 | 10.3 |  |  |  |  |  | 4 |
| 515 | 13.86 | 1 | 3150 | 50 | 3 | 2 | 1 | 1 | 72 | 8.8 | 10.1 |  |  |  |  |  | 0 |
| 516 | 18.96 | 2 | 3140 |  | 4 | 3 | 1 | 1 | 77 | 8.7 | 11.7 |  |  |  |  |  | 5 |
| 517 | 21.22 | 2 | 3300 | 50 | 2 | 3 | 1 | 1 | 79 | 9.7 | 13.8 |  |  |  |  |  | 4 |
| 518 | 8.05 | 1 |  |  | 2 | 3 | 1 | 1 | 63.5 | 8.8 | 13 |  |  |  |  |  | 1 |
| 519 | 16.2 | 2 |  |  | 3 | 3 | 2 | 1 | 74 | 11.2 | 12 |  |  |  |  |  | 3 |
| 520 | 11.24 | 1 | 3200 |  | 3 | 1 | 2 | 2 | 74 | 7.8 | 12.5 |  |  |  |  |  | 3 |
| 521 | 27.4 | 1 | 3100 |  | 3 | 3 | 1 | 1 | 82.5 | 9.9 | 13.1 |  |  |  |  |  | 0 |
| 522 | 23.82 | 2 | 2800 | 50 | 1 | 2 | 1 | 1 | 88 | 11.5 | 11.7 |  |  |  |  |  | 4 |
| 523 | 12.91 | 1 | 3100 |  | 3 | 3 | 2 | 1 | 74 | 9.5 | 11.7 |  |  |  |  |  | 4 |
| 524 | 13.34 | 1 | 3750 | 56 | 3 | 3 | 1 | 1 | 75 | 10.7 | 12.4 |  |  |  |  |  | 4 |
| 525 | 10.81 | 1 | 3600 | 55 | 3 | 3 | 2 | 1 | 68.5 | 7.3 | 11.1 |  |  |  |  |  | 2 |
| 526 | 13.27 | 2 | 3700 |  | 3 | 3 | 1 | 1 | 71.5 | 7.7 | 10.3 |  |  |  |  |  | 0 |
| 527 | 15.24 | 1 | 2500 |  | 3 | 2 | 2 | 1 | 79 | 8.5 | 14 |  |  |  |  |  | 2 |
| 528 | 5.88 | 2 | 3000 |  | 1 | 2 | 1 | 1 | 62 | 7.7 | 11.4 |  |  |  |  |  | 2 |
| 529 | 14.92 | 1 | 2600 | 48 | 2 | 3 | 1 | 1 | 74 | 8.8 | 11.3 |  |  |  |  |  | 5 |
| 530 | 14.92 | 1 | 2750 | 48 | 2 | 3 | 1 | 1 | 75 | 8.3 | 11.4 |  |  |  |  |  | 5 |
| 531 | 17.45 | 1 |  |  | 3 | 1 | 1 | 1 | 74 | 9.7 | 12.8 |  |  |  |  |  | 4 |
| 532 | 24.97 | 1 |  |  |  |  |  |  | 87 | 12.8 | 8.9 |  |  |  |  |  | 0 |
| 533 | 19.38 | 1 | 3400 | 55 | 3 | 1 | 2 | 1 | 77 | 9.8 | 10.3 |  |  |  |  |  | 3 |
| 534 | 16.49 | 2 | 3100 |  |  |  |  |  | 75 | 9.7 | 10.8 |  |  |  |  |  | 0 |
| 535 | 23.1 | 1 | 4000 |  | 3 | 2 | 1 | 1 | 85 | 12.68 | 12.5 |  |  |  |  |  | 3 |
| 536 | 23.82 | 1 |  |  | 2 | 2 | 1 | 1 | 76.5 | 10.18 | 12.5 |  |  |  |  |  | 2 |
| 537 | 20.8 | 1 | 3100 |  | 2 | 3 | 2 | 1 | 77 | 9.7 | 11.8 |  |  |  |  |  | 3 |
| 538 | 16.49 | 2 | 3100 |  |  |  |  |  | 76 | 8.7 | 12.3 |  |  |  |  |  | 0 |
| 539 | 5.55 | 2 | 3300 | 50 |  |  |  |  | 65 | 9.3 | 12.4 |  |  |  |  |  | 0 |
| 540 | 11.96 | 1 | 3600 | 50 |  |  |  |  | 76 | 9.7 | 12.3 |  |  |  |  |  | 0 |
| 541 | 4.01 | 2 | 3700 | 55 |  |  |  |  | 59 | 6.7 | 10.8 |  |  |  |  |  | 0 |
| 542 | 20.21 | 1 | 3350 | 49 |  |  |  |  | 79.3 | 9.25 | 13 |  |  |  |  |  | 0 |
| 543 | 14.69 | 2 | 3500 | 55 |  |  |  |  | 75.5 | 9.7 | 12.3 |  |  |  |  |  | 0 |
| 544 | 28.91 | 2 | 3000 |  |  |  |  |  | 81 | 10.75 | 12.9 |  |  |  |  |  | 0 |
| 545 | 6.57 | 1 | 3100 |  |  |  |  |  | 65 | 8.7 | 11.9 |  |  |  |  |  | 0 |
| 546 | 17.61 | 1 | 3000 |  |  |  |  |  | 76 | 9.7 | 11 |  |  |  |  |  | 0 |
| 547 | 2.5 | 1 | 4000 |  |  |  |  |  | 60 | 5.8 | 10.1 |  |  |  |  |  | 0 |
| 548 | 24.31 | 2 | 3000 |  |  |  |  |  | 80 | 9.5 | 12.1 |  |  |  |  |  | 0 |
| 549 | 12.25 | 1 | 3600 |  |  |  |  |  | 72 | 7.3 | 11.3 |  |  |  |  |  | 0 |
| 550 | 21.82 | 1 | 3100 |  |  |  |  |  | 79.5 | 8.5 | 10.2 |  |  |  |  |  | 0 |
| 551 | 21.75 | 1 | 3100 |  |  |  |  |  | 90 | 12.7 | 11.2 |  |  |  |  |  | 0 |
| 552 | 14.75 | 1 | 3500 |  |  |  |  |  | 81 | 10.8 | 11.3 |  |  |  |  |  | 0 |
| 553 | 36.01 | 2 |  |  |  |  |  |  | 90 | 10.5 | 12 |  |  |  |  |  | 0 |
| 554 | 21.72 | 2 | 3100 |  |  |  |  |  | 75 | 8.8 | 9.9 |  |  |  |  |  | 0 |
| 555 | 18.04 | 2 | 3000 |  |  |  |  |  | 75.5 | 7.8 | 11.7 |  |  |  |  |  | 0 |
| 556 | 17.48 | 1 |  |  |  |  |  |  | 76 | 9.3 | 11.3 |  |  |  |  |  | 0 |
| 557 | 28.52 | 2 | 3000 |  |  |  |  |  | 89.5 | 11.3 | 12.3 |  |  |  |  |  | 0 |
| 558 | 20.21 | 1 |  |  |  |  |  |  | 79 | 9.75 | 9.8 |  |  |  |  |  | 0 |
| 559 | 10.68 | 1 |  |  |  |  |  |  | 68 | 6.8 | 11.3 |  |  |  |  |  | 0 |
| 560 | 10.02 | 2 | 3700 |  |  |  |  |  | 68 | 8.7 | 9.8 |  |  |  |  |  | 0 |
| 561 | 13.24 | 1 |  |  |  |  |  |  | 71 | 8.8 | 11.9 |  |  |  |  |  | 0 |
| 562 | 16.39 | 1 | 3000 |  |  |  |  |  | 78 | 9.5 | 11.5 |  |  |  |  |  | 0 |
| 563 | 9.59 | 1 | 3750 |  |  |  |  |  | 70.5 | 9.5 | 10.9 |  |  |  |  |  | 0 |
| 564 | 21.85 | 2 | 3380 | 51 | 2 | 2 | 1 | 1 | 84 | 10.6 | 12.6 | 20.17 | 0.00 | 7.51 | 90.60 | 0.93 | 7 |
| 565 | 31.97 | 2 | 2800 |  | 3 | 3 | 1 | 1 | 88.2 | 10.74 | 12.2 | 185.51 | 27.43 | 23.31 | 37.17 | 0.83 | 2 |
| 566 | 32.23 | 1 | 3800 | 50 | 3 | 3 | 1 | 1 | 92.2 | 11.8 | 12.7 | 470.16 | 17.27 | 30.54 | 63.49 | 1.01 | 4 |
| 567 | 17.91 | 1 | 3000 | 50 | 3 | 3 | 1 | 1 | 79.6 | 9.32 | 13.4 |  |  | 16.66 | 93.23 | 0.92 | 4 |
| 568 | 16 | 2 | 2700 |  | 3 | 2 | 1 | 1 | 78.2 | 7.67 | 13 | 534.93 | 17.13 | 24.39 | 107.58 | 0.89 | 4 |
| 569 | 23.62 | 2 | 3100 |  | 4 | 3 | 1 | 1 | 79.1 | 9 | 13.4 | 308.86 | 5.43 | 10.81 | 85.12 | 1.04 | 3 |
| 570 | 10.74 | 1 | 3600 | 53 | 3 | 3 | 1 | 1 | 72.2 | 7 | 12.3 |  |  |  |  |  | 7 |
| 571 | 24.71 | 2 | 3000 |  | 3 | 2 | 2 | 1 | 82.5 | 9.87 | 13.4 | 199.02 | 10.20 | 24.30 | 105.42 | 1.06 | 6 |
| 572 | 27.47 | 1 | 3000 | 47 | 3 | 3 | 1 | 1 | 87 | 11.36 | 13.6 | 384.68 | 9.35 | 19.78 | 82.19 | 0.92 | 3 |
| 573 | 26.87 | 2 | 2400 |  | 3 | 2 | 2 | 2 | 81.5 | 9.965 | 12.9 | 396.16 | 4.70 | 7.19 | 82.19 | 1.03 | 2 |
| 574 | 14.82 | 2 | 3350 |  | 3 | 2 | 1 | 1 | 76.5 | 8.62 | 12.6 | 223.80 | 21.09 | 12.13 | 61.33 |  | 5 |
| 575 | 20.76 | 2 | 2500 |  | 3 | 3 | 1 | 1 | 77 | 9.415 | 12.3 | 468.06 | 7.73 | 6.14 | 44.98 | 0.99 | 3 |
| 576 | 6.11 | 1 | 2800 |  | 3 | 2 | 1 | 1 | 62.5 | 6.76 | 13.1 |  |  |  |  |  | 2 |
| 577 | 4.3 | 2 | 2900 | 48 | 3 | 3 | 2 | 1 | 62 | 6.295 | 13.3 |  |  |  |  |  | 0 |
| 578 | 4.07 | 2 | 3500 | 48 | 3 | 3 | 1 | 2 | 64 | 6.905 | 11.9 |  |  |  |  |  | 0 |
| 579 | 31.47 | 1 | 3600 |  | 3 | 2 | 1 | 1 | 91.5 | 14.29 | 13 | 233.57 | 37.22 | 18.19 | 56.22 | 0.82 | 4 |
| 580 | 5.09 | 2 | 2800 | 47 | 3 | 3 | 2 | 1 | 65.1 | 6.85 | 11.6 |  |  |  |  |  | 0 |
| 581 | 14.92 | 1 | 3400 | 48 | 3 | 4 | 1 | 1 | 75.6 | 9.605 | 12.3 | 627.21 | 20.12 | 42.77 | 95.64 | 1.01 | 5 |
| 582 | 18.6 | 2 | 4100 |  | 3 | 3 | 1 | 2 | 78.9 | 10.165 | 12.7 | 424.18 | 24.83 | 28.39 | 54.08 |  | 7 |
| 583 | 17.91 | 1 | 2950 | 47 | 4 | 3 | 1 | 1 | 81.5 | 10.58 | 13.2 | 277.74 | 32.02 | 37.00 | 78.35 | 0.86 | 3 |
| 584 | 9.33 | 2 | 2900 |  | 3 | 3 | 2 | 2 | 68.5 | 6.68 | 12.7 |  |  |  |  |  | 3 |
| 585 | 31.77 | 2 | 3300 |  | 2 | 2 | 1 | 1 | 90.1 | 14.6 | 14.3 | 510.32 | 13.52 | 21.65 | 72.29 | 1.04 | 6 |
| 586 | 14.49 | 2 | 2150 | 47 | 3 | 4 | 1 | 1 | 71.5 | 7.685 | 12.6 |  |  | 11.20 | 51.32 |  | 5 |
| 587 | 14.49 | 1 | 2850 | 48 | 3 | 4 | 2 | 1 | 78.5 | 10.15 | 10.9 |  |  | 4.12 | 46.21 | 0.90 | 4 |
| 588 | 3.55 | 2 | 2900 |  | 3 | 3 | 1 | 2 | 59.5 | 4.675 | 13 |  |  |  |  |  | 0 |
| 589 | 18.46 | 1 | 3200 |  | 3 | 3 | 1 | 1 | 86.3 | 10.025 | 12.7 |  |  | 13.26 | 108.31 | 1.32 | 4 |
| 590 | 10.87 | 1 | 2800 |  | 3 | 3 | 1 | 2 | 72.3 | 9.506 | 10.3 |  |  |  |  |  | 4 |
| 591 | 22.57 | 2 | 3150 |  | 3 | 4 | 2 | 1 | 89.3 | 12.445 | 13.1 | 301.32 | 10.86 | 16.86 | 78.08 | 1.16 | 3 |
| 592 | 2.76 | 2 | 3000 |  | 3 | 2 | 1 | 1 | 55 | 6.065 | 11.7 |  |  |  |  |  | 0 |
| 593 | 23.75 | 1 | 1900 |  | 3 | 3 | 2 | 1 | 80.2 | 8.565 | 12.2 | 356.27 | 13.16 | 28.27 | 75.10 | 0.81 | 5 |
| 594 | 7.29 | 1 | 3000 | 48 | 4 | 4 | 2 | 1 | 68.1 | 7.23 | 12.1 |  |  |  |  |  | 1 |
| 595 | 9.07 | 1 | 3200 | 47 | 2 | 3 | 2 | 1 | 68.5 | 9.045 | 14 |  |  |  |  |  | 4 |
| 596 | 25.03 | 1 | 2500 |  | 3 | 2 | 1 | 1 | 79 | 10.515 | 12.8 | 377.90 | 34.38 | 37.15 | 51.27 | 0.89 | 4 |
| 597 | 13.4 | 1 | 2600 |  | 2 | 2 | 1 | 1 | 71.9 | 7.671 | 11.9 | 398.49 | 17.66 | 11.92 | 62.85 | 0.82 | 4 |
| 598 | 24.84 | 1 | 3050 | 48 | 3 | 2 | 2 | 1 | 90.2 | 11.93 | 13.7 | 158.67 | 11.45 | 20.61 | 48.19 | 0.79 | 4 |
| 599 | 23.1 | 1 | 2700 | 47 | 4 | 3 | 1 | 1 | 77.1 | 8.2 | 12.4 | 482.90 | 16.81 | 20.19 | 63.49 | 0.88 | 3 |
| 600 | 25.33 | 1 |  |  | 2 | 2 | 1 | 2 | 77.3 | 9.52 | 11.7 | 587.91 | 7.65 | 20.77 | 64.45 | 0.76 | 6 |
| 601 | 2.79 | 1 | 3000 |  | 3 | 1 | 1 | 1 | 56.2 | 5.485 | 9.8 |  |  |  |  |  | 0 |
| 602 | 2.83 | 1 | 2900 |  | 4 | 2 | 1 | 1 | 57.8 | 4.58 | 11.4 |  |  |  |  |  | 0 |
| 603 | 26.09 | 1 | 3300 |  | 3 | 3 | 1 | 1 | 80.1 | 8.925 | 11.8 | 350.24 | 16.34 | 17.65 | 58.40 | 0.79 | 3 |
| 604 | 14.06 | 2 | 2500 | 47 | 3 | 3 | 1 | 1 | 71.1 | 8.135 | 11.9 | 346.39 | 37.28 | 8.70 | 54.66 | 0.96 | 6 |
| 605 | 24.44 | 2 |  |  | 2 | 2 | 1 | 1 | 84.2 | 10.325 | 12.8 | 226.23 | 20.18 | 38.91 | 55.69 | 0.96 | 3 |
| 606 | 13.01 | 2 | 3000 |  | 3 | 3 | 1 | 1 | 75.9 | 9.75 | 11.7 |  |  |  |  |  | 3 |
| 607 | 13.11 | 2 | 3100 |  | 4 | 2 | 1 | 2 | 73.1 | 9.75 | 13.5 | 122.58 | 22.11 | 7.50 | 74.27 | 0.88 | 2 |
| 608 | 1.81 | 1 | 3850 | 46 | 3 | 3 | 2 | 1 | 58.1 | 5.31 | 10.3 |  |  |  |  |  | 1 |
| 609 | 9.99 | 2 | 3300 |  | 3 | 2 | 2 | 1 | 66.2 | 6.92 | 11.2 |  |  |  |  |  | 3 |
| 610 | 14.98 | 1 | 3150 |  | 2 | 2 | 1 | 1 | 71.7 | 8.13 | 11.6 | 262.40 | 10.98 | 11.76 | 56.22 | 0.84 | 4 |
| 611 | 9.4 | 2 | 3200 | 45 | 3 | 3 | 1 | 1 | 70.1 | 7.59 | 12.3 |  |  |  |  |  | 2 |
| 612 | 19.09 | 1 |  |  | 2 | 3 | 1 | 1 | 76.5 | 9.09 | 11 | 342.15 | 7.57 | 4.50 | 56.22 | 1.18 | 3 |
| 613 | 25.76 | 1 | 2950 |  | 3 | 3 | 1 | 1 | 85 | 12.13 | 13.9 |  |  | 22.59 | 54.16 | 0.90 | 6 |
| 614 | 14.03 | 1 | 3200 |  | 3 | 3 | 2 | 1 | 74.1 | 9.2 | 11.6 |  |  | 34.66 | 74.69 | 0.75 | 3 |
| 615 | 32.72 | 1 | 3050 |  | 2 | 3 | 1 | 1 | 84 | 12.7 | 12.3 | 231.11 | 15.85 | 7.95 | 58.08 | 1.08 | 3 |
| 616 | 14.88 | 2 | 2800 | 45 | 3 | 2 | 1 | 1 | 73 | 7.36 | 12.8 | 237.46 | 6.87 | 4.96 | 62.52 | 1.05 | 4 |
| 617 | 21.85 | 2 | 3400 |  | 3 | 3 | 1 | 1 | 80.5 | 11.7 | 13.6 | 258.36 | 10.72 | 14.47 | 67.81 | 1.01 | 4 |
| 618 | 7.2 | 2 | 2800 | 47 | 3 | 3 | 1 | 1 | 70 | 9.5 | 10.7 |  |  | 24.69 | 53.87 |  | 1 |
| 619 | 27.3 | 1 | 3000 | 44 | 3 | 3 | 1 | 1 | 80 | 10.26 | 14.6 |  |  |  |  |  | 4 |
| 620 | 30.72 | 1 | 3800 | 50 | 3 | 2 | 1 | 1 | 84.5 | 11.73 | 12.9 | 344.69 | 8.25 | 27.39 | 69.15 | 1.45 | 3 |
| 621 | 2.4 | 1 | 2500 |  | 3 | 3 | 1 | 1 | 57 | 5.4 | 10 |  |  |  |  |  | 0 |
| 622 | 32.59 | 1 | 3300 |  | 2 | 2 | 1 | 1 | 81.5 | 9.83 | 12.6 | 173.24 | 16.24 | 31.98 | 62.52 | 1.20 | 2 |
| 623 | 32.95 | 2 | 3000 |  | 2 | 2 | 1 | 1 | 84.5 | 10.3 | 15.4 | 325.06 | 10.54 | 34.37 | 43.23 | 0.73 | 2 |
| 624 | 29.34 | 2 | 2900 |  | 3 | 2 | 2 | 1 | 84.5 | 10.1 | 13.5 |  |  | 19.30 | 47.84 |  | 1 |
| 625 | 7.49 | 1 | 3100 | 48 | 3 | 3 | 1 | 1 | 59 | 4.2 | 12.8 |  |  |  |  |  | 0 |
| 626 | 15.54 | 1 | 3000 | 48 | 3 | 2 | 1 | 2 | 66 | 8.29 | 10.6 |  |  |  |  |  | 0 |
| 627 | 15.41 | 1 | 3500 |  | 3 | 2 | 1 | 1 | 77.7 | 9.4 | 11.5 |  |  | 2.80 | 79.41 |  | 1 |
| 628 | 5.75 | 2 |  |  | 2 | 2 | 1 | 1 | 65.2 | 6.1 | 13 |  |  |  |  |  | 0 |
| 629 | 31.01 | 1 |  |  | 2 | 2 | 1 | 1 | 82.6 | 13.26 | 12.2 | 113.64 | 4.45 | 26.33 | 52.69 | 1.04 | 1 |
| 630 | 1.71 | 1 | 2900 | 40 | 4 | 2 | 1 | 1 | 70.3 | 7.37 | 10.6 |  |  |  |  |  | 2 |
| 631 | 4.21 | 1 | 3200 | 43 | 2 | 2 | 1 | 1 | 73.5 | 9.216 | 11 | 243.15 | 11.38 | 13.47 | 72.29 | 1.05 | 2 |
| 632 | 4.07 | 2 | 2800 | 40 | 2 | 2 | 2 | 1 | 57.3 | 4.693 | 13.1 |  |  |  |  |  | 0 |
| 633 | 24.44 | 2 |  |  | 2 | 2 | 1 | 1 | 80.3 | 10.14 | 13.9 | 118.40 | 10.06 | 13.85 | 65.78 | 1.14 | 3 |
| 634 | 3.35 | 2 | 2700 | 50 | 2 | 2 | 1 | 2 | 62.3 | 5.596 | 13.2 |  |  |  |  |  | 0 |
| 635 | 5.78 | 1 | 3000 |  | 2 | 2 | 1 | 2 | 66.3 | 7.14 | 12.8 |  |  |  |  |  | 2 |
| 636 | 1.81 | 2 | 3000 |  | 2 | 3 | 1 | 1 | 54.3 | 4.418 | 13 |  |  |  |  |  | 0 |
| 637 | 15.74 | 2 |  |  | 2 | 2 | 2 | 1 | 70.2 | 8.9 | 13.6 |  |  |  |  |  | 3 |
| 638 | 24.44 | 2 | 2950 |  | 3 | 2 | 2 | 1 | 81.5 | 11.03 | 12.8 |  |  | 24.10 | 58.69 | 1.27 | 3 |
| 639 | 5.36 | 2 |  |  | 2 | 2 | 1 | 2 | 60.4 | 6.35 | 12.5 |  |  |  |  |  | 0 |
| 640 | 21.16 | 1 | 3150 |  | 4 | 3 | 1 | 1 | 82.4 | 9.93 | 11.9 | 370.31 | 24.05 | 40.06 | 39.44 | 1.31 | 4 |
| 641 | 18.37 | 1 |  |  | 2 | 2 | 1 | 1 | 83.5 | 9.35 | 13.6 |  |  | 28.21 | 49.69 | 1.20 | 2 |
| 642 | 5.29 | 1 | 3000 | 42 | 2 | 1 | 1 | 1 | 66.5 | 5.72 | 13.7 |  |  |  |  |  | 0 |
| 643 | 32.16 | 1 | 2400 |  | 2 | 2 | 1 | 1 | 86.2 | 10.985 | 12.4 | 250.34 | 10.80 | 13.09 | 60.13 | 1.15 | 4 |
| 644 | 15.34 | 1 | 2100 |  | 3 | 3 | 1 | 1 | 74.2 | 7.67 | 11.3 | 366.77 | 10.99 | 2.52 | 73.47 | 1.15 | 5 |
| 645 | 12.58 | 1 | 3000 | 48 | 3 | 3 | 1 | 1 | 76.3 | 9.445 | 11.1 | 625.86 | 2.75 | 7.94 | 74.69 | 1.04 | 3 |
| 646 | 9.4 | 1 | 3500 |  | 2 | 2 | 1 | 1 | 70.6 | 9.8 | 11.8 |  |  |  |  |  | 4 |
| 647 | 21.03 | 1 | 3500 | 50 | 2 | 3 | 1 | 1 | 82.8 | 10.855 | 12.5 | 328.77 | 9.16 | 17.12 | 75.93 | 1.07 | 4 |
| 648 | 3.25 | 1 | 3150 | 48 | 3 | 3 | 1 | 2 | 62.5 | 7.41 | 11.8 |  |  |  |  |  | 0 |
| 649 | 0.89 | 2 | 2750 |  | 2 | 2 | 1 | 1 | 52.2 | 3.975 | 12.7 |  |  |  |  |  | 0 |
| 650 | 4.3 | 2 | 3600 |  | 2 | 2 | 1 | 1 | 68.2 | 8.315 | 15.1 |  |  |  |  |  | 0 |
| 651 | 11.66 | 1 | 3300 |  | 3 | 3 | 1 | 1 | 71.5 | 8.31 | 11.6 |  |  |  |  |  | 1 |
| 652 | 20.21 | 1 | 3500 |  | 3 | 2 | 1 | 1 | 82.1 | 10.85 | 12.1 | 255.79 | 6.75 | 7.59 | 94.41 | 1.16 | 4 |
| 653 | 25.69 | 1 | 3500 |  | 2 | 3 | 1 | 1 | 89.1 | 13.67 | 13.9 | 337.10 | 7.97 | 10.76 | 35.49 | 1.24 | 5 |
| 654 | 13.9 | 2 | 3000 |  | 3 | 3 | 1 | 1 | 76.5 | 8.955 | 12 | 460.78 | 7.17 | 5.86 | 68.19 | 0.88 | 4 |
| 655 | 15.93 | 2 | 3200 | 48.5 | 4 | 3 | 1 | 1 | 78.2 | 9.5 | 11.7 | 460.26 | 6.61 | 24.38 | 49.04 | 1.17 | 4 |
| 656 | 15.97 | 1 | 2800 | 47 | 3 | 4 | 2 | 1 | 75.6 | 7.625 | 12.4 | 91.36 | 9.96 | 15.31 | 63.49 | 0.95 | 4 |
| 657 | 2.79 | 1 | 2500 | 46 | 2 | 4 | 1 | 2 | 58.5 | 6.95 | 12 |  |  |  |  |  | 1 |
| 658 | 18.5 | 2 | 3200 |  | 3 | 4 | 1 | 2 | 77.5 | 8.85 | 12.7 | 587.28 | 32.69 | 18.28 | 93.81 | 1.08 | 5 |
| 659 | 11.01 | 1 | 3100 | 48 | 3 | 3 | 1 | 1 | 76.1 | 10.1 | 11.5 |  |  |  |  |  | 3 |
| 660 | 1.74 | 2 | 3000 |  | 3 | 2 | 1 | 2 | 56 | 4.4 | 14 |  |  |  |  |  | 0 |
| 661 | 32.16 | 1 | 3500 |  | 2 | 2 | 1 | 1 | 92.2 | 12.32 | 13.6 | 117.21 | 9.86 | 23.25 | 60.13 | 0.89 | 4 |
| 662 | 21.55 | 2 | 2500 |  | 3 | 3 | 1 | 1 | 81.1 | 8.96 | 12.2 |  |  | 11.92 | 82.67 | 1.08 | 3 |
| 663 | 8.31 | 1 | 3500 |  | 2 | 2 | 1 | 1 | 73.5 | 8.69 | 11.9 |  |  |  |  |  | 4 |
| 664 | 35.65 | 2 | 2950 | 49 | 3 | 3 | 1 | 1 | 93.5 | 13.36 | 14.1 | 260.19 | 14.56 | 36.40 | 20.52 | 1.39 | 5 |
| 665 | 13.14 | 1 | 3200 |  | 3 | 3 | 2 | 1 | 71 | 8.7 | 11.7 |  | 0.00 | 7.92 | 54.66 | 1.64 | 2 |
| 666 | 13.5 | 1 | 3100 | 42 | 3 | 3 | 1 | 1 | 75.9 | 10.13 | 12.1 | 196.36 | 16.02 | 14.28 | 103.83 | 0.91 | 4 |
| 667 | 11.2 | 1 | 3300 |  | 2 | 2 | 1 | 1 | 68 | 9.1 | 11.1 |  |  |  |  |  | 2 |
| 668 | 12.16 | 1 | 3200 |  | 2 | 2 | 1 | 2 | 70 | 9.19 | 11.9 |  |  |  |  |  | 3 |
| 669 | 30.29 | 2 | 3050 |  | 2 | 2 | 1 | 1 | 82 | 9.27 | 13.7 | 261.30 | 17.50 | 15.04 | 68.47 | 0.97 | 2 |
| 670 | 23.89 | 2 | 2300 |  | 3 | 2 | 2 | 1 | 76 | 8.63 | 12.4 | 189.44 | 10.44 | 16.72 | 70.18 | 1.14 | 5 |
| 671 | 30.62 | 1 |  |  | 2 | 2 | 1 | 1 | 89.1 | 14.02 | 13.7 |  |  | 14.34 | 50.72 |  | 5 |
| 672 | 5.06 | 1 | 3600 |  | 3 | 3 | 1 | 1 | 66.5 | 8.35 | 12.8 |  |  |  |  |  | 1 |
| 673 | 22.05 | 1 | 3350 |  | 3 | 2 | 1 | 1 | 83.5 | 12.49 | 12.8 | 158.05 | 7.78 | 23.92 | 56.29 |  | 4 |
| 674 | 28.94 | 2 | 3000 | 46 | 3 | 3 | 1 | 2 | 85 | 10.41 | 12.3 | 436.96 | 4.26 | 10.05 | 50.08 | 0.87 | 2 |
| 675 | 8.57 | 2 | 3150 | 48 | 3 | 3 | 1 | 1 | 68.1 | 7.71 | 12.9 |  |  |  |  |  | 1 |
| 676 | 26.74 | 2 | 3100 |  | 4 | 3 | 2 | 1 | 86.2 | 10.63 | 12.8 | 454.10 | 12.97 | 49.84 | 93.21 | 0.82 | 4 |
| 677 | 21.42 | 1 | 3100 |  | 3 | 2 | 2 | 1 | 80.1 | 9.38 | 12.8 |  |  | 7.78 | 66.82 | 0.93 | 6 |
| 678 | 8.08 | 1 | 3250 | 51.5 | 3 | 4 | 1 | 1 | 70.2 | 8.26 | 10.3 |  |  |  |  |  | 5 |
| 679 | 12.62 | 2 | 2700 |  | 3 | 2 | 1 | 2 | 76.1 | 8.26 | 12.1 |  |  |  |  |  | 4 |
| 680 | 17.61 | 2 | 3200 | 45.5 | 3 | 2 | 1 | 1 | 77.1 | 8.03 | 11.4 | 251.79 | 12.75 | 22.60 | 99.48 | 1.14 | 3 |
| 681 | 2.53 | 1 | 3500 | 48 | 3 | 3 | 1 | 1 | 60.1 | 6.64 | 12.1 |  |  |  |  |  | 0 |
| 682 | 17.38 | 1 | 3450 |  | 3 | 4 | 1 | 2 | 80.2 | 9.65 | 13.8 | 245.30 | 7.13 | 22.09 | 103.83 | 0.93 | 5 |
| 683 | 30.75 | 2 | 3100 |  | 3 | 4 | 2 | 1 | 90.1 | 11.62 | 13.1 | 184.86 | 13.39 | 16.24 | 74.18 | 0.99 | 5 |
| 684 | 23.29 | 2 | 2800 | 49 | 3 | 3 | 1 | 1 | 89 | 8.23 | 13.5 | 300.53 | 9.66 | 16.83 | 78.95 | 1.11 | 5 |
| 685 | 8.34 | 2 | 3105 | 46 | 3 | 2 | 1 | 1 | 71.1 | 7.48 | 13.7 |  |  |  |  |  | 3 |
| 686 | 24.84 | 1 | 2800 | 46 | 3 | 3 | 1 | 1 | 82.2 | 8.89 | 12.8 |  |  | 16.22 | 71.59 | 1.00 | 4 |
| 687 | 18.27 | 2 | 3000 |  | 4 | 3 | 1 | 1 | 77.2 | 8.96 | 12.9 | 318.12 | 12.98 | 28.35 | 81.08 | 1.16 | 7 |
| 688 | 11.17 | 2 | 3000 | 46 | 2 | 2 | 2 | 1 | 73 | 8.4 | 13 |  |  |  |  |  | 3 |
| 689 | 12.62 | 1 | 3200 |  | 3 | 3 | 1 | 1 | 75.2 | 10.09 | 10.2 |  |  |  |  |  | 5 |
| 690 | 6.54 | 1 | 3300 | 50 | 3 | 3 | 1 | 1 | 64.5 | 7.505 | 10.9 |  |  |  |  |  | 0 |
| 691 | 7.2 | 2 | 2500 | 40 | 3 | 3 | 1 | 1 | 65 | 6.065 | 12 |  |  |  |  |  | 2 |
| 692 | 3.88 | 1 | 3200 | 50 | 3 | 1 | 1 | 1 | 59.1 | 5.59 | 13.8 |  |  |  |  |  | 0 |
| 693 | 7.62 | 2 | 2800 |  | 2 | 3 | 1 | 1 | 68.9 | 6.985 | 13 |  |  |  |  |  | 5 |
| 694 | 17.41 | 2 | 1800 | 40 | 3 | 2 | 1 | 1 | 78.5 | 9.605 | 11.2 |  |  |  |  |  | 4 |
| 695 | 31.57 | 2 | 3950 |  | 3 | 3 | 2 | 1 | 84.9 | 11.585 | 13.3 | 401.44 | 29.06 | 23.30 | 67.95 | 0.78 | 2 |
| 696 | 15.8 | 1 | 4000 |  | 3 | 3 | 1 | 2 | 82.8 | 10.14 | 12.9 |  |  | 11.66 | 52.70 | 0.75 | 3 |
| 697 | 18.73 | 2 | 3200 | 47 | 3 | 3 | 1 | 1 | 80.5 | 11.12 | 13.7 | 359.30 | 10.22 | 21.37 | 65.66 | 1.04 | 4 |
| 698 | 5.45 | 2 | 2500 |  | 3 | 4 | 1 | 1 | 64.5 | 6.565 | 13.9 |  |  |  |  |  | 2 |
| 699 | 28.65 | 2 | 3500 |  | 3 | 3 | 1 | 1 | 86.9 | 12.15 | 12.7 | 579.84 | 15.48 | 42.73 | 57.06 | 0.96 | 3 |
| 700 | 7.2 | 1 | 3100 | 55 | 3 | 3 | 1 | 1 | 69 | 8.13 | 12.4 |  |  |  |  |  | 3 |
| 701 | 5.68 | 2 | 3400 | 49 | 3 | 3 | 2 | 1 | 65.3 | 7.565 | 11.6 |  |  |  |  |  | 2 |
| 702 | 9.1 | 2 | 3200 |  | 3 | 3 | 1 | 1 | 70.5 | 9 | 11.8 |  |  |  |  |  | 3 |
| 703 | 31.9 | 1 | 3000 | 55 | 3 | 3 | 2 | 1 | 88.5 | 12.375 | 12.8 | 411.75 | 10.39 | 18.42 | 61.70 | 0.76 | 5 |
| 704 | 7.75 | 1 | 3150 | 49 | 3 | 4 | 1 | 2 | 68.2 | 8.175 | 11.9 |  |  |  |  |  | 2 |
| 705 | 23.26 | 2 | 3200 |  | 3 | 3 | 1 | 1 | 90.6 | 10.065 | 12.2 | 144.45 | 5.00 | 16.72 | 52.47 | 0.75 | 6 |
| 706 | 25.53 | 2 | 3000 | 47 | 3 | 3 | 2 | 1 | 82.8 | 10.185 | 13.1 | 367.83 | 8.39 | 21.15 | 80.66 | 0.80 | 6 |
| 707 | 18.17 | 2 | 3600 |  | 3 | 3 | 1 | 1 | 83 | 10.42 | 10.9 | 757.50 | 6.93 | 4.89 | 80.23 | 1.03 | 5 |
| 708 | 4.34 | 2 | 3650 |  | 3 | 4 | 1 | 2 | 65.6 | 6.685 | 11.2 |  |  |  |  |  | 0 |
| 709 | 22.31 | 1 | 3700 |  | 3 | 3 | 1 | 2 | 85.6 | 12.8 | 13.3 | 363.07 | 11.22 | 14.64 | 64.10 | 0.84 | 5 |
| 710 | 6.97 | 2 | 3000 |  | 3 | 3 | 1 | 1 | 66.5 | 7.65 | 10.6 |  |  |  |  |  | 0 |
| 711 | 2.37 | 2 | 2900 | 48 | 3 | 3 | 1 | 1 | 56.2 | 4.88 | 11.1 |  |  |  |  |  | 1 |
| 712 | 19.12 | 1 | 3350 | 50 | 2 | 2 | 1 | 1 | 82.3 | 11.025 | 12 | 133.11 | 13.92 | 13.05 | 58.37 | 0.97 | 4 |
| 713 | 1.54 | 2 | 2600 |  | 3 | 3 | 1 | 1 | 55.5 | 4.065 | 13.9 |  |  |  |  |  | 0 |
| 714 | 1.12 | 2 | 3250 | 48 | 6 | 3 | 1 | 1 | 53 | 3.745 | 12.4 |  |  |  |  |  | 0 |
| 715 | 5.52 | 1 | 3050 |  | 4 | 3 | 2 | 1 | 69 | 7.4 | 11.9 |  |  |  |  |  | 3 |
| 716 | 6.6 | 2 | 2700 |  | 2 | 3 | 1 | 1 | 60.4 | 5.19 | 11.2 |  |  |  |  |  | 2 |
| 717 | 6.7 | 2 | 2600 |  | 3 | 2 | 1 | 1 | 63 | 6.62 | 10.5 |  |  |  |  |  | 2 |
| 718 | 7.95 | 2 | 3100 |  | 3 | 2 | 2 | 1 | 69.5 | 8.22 | 10.7 |  |  |  |  |  | 0 |
| 719 | 3.98 | 1 | 3900 |  | 3 | 3 | 1 | 1 | 64 | 6.58 | 11.1 |  |  |  |  |  | 0 |
| 720 | 6.31 | 2 | 2700 |  | 2 | 2 | 1 | 1 | 64.8 | 6.11 | 10.3 |  |  |  |  |  | 2 |
| 721 | 17.45 | 1 | 3400 |  | 3 | 3 | 1 | 1 | 78 | 9.77 | 13.3 | 413.58 | 8.56 | 38.24 | 58.53 | 0.79 | 4 |
| 722 | 16.43 | 1 | 3200 | 50 | 4 | 3 | 1 | 1 | 79.2 | 9.17 | 12.7 | 288.83 | 11.13 | 4.79 | 72.12 | 1.09 | 5 |
| 723 | 16.2 | 2 | 2600 |  | 3 | 3 | 1 | 1 | 74.3 | 8.01 | 12.5 | 562.38 | 12.06 | 9.43 | 95.07 | 1.03 | 3 |
| 724 | 20.17 | 1 | 3350 |  | 3 | 3 | 1 | 1 | 80.9 | 10.645 | 13.5 | 253.97 | 7.74 | 15.19 | 83.07 | 0.89 | 6 |
| 725 | 21.68 | 2 | 2950 |  | 3 | 3 | 1 | 1 | 78 | 8.9 | 14.3 | 364.13 | 13.04 | 6.35 | 67.79 | 0.88 | 7 |
| 726 | 19.84 | 2 | 3200 |  | 3 | 1 | 1 | 1 | 81 | 11.4 | 13 | 209.42 | 5.27 | 32.33 | 73.27 | 1.25 | 3 |
| 727 | 19.35 | 2 | 3600 |  | 3 | 3 | 1 | 1 | 82.1 | 10.875 | 12.4 | 324.65 | 14.41 | 7.03 | 86.46 | 1.35 | 2 |
| 728 | 25.2 | 1 | 2700 | 48 | 3 | 3 | 1 | 1 | 82 | 9.2 | 14.2 | 78.33 | 6.72 | 26.13 | 70.63 | 0.86 | 4 |
| 729 | 28.06 | 2 | 3750 |  | 3 | 3 | 1 | 1 | 90.2 | 11.29 | 12.9 |  |  | 30.64 | 95.65 | 1.07 | 4 |
| 730 | 31.28 | 1 | 3800 |  | 3 | 3 | 1 | 1 | 83.3 | 10.41 | 12.2 | 242.43 | 16.28 | 22.19 | 74.05 | 0.98 | 3 |
| 731 | 32.39 | 2 | 3150 |  | 3 | 3 | 1 | 1 | 92.8 | 11.475 | 13.3 | 410.24 | 12.53 | 31.71 | 109.28 | 0.84 | 5 |
| 732 | 32.85 | 2 | 2600 |  | 3 | 3 | 1 | 1 | 87.1 | 10.5 | 13.8 | 290.76 | 10.19 | 24.13 | 28.90 | 0.87 | 5 |
| 733 | 31.67 | 1 | 3500 |  | 3 | 2 | 1 | 1 | 86.3 | 11.71 | 13.1 | 234.63 | 8.83 | 21.33 | 67.79 | 0.82 | 3 |
| 734 | 31.51 | 2 | 2350 | 47 | 3 | 3 | 1 | 1 | 88.2 | 12.125 | 13.7 | 255.06 | 12.17 | 22.26 | 81.23 | 0.81 | 4 |
| 735 | 29.17 | 2 | 2900 | 47 | 3 | 2 | 1 | 1 | 87.1 | 11.18 | 13.9 | 177.41 | 11.46 | 11.87 | 83.55 | 0.94 | 5 |
| 736 | 19.88 | 2 | 3750 |  | 3 | 3 | 1 | 1 | 81.2 | 10.5 | 13.5 | 524.91 | 8.57 | 16.01 | 60.85 | 1.14 | 5 |
| 737 | 32.46 | 2 | 2900 |  | 3 | 3 | 1 | 1 | 83.3 | 9.345 | 13 | 489.56 | 16.95 | 25.00 | 90.84 | 0.96 | 4 |
| 738 | 13.93 | 2 | 2600 |  | 2 | 3 | 1 | 1 | 67 | 6.51 | 11.8 | 544.42 | 7.31 | 22.82 | 55.06 | 0.85 | 3 |
| 739 | 19.09 | 2 | 2800 | 47 | 2 | 3 | 1 | 1 | 76 | 10.6 | 12.9 | 218.92 | 8.64 | 11.16 | 76.44 | 0.82 | 5 |
| 740 | 17.91 | 1 | 2700 | 47 | 3 | 3 | 1 | 1 | 74.5 | 7.6 | 11.8 | 114.06 | 11.13 | 14.91 | 81.55 | 0.87 | 7 |
| 741 | 6.51 | 2 | 3400 | 56 | 3 | 3 | 1 | 1 | 73 | 8.005 | 13.2 |  |  |  |  |  | 3 |
| 742 | 34.83 | 1 | 2500 |  | 3 | 3 | 1 | 1 | 89 | 11.95 | 13.3 | 149.95 | 9.79 | 23.58 | 72.19 | 1.06 | 2 |
| 743 | 7.49 | 2 | 4100 |  | 4 | 3 | 1 | 1 | 73 | 10.39 | 11.4 |  |  |  |  |  | 4 |
| 744 | 15.28 | 1 | 2950 | 48 | 3 | 4 | 1 | 2 | 72 | 8 | 13 | 586.19 | 16.19 | 17.88 | 91.38 | 0.99 | 5 |
| 745 | 8.9 | 1 | 2750 | 47 | 3 | 3 | 1 | 1 | 69 | 7.4 | 11.7 |  |  |  |  |  | 5 |
| 746 | 3.94 | 1 | 3300 |  | 3 | 3 | 1 | 1 | 64 | 7.2 | 12.4 |  |  |  |  |  | 2 |
| 747 | 14.39 | 2 | 3000 | 55 | 3 | 3 | 1 | 1 | 72 | 9 | 11.6 | 86.99 | 20.27 | 6.27 | 81.68 | 1.07 | 3 |
| 748 | 28.52 | 1 | 3100 | 46 | 3 | 3 | 1 | 1 | 87 | 12.725 | 13.2 | 171.24 | 10.23 | 32.21 | 80.33 | 0.71 | 5 |
| 749 | 32.03 | 2 |  |  | 4 | 3 | 1 | 1 | 92 | 12.72 | 12.5 | 312.71 | 10.40 | 24.90 | 64.16 | 1.14 | 5 |
| 750 | 19.94 | 2 | 3100 |  | 3 | 2 | 1 | 1 | 80 | 10.6 | 12.2 | 460.27 | 9.29 | 55.43 | 73.66 | 0.93 | 1 |
| 751 | 10.22 | 2 | 2400 |  | 3 | 2 | 1 | 1 | 67 | 7.1 | 10.4 |  |  |  |  |  | 4 |
| 752 | 6.08 | 1 | 3200 | 50 | 3 | 3 | 2 | 1 | 64 | 6.55 | 11.5 |  |  |  |  |  | 1 |
| 753 | 10.12 | 1 | 2500 | 45 | 2 | 3 | 2 | 1 | 68.5 | 6.025 | 11.6 |  |  |  |  |  | 2 |
| 754 | 27.76 | 2 | 3200 |  | 3 | 2 | 1 | 1 | 80 | 9.19 | 12.9 | 412.80 | 6.79 | 16.16 | 90.60 | 0.87 | 5 |
| 755 | 35.81 | 2 | 3000 |  | 3 | 3 | 1 | 1 | 84 | 9.8 | 13.5 | 671.09 | 10.17 | 18.90 | 98.03 | 0.89 | 3 |
| 756 | 27.47 | 1 | 2800 |  | 2 | 2 | 1 | 1 | 76.5 | 8.485 | 12.1 | 804.13 | 17.79 | 11.75 | 74.05 | 1.05 | 4 |
| 757 | 22.93 | 1 | 3500 | 57 | 3 | 3 | 1 | 1 | 84 | 12.2 | 12.8 | 222.39 | 8.50 | 18.55 | 99.16 | 0.85 | 4 |
| 758 | 8.67 | 2 | 4450 |  | 3 | 4 | 1 | 1 | 74.5 | 9.475 | 11.3 |  |  |  |  |  | 3 |
| 759 | 11.79 | 2 | 3100 |  | 3 | 3 | 1 | 2 | 68.5 | 8.298 | 11.7 |  |  |  |  |  | 3 |
| 760 | 11.24 | 2 | 3600 | 50 | 3 | 3 | 1 | 1 | 70.5 | 8.48 | 11.7 |  |  |  |  |  | 3 |
| 761 | 7.29 | 2 | 3400 |  | 3 | 4 | 2 | 2 | 66 | 7.665 | 12.8 |  |  |  |  |  | 1 |
| 762 | 33.35 | 1 | 3500 |  | 4 | 3 | 1 | 1 | 91 | 12.23 | 11.9 | 194.39 | 9.57 | 47.14 | 53.16 | 0.80 | 4 |
| 763 | 33.38 | 2 | 2800 |  | 3 | 3 | 1 | 1 | 90 | 11.8 | 11.5 | 197.66 | 12.52 | 34.81 | 54.81 | 0.92 | 4 |
| 764 | 24.28 | 1 | 3300 |  | 3 | 4 | 1 | 1 | 86 | 11.76 | 12.4 | 229.08 | 11.89 | 8.88 | 57.06 | 0.80 | 4 |
| 765 | 32.99 | 1 | 3000 |  | 3 | 3 | 1 | 1 | 88 | 11.28 | 12.9 | 325.70 | 17.93 | 21.53 | 264.51 | 0.83 | 5 |
| 766 | 14.03 | 1 | 2650 |  | 3 | 2 | 1 | 1 | 70 | 7.275 | 12 | 261.83 | 10.23 | 6.91 | 57.06 | 1.09 | 3 |
| 767 | 20.11 | 2 | 3500 |  | 2 | 4 | 1 | 1 | 78.5 | 10.6 | 12.6 | 451.22 | 11.92 | 25.24 | 64.71 | 1.07 | 5 |
| 768 | 12.98 | 1 | 3100 |  | 3 | 3 | 1 | 1 | 70.5 | 9.7 | 11.4 | 162.40 | 6.63 | 21.00 | 71.45 | 1.03 | 3 |
| 769 | 5.59 | 1 | 3500 | 49 | 4 | 3 | 1 | 1 | 67.5 | 8.3 | 12.2 |  |  |  |  |  | 1 |
| 770 | 33.68 | 2 | 3000 | 56 | 3 | 3 | 1 | 1 | 87.5 | 10.745 | 13.3 | 400.93 | 9.17 | 29.00 | 66.95 | 1.07 | 4 |
| 771 | 5.03 | 2 | 3250 | 47 | 3 | 2 | 2 | 1 | 67 | 7.34 | 13.3 |  |  |  |  |  | 1 |
| 772 | 15.57 | 2 | 2900 | 54 | 3 | 3 | 1 | 1 | 75.2 | 8.93 | 11 | 174.96 | 5.32 | 7.92 | 83.38 | 1.11 | 4 |
| 773 | 33.48 | 1 | 3500 |  | 3 | 3 | 1 | 2 | 90 | 11.8 | 11.4 | 353.69 | 14.78 | 21.47 | 64.71 | 1.30 | 4 |
| 774 | 6.44 | 2 | 3400 | 48 | 4 | 3 | 1 | 1 | 63.5 | 7.42 | 11.7 |  |  |  |  |  | 3 |
| 775 | 32.3 | 1 | 2800 | 48 | 3 | 3 | 1 | 1 | 85 | 10.5 | 12.4 | 397.87 | 12.66 | 19.70 | 48.61 | 0.87 | 5 |
| 776 | 15.01 | 1 | 3100 |  | 3 | 3 | 1 | 1 | 75.5 | 8.5 | 9.7 | 190.81 | 14.55 | 6.12 | 67.28 | 1.32 | 5 |
| 777 | 9.26 | 1 | 3300 |  | 4 | 3 | 1 | 2 | 70.5 | 9 | 11 |  |  |  |  |  | 3 |
| 778 | 5.62 | 2 | 3200 | 50 | 3 | 3 | 1 | 1 | 65 | 7.53 | 11.5 |  |  |  |  |  | 1 |
| 779 | 27.2 | 2 | 2950 | 49 | 4 | 4 | 1 | 1 | 87 | 12.5 | 12.6 | 641.77 | 11.99 | 20.88 | 76.04 | 1.44 | 5 |
| 780 | 19.35 | 2 | 2700 | 47 | 3 | 3 | 1 | 1 | 77.3 | 9.68 | 12.4 |  |  | 8.80 | 61.13 |  | 4 |
| 781 | 34.92 | 2 | 3800 |  | 3 | 3 | 1 | 1 | 88.2 | 12.43 | 12.4 | 217.19 | 5.14 | 26.94 | 76.44 | 1.15 | 5 |
| 782 | 25.13 | 2 | 3900 |  | 3 | 3 | 1 | 1 | 90 | 11.8 | 12.8 | 542.44 | 3.22 | 14.16 | 50.06 | 1.12 | 5 |
| 783 | 1.18 | 2 | 3200 | 46 | 3 | 4 | 1 | 1 | 50 | 4.1 | 13.5 |  |  |  |  |  | 0 |
| 784 | 5.03 | 2 | 3000 |  | 3 | 4 | 1 | 1 | 70.2 | 7.625 | 12.7 |  |  |  |  |  | 2 |
| 785 | 35.81 | 1 | 2600 | 47 | 3 | 3 | 1 | 1 | 91 | 12.04 | 14.1 | 319.16 | 8.80 | 19.33 | 72.93 | 1.25 | 4 |
| 786 | 12.52 | 1 | 4200 |  | 3 | 4 | 1 | 1 | 79.5 | 11.5 | 11.4 | 373.60 | 5.83 | 4.59 | 53.86 | 1.48 | 5 |
| 787 | 35.32 | 1 | 2750 | 50 | 4 | 4 |  |  | 93.6 | 12.085 | 12.3 | 339.49 | 14.71 | 20.12 | 76.78 | 0.82 | 4 |
| 788 | 7.39 | 2 | 3500 | 48 | 3 | 2 | 2 | 2 | 65 | 6.16 | 12.4 |  |  |  |  |  | 1 |
| 789 | 20.27 | 2 | 2900 |  | 3 | 3 | 1 | 1 | 73.1 | 8.32 | 13.3 | 300.08 | 35.89 | 30.70 | 55.60 | 1.07 | 3 |
| 790 | 28.35 | 1 | 3150 | 47 | 3 | 2 | 2 | 1 | 80 | 10.3 | 12.9 | 152.00 | 10.90 | 36.35 | 65.95 | 1.13 | 1 |
| 791 | 24.94 | 1 | 3100 | 47 | 3 | 2 | 2 | 1 | 90 | 11.71 | 13.1 | 285.76 | 11.28 | 22.30 | 41.23 | 1.52 | 5 |
| 792 | 2.14 | 1 | 3150 | 48 | 4 | 3 | 1 | 1 | 59 | 5.72 | 10.6 |  |  |  |  |  | 1 |
| 793 | 33.22 | 1 | 3200 |  | 3 | 3 | 1 | 1 | 89.7 | 12.57 | 12.7 | 291.43 | 20.13 | 51.58 | 89.68 | 1.14 | 4 |
| 794 | 22.8 | 1 | 3200 | 50 | 3 | 3 | 1 | 2 | 83.3 | 10.875 | 14.4 | 408.12 | 13.79 | 13.87 | 86.38 | 0.85 | 5 |
| 795 | 2.92 | 2 | 2600 | 45 | 3 | 3 | 1 | 1 | 60 | 4.675 | 12.7 |  |  |  |  |  | 0 |
| 796 | 20.9 | 2 | 3100 | 48 | 3 | 3 | 2 | 2 | 77 | 9.7 | 13.1 | 420.69 | 3.62 | 10.93 | 53.78 | 1.08 | 3 |
| 797 | 23.66 | 1 | 2600 | 40 | 4 | 4 | 1 | 1 | 84.5 | 11.455 | 13.8 | 198.32 | 11.50 | 31.53 | 81.99 | 1.02 | 6 |
| 798 | 7.52 | 2 | 2500 |  | 3 | 2 | 1 | 1 | 68.8 | 7.165 | 12.4 |  |  |  |  |  | 2 |
| 799 | 13.44 | 1 | 2700 |  | 2 | 3 | 1 | 1 | 77.5 | 12.3 | 14.8 | 143.01 | 13.90 | 32.92 | 81.57 |  | 4 |
| 800 | 17.54 | 2 | 3600 |  | 3 | 2 | 2 | 1 | 80.5 | 11.1 | 12.9 | 175.58 | 5.86 | 6.88 | 63.89 |  | 1 |
| 801 | 3.06 | 2 | 2400 | 48 | 3 | 3 | 1 | 1 | 59 | 5.935 | 11.4 |  |  |  |  |  | 0 |
| 802 | 1.91 | 2 | 3300 | 48 | 3 | 3 | 1 | 1 | 60.5 | 5.195 | 12.6 |  |  |  |  |  | 0 |
| 803 | 28.22 | 1 | 2200 | 47 | 3 | 3 | 2 | 1 | 91.5 | 11.83 | 13.4 | 362.13 | 9.41 | 22.05 | 77.94 | 0.81 | 5 |
| 804 | 27.73 | 1 | 3300 | 48 | 3 | 3 | 2 | 1 | 86 | 11.215 | 14.4 | 242.76 | 29.91 | 25.02 | 73.12 | 0.94 | 4 |
| 805 | 12.45 | 1 |  |  | 4 | 2 | 1 | 1 | 70 | 7.8 | 12.7 |  |  |  |  |  | 3 |
| 806 | 2.76 | 2 | 2856 | 46 | 4 | 4 | 1 | 2 | 59.5 | 4.6 | 10.5 |  |  |  |  |  | 0 |
| 807 | 20.3 | 2 | 2600 |  | 3 | 3 | 1 | 1 | 81.2 | 11.05 | 13.1 | 205.30 | 7.71 | 28.89 | 69.39 | 0.91 | 4 |
| 808 | 27.6 | 2 | 2900 | 45 | 3 | 3 | 1 | 1 | 84.5 | 10.625 | 13.3 | 424.94 | 4.00 | 7.84 | 83.27 | 1.14 | 5 |
| 809 | 20.6 | 2 | 3000 |  | 3 | 3 | 1 | 1 | 80 | 9.47 | 11.3 | 56.21 | 10.56 | 8.45 | 91.16 | 0.99 | 3 |
| 810 | 18.2 | 1 | 3700 |  | 3 | 4 | 2 | 1 | 82 | 9.8 | 13.9 |  |  | 13.95 | 76.40 | 0.78 | 2 |
| 811 | 1.84 | 1 | 3350 | 48 | 3 | 3 | 1 | 1 | 55 | 5.8 | 11 |  |  |  |  |  | 1 |
| 812 | 24.31 | 2 | 2600 |  | 3 | 3 | 1 | 1 | 78.8 | 8.795 | 13 |  |  |  |  | 1.11 | 3 |
| 813 | 22.37 | 1 | 2800 |  | 3 | 3 | 1 | 1 | 78.4 | 9.785 | 12.2 | 155.54 | 12.37 | 12.97 | 82.42 | 1.21 | 3 |
| 814 | 15.21 | 1 | 3500 |  | 3 | 2 | 1 | 1 | 77.2 | 10.65 | 12.4 | 227.31 | 18.04 | 13.18 | 64.47 | 0.84 | 4 |
| 815 | 28.02 | 1 | 3300 |  | 2 | 3 | 1 | 1 | 84.6 | 10.595 | 13 | 368.31 | 16.17 | 30.66 | 55.83 | 0.97 | 4 |
| 816 | 5.29 | 1 | 2900 | 58 | 3 | 3 | 1 | 1 | 67.2 | 8.15 | 10.3 |  |  |  |  |  | 1 |
| 817 | 8.08 | 2 | 4005 |  | 5 | 5 | 1 | 1 | 70.9 | 8 | 12.2 |  |  |  |  |  | 1 |
| 818 | 28.16 | 2 | 3000 |  | 2 | 2 | 1 | 1 | 83.1 | 10.055 | 14.6 | 220.65 | 13.39 | 32.20 | 60.05 | 1.12 | 2 |
| 819 | 23.85 | 1 | 3700 |  | 3 | 2 | 1 | 1 | 90.5 | 11.48 | 12.9 | 154.94 | 15.75 | 39.79 | 58.25 | 0.86 | 2 |
| 820 | 6.51 | 1 | 3150 | 47 | 3 | 3 | 1 | 1 | 69 | 8.465 | 11.3 |  |  |  |  |  | 2 |
| 821 | 23.56 | 1 | 3000 | 48 | 3 | 3 | 1 | 1 | 82.5 | 11.35 | 14 | 243.49 | 13.45 | 14.62 | 90.17 | 1.25 | 6 |
| 822 | 29.86 | 1 | 3800 |  | 2 | 3 | 1 | 1 | 92 | 12.7 | 13 | 190.49 | 13.42 | 10.78 | 73.12 | 0.97 | 4 |
| 823 | 30.39 | 2 |  |  | 4 | 4 | 1 | 1 | 93 | 13.2 | 13.4 | 605.63 | 14.22 | 11.17 | 64.17 | 0.86 | 3 |
| 824 | 32.23 | 1 | 3650 |  | 3 | 3 | 1 | 1 | 80.5 | 11.98 | 13.9 | 261.45 | 8.02 | 12.47 | 64.47 | 1.08 | 5 |
| 825 | 11.56 | 1 | 3250 | 52 | 5 | 3 | 2 | 1 | 72 | 8.575 | 12.9 |  |  |  |  |  | 1 |
| 826 | 13.37 | 2 | 2800 |  | 3 | 3 | 1 | 1 | 75.2 | 8.5 | 11.9 | 439.57 | 9.99 | 8.52 | 131.57 | 0.89 | 1 |
| 827 | 7.92 | 2 | 2600 |  | 3 | 3 | 1 | 1 | 70.5 | 8.1 | 12.9 |  |  |  |  |  | 4 |
| 828 | 10.25 | 2 | 3000 | 45 | 3 | 4 | 1 | 2 | 71.5 | 8.665 | 10.6 |  |  |  |  |  | 3 |
| 829 | 20.4 | 2 | 2700 | 48 | 3 | 3 | 1 | 1 | 83.5 | 10.05 | 12.4 | 28.28 | 7.40 | 35.27 | 55.83 | 0.84 | 2 |
| 830 | 10.81 | 1 | 2900 |  | 3 | 3 | 1 | 1 | 72 | 7.838 | 11 |  |  |  |  |  | 6 |
| 831 | 12.71 | 1 | 3100 |  | 3 | 3 | 1 | 1 | 76 | 9.763 | 10.8 | 53.31 | 5.09 | 9.56 | 83.27 | 0.91 | 4 |
| 832 | 13.63 | 1 | 3500 |  | 4 | 3 | 1 | 1 | 76 | 9 | 11.3 | 158.21 | 15.85 | 19.76 | 88.72 | 0.94 | 5 |
| 833 | 1.64 | 1 | 2800 |  | 3 | 3 | 1 | 1 | 57 | 5.6 | 11.2 |  |  |  |  |  | 0 |
| 834 | 25.82 | 1 | 3050 | 48 | 3 | 2 | 1 | 1 | 81.5 | 10.5 | 12.4 | 154.65 | 8.10 | 21.19 | 72.07 | 1.22 | 3 |
| 835 | 17.91 | 1 | 4400 |  | 2 | 2 | 1 | 1 | 80 | 10.95 | 13.4 | 254.25 | 12.67 | 7.77 | 69.39 | 1.07 | 3 |
| 836 | 21.98 | 2 | 2550 |  | 3 | 2 | 1 | 1 | 79.9 | 7.15 | 11.9 |  |  | 28.93 | 89.20 | 1.45 | 0 |
| 837 | 9 | 2 | 3000 |  | 2 | 2 | 1 | 1 | 68.5 | 7.7 | 13.3 |  |  |  |  |  | 0 |
| 838 | 14.46 | 2 | 3500 |  | 3 | 2 | 1 | 1 | 73 | 8.83 | 13.2 |  |  | 6.64 | 65.05 | 1.00 | 3 |
| 839 | 18.73 | 2 |  |  | 2 | 2 | 1 | 1 | 76 | 9.05 | 13 |  |  | 28.78 | 67.79 | 1.11 | 4 |
| 840 | 25.66 | 1 |  |  | 2 | 2 | 2 |  | 87 | 11.88 | 13.9 | 49.76 | 3.82 | 21.29 | 61.37 | 1.22 | 4 |
| 841 | 10.84 | 1 | 3500 | 47 | 2 | 2 | 1 | 1 | 70.5 | 7.76 | 11.4 |  |  |  |  |  | 3 |
| 842 | 3.91 | 1 | 3000 |  | 3 | 3 | 1 | 1 | 60 | 6.3 | 12.2 |  |  |  |  |  | 0 |
| 843 | 1.54 | 2 | 3100 |  | 3 | 2 | 1 | 1 | 52 | 4.2 | 11.5 |  |  |  |  |  | 1 |
| 844 | 21.03 | 2 | 3500 |  | 3 | 2 | 1 | 1 | 76.5 | 8.5 | 13.4 | 164.22 | 12.47 | 25.53 | 74.55 | 1.26 | 4 |
| 845 | 8.18 | 1 | 3800 |  | 3 | 2 | 1 | 1 | 67 | 7 | 12.2 |  |  |  |  |  | 3 |
| 846 | 32.69 | 1 | 3500 |  | 3 | 3 | 2 | 1 | 85.5 | 11.82 | 12.2 | 333.21 | 3.94 | 27.04 | 55.60 | 0.96 | 3 |
| 847 | 5.19 | 2 | 3550 |  | 3 | 3 | 1 | 1 | 67 | 8.2 | 13.1 |  |  |  |  |  | 2 |
| 848 | 26.35 | 1 |  |  | 3 | 2 | 2 | 1 | 83 | 11.505 | 13.4 | 141.30 | 1.89 | 17.17 | 76.40 | 0.95 | 6 |
| 849 | 30.46 | 1 | 3050 |  | 3 | 2 | 1 | 2 | 84.8 | 10.4 | 11.3 | 555.10 | 7.08 | 21.18 | 64.47 | 1.15 | 5 |
| 850 | 31.61 | 2 | 3150 |  | 2 | 3 | 1 | 1 | 90 | 11.8 | 14.2 | 150.83 | 3.46 | 50.51 | 64.17 | 1.08 | 4 |
| 851 | 30.49 | 1 | 3050 |  | 2 | 2 | 1 | 1 | 85 | 11.1 | 12.9 |  |  | 34.34 | 55.60 | 0.99 | 3 |
| 852 | 13.27 | 2 |  |  | 3 | 2 | 1 | 1 | 78 | 10 | 13.7 | 217.19 | 12.66 | 12.64 | 64.76 | 1.36 | 5 |
| 853 | 31.64 | 1 | 2500 |  | 3 | 2 | 1 | 1 | 89.5 | 11.5 | 12.8 | 355.09 | 14.31 | 23.22 | 67.17 | 1.14 | 1 |
| 854 | 17.38 | 1 | 3700 |  | 3 | 2 | 1 | 1 | 78 | 10.1 | 12.3 |  |  | 21.24 | 72.77 | 0.89 | 3 |
| 855 | 23.56 | 1 |  |  | 3 | 2 | 2 | 1 | 80.5 | 10.6 | 12.6 |  |  | 4.79 | 62.48 | 0.89 | 1 |
| 856 | 9.43 | 2 | 3200 | 47 | 3 | 2 | 1 | 2 | 69.9 | 7.8 | 13.4 |  |  |  |  |  | 3 |
| 857 | 16.26 | 1 | 3500 |  | 2 | 2 | 1 | 1 | 82 | 11.328 | 12.4 | 220.65 | 6.95 | 6.00 | 65.65 | 1.03 | 4 |
| 858 | 9.13 | 2 | 3200 |  | 3 | 3 | 1 | 1 | 68 | 7.7 | 11.8 |  |  |  |  |  | 2 |
| 859 | 11.2 | 2 | 3000 |  | 3 | 2 | 1 | 1 | 69.5 | 6.9 | 12.6 | 369.86 | 19.08 | 16.47 | 69.49 | 0.99 | 6 |
| 860 | 13.4 | 2 | 3600 |  | 2 | 3 | 1 | 1 | 75 | 9.3 | 10.9 | 178.38 | 17.68 | 12.10 | 33.82 | 0.82 | 3 |
| 861 | 28.65 | 1 | 4500 |  | 2 | 3 | 1 | 1 | 84.5 | 10.7 | 12.4 | 194.37 | 15.23 | 17.15 | 50.94 | 0.93 | 5 |
| 862 | 19.15 | 1 | 3550 |  | 3 | 3 | 1 | 1 | 77 | 9 | 12.5 | 426.62 | 7.52 | 20.03 | 97.12 | 1.03 | 5 |
| 863 | 17.91 | 1 | 3400 |  | 3 | 3 | 1 | 1 | 73.5 | 9.45 | 11.2 | 225.88 | 10.13 | 29.87 | 74.94 | 1.23 | 2 |
| 864 | 7.59 | 2 | 3250 |  | 3 | 3 | 1 | 1 | 69.5 | 8.8 | 12.3 |  |  |  |  |  | 2 |
| 865 | 10.71 | 2 | 3000 | 48 | 3 | 3 | 1 | 1 | 70.5 | 7.9 | 13.5 |  |  |  |  |  | 1 |
| 866 | 11.17 | 1 | 2800 |  | 4 | 4 | 1 | 1 | 73.5 | 8 | 11.9 |  |  |  |  |  | 4 |
| 867 | 13.34 | 1 | 2700 |  | 4 | 3 | 1 | 1 | 76 | 10 | 11.3 | 187.47 | 8.80 | 6.98 | 75.32 | 1.04 | 4 |
| 868 | 22.08 | 2 | 2500 |  | 2 | 2 | 1 | 1 | 78.2 | 10.1 | 11.7 | 327.12 | 6.88 | 13.36 | 78.53 | 0.98 | 2 |
| 869 | 8.54 | 2 | 3000 |  | 2 | 3 | 1 | 1 | 70 | 8 | 12.9 |  |  |  |  |  | 3 |
| 870 | 6.21 | 2 | 3800 | 48 | 3 | 4 | 1 | 1 | 66.5 | 7.7 | 13.3 |  |  |  |  |  | 2 |
| 871 | 20.17 | 1 | 3800 |  | 3 | 3 | 1 | 1 | 82.5 | 11.2 | 11.4 | 310.06 | 13.37 | 4.91 | 69.15 | 1.20 | 2 |
| 872 | 20.4 | 1 | 3500 |  | 3 | 3 | 1 | 1 | 83.5 | 11.1 | 12.1 | 464.41 | 6.41 | 24.93 | 75.72 | 0.94 | 2 |
| 873 | 1.31 | 2 | 2700 | 47 | 4 | 3 | 1 | 1 | 55 | 4.8 | 12.3 |  |  |  |  |  | 0 |
| 874 | 1.97 | 1 | 3600 |  | 3 | 3 | 1 | 1 | 58.5 | 4.7 | 9.8 |  |  |  |  |  | 0 |
| 875 | 2.73 | 2 | 2500 | 47 | 3 | 3 | 1 | 1 | 58 | 5 | 12 |  |  |  |  |  | 0 |
| 876 | 3.19 | 2 | 2900 |  | 2 | 3 | 1 | 1 | 61 | 5.7 | 11.3 |  |  |  |  |  | 0 |
| 877 | 14.46 | 2 | 2650 |  | 2 | 3 | 1 | 1 | 78 | 8.3 | 12.7 | 736.42 | 11.31 | 11.13 | 94.30 | 1.23 | 3 |
| 878 | 17.05 | 2 | 3000 |  | 3 | 2 | 1 | 1 | 78.5 | 9.9 | 11.9 | 261.66 | 16.63 | 11.27 | 86.57 | 0.88 | 3 |
| 879 | 16.76 | 2 | 3700 |  | 4 | 3 | 1 | 1 | 77 | 10.2 | 11.7 | 267.22 | 10.33 | 15.48 | 82.40 | 1.04 | 2 |
| 880 | 21.03 | 1 | 2800 | 48 | 4 | 3 | 2 | 1 | 79.3 | 10.3 | 11.2 | 373.42 | 12.37 | 41.56 | 89.54 | 0.91 | 4 |
| 881 | 32.33 | 2 | 3600 |  | 3 | 4 | 1 | 1 | 83.5 | 11.4 | 12 | 295.43 | 6.83 | 26.61 | 81.08 | 1.04 | 4 |
| 882 | 16.62 | 1 | 3500 |  | 2 | 3 | 1 | 1 | 75.5 | 9.83 | 9.9 | 453.47 | 7.56 | 6.18 | 79.01 | 0.96 | 3 |
| 883 | 7.98 | 2 | 3200 |  | 2 | 2 | 1 | 1 | 70 | 8.2 | 11.8 |  |  |  |  |  | 4 |
| 884 | 11.43 | 1 | 3000 | 47 | 3 | 2 | 1 | 1 | 71.5 | 7.8 | 10.2 |  |  |  |  |  | 3 |
| 885 | 10.97 | 1 |  |  | 3 | 2 | 1 | 1 | 69 | 8 | 10.3 |  |  |  |  |  | 3 |
| 886 | 10.12 | 1 | 3300 |  | 2 | 2 | 1 | 1 | 73.5 | 8.9 | 12.1 |  |  |  |  |  | 5 |
| 887 | 12.16 | 2 |  |  | 2 | 2 | 2 | 1 | 71.5 | 7.9 | 11.6 |  |  |  |  |  | 4 |
| 888 | 13.04 | 1 | 4200 |  | 2 | 2 | 1 | 1 | 70 | 6.9 | 11.3 | 84.30 | 11.61 | 4.20 | 71.74 | 0.75 | 1 |
| 889 | 28.32 | 2 | 3800 |  | 2 | 2 | 1 | 1 | 83 | 11.2 | 12.7 | 464.86 | 6.42 | 40.79 | 99.88 | 1.06 | 2 |
| 890 | 20.6 | 1 |  |  | 1 | 1 | 1 | 1 | 79.9 | 10 | 10.1 |  |  | 4.20 | 71.00 | 0.89 | 2 |
| 891 | 8.25 | 1 | 3000 |  | 2 | 2 | 1 | 1 | 66.5 | 6.1 | 11.5 |  |  |  |  |  | 4 |
| 892 | 15.41 | 1 |  |  | 2 | 2 | 1 | 1 | 74 | 9.1 | 11.2 | 503.56 | 2.81 | 11.45 | 72.88 | 0.71 | 3 |
| 893 | 15.41 | 1 | 3000 |  | 2 | 2 | 1 | 1 | 76.5 | 8 | 11.8 | 218.28 | 14.46 | 13.12 | 49.91 | 0.99 | 5 |
| 894 | 1.64 | 2 | 2700 | 56 | 2 | 2 | 1 | 1 | 56.5 | 5 | 11.7 |  |  |  |  |  | 0 |
| 895 | 14.36 | 2 | 3000 |  | 2 | 2 | 1 | 1 | 73.5 | 8.3 | 11.7 |  | 0.30 | 12.92 | 77.72 | 0.80 | 3 |
| 896 | 6.01 | 1 | 3300 | 51 | 2 | 3 | 2 | 1 | 66.5 | 8.05 | 12.3 |  |  |  |  |  | 4 |
| 897 | 2.07 | 2 | 3150 |  | 2 | 3 | 1 | 1 | 59.5 | 6 | 12 |  |  |  |  |  | 0 |
| 898 | 14.29 | 2 | 2900 |  | 2 | 1 | 1 | 1 | 70 | 6.8 | 11.3 |  |  | 16.57 | 96.23 | 0.93 | 3 |
| 899 | 24.8 | 2 | 2700 |  | 2 | 2 | 1 | 1 | 79 | 8.3 | 11.4 | 371.19 | 7.11 | 47.26 | 76.06 | 1.12 | 5 |
| 900 | 15.21 | 2 | 3200 |  | 3 | 3 | 1 | 1 | 72.5 | 7.1 | 11.9 | 368.53 | 5.06 | 11.46 | 80.33 | 1.01 | 4 |
| 901 | 11.73 | 2 | 2500 |  | 2 | 3 | 1 | 1 | 69.7 | 6.8 | 11.8 | 191.41 | 11.01 | 12.18 | 81.23 | 0.91 | 6 |
| 902 | 10.97 | 1 | 3250 |  | 3 | 3 | 1 | 1 | 72 | 7.85 | 10.2 |  |  |  |  |  | 6 |
| 903 | 12.45 | 2 | 3400 |  | 2 | 3 | 1 | 1 | 73 | 7.82 | 11.9 | 171.00 | 39.82 | 40.32 | 114.52 | 0.83 | 1 |
| 904 | 3.88 | 1 | 3300 |  | 2 | 2 | 1 | 1 | 65.5 | 6.5 | 13.4 |  |  |  |  |  | 0 |
| 905 | 3.68 | 2 | 3350 |  | 4 | 3 | 1 | 2 | 63 | 6.05 | 12.7 |  |  |  |  |  | 1 |
| 906 | 12.22 | 1 | 3100 |  | 4 | 3 | 1 | 1 | 75.5 | 9.7 | 14.4 | 716.03 | 15.78 | 27.10 | 77.72 | 1.08 | 5 |
| 907 | 21.13 | 1 | 3250 |  | 3 | 2 | 2 | 1 | 84.5 | 11.79 | 11.8 | 297.18 | 14.64 | 11.75 | 106.41 | 1.05 | 4 |
| 908 | 5.26 | 1 | 3700 | 45 | 3 | 2 | 1 | 1 | 66 | 6.8 | 10.6 |  |  |  |  |  | 1 |
| 909 | 1.02 | 2 | 3150 | 47 | 3 | 3 | 1 | 1 | 53 | 3.5 | 10.5 |  |  |  |  |  | 0 |
| 910 | 12.55 | 2 | 3000 | 47 | 3 | 3 | 1 | 1 | 70.5 | 6.7 | 12.3 | 124.07 | 17.20 | 7.18 | 114.52 | 0.90 | 3 |
| 911 | 24.8 | 2 | 3200 |  | 3 | 3 | 1 | 1 | 83.5 | 9.875 | 14 | 242.79 | 6.55 | 22.15 | 59.94 | 1.36 | 5 |
| 912 | 7.39 | 2 | 3700 |  | 5 | 4 | 1 | 2 | 67.5 | 7.8 | 11.5 |  |  |  |  |  | 3 |
| 913 | 29.24 | 1 | 2300 |  | 2 | 2 | 2 | 2 | 84 | 11.575 | 12 |  |  | 9.19 | 54.83 | 1.01 | 3 |
| 914 | 23.46 | 2 | 2150 | 48 | 2 | 2 | 1 | 1 | 79 | 8.9 | 12.4 | 479.15 | 4.85 | 13.87 | 33.20 | 1.26 | 4 |
| 915 | 1.58 | 1 | 3000 | 46 | 2 | 2 | 1 | 1 | 54 | 5.4 | 13 |  |  |  |  |  | 0 |
| 916 | 27.56 | 2 | 3500 | 48 | 3 | 3 | 2 | 1 | 81 | 12.2 | 13.6 | 308.06 | 13.46 | 9.00 | 58.80 | 1.17 | 5 |
| 917 | 14.46 | 1 | 2550 |  | 3 | 3 | 2 | 1 | 74.5 | 8.1 | 10.6 | 451.04 | 8.25 | 13.49 | 72.12 | 1.07 | 3 |
| 918 | 21.45 | 1 | 3800 | 49 | 3 | 3 | 1 | 2 | 82.5 | 10.9 | 13 | 450.02 | 10.45 | 10.46 | 70.27 | 1.02 | 6 |
| 919 | 4.01 | 2 | 3500 |  | 3 | 3 | 1 | 1 | 64.5 | 7 | 13 |  |  |  |  |  | 0 |
| 920 | 10.74 | 2 | 2800 |  | 3 | 3 | 1 | 1 | 68.5 | 7.52 | 13.5 |  |  |  |  |  | 3 |
| 921 | 12.65 | 2 | 3400 |  | 3 | 3 | 1 | 1 | 68.5 | 8.1 | 12.6 |  |  |  |  |  | 4 |
| 922 | 24.64 | 2 | 2300 | 46 | 3 | 2 | 1 | 1 | 85 | 9.8 | 12.7 | 233.92 | 13.85 | 13.49 | 53.11 | 1.14 | 3 |
| 923 | 14.46 | 2 | 3600 |  | 3 | 3 | 1 | 1 | 71 | 8.9 | 12.3 | 121.38 | 19.60 | 17.76 | 59.08 | 1.10 | 1 |
| 924 | 21.09 | 2 | 2800 | 46 | 3 | 3 | 1 | 1 | 82 | 9 | 13.3 | 170.05 | 9.57 | 8.11 | 65.87 | 1.14 | 6 |
| 925 | 32.99 | 1 | 2750 | 46 | 4 | 3 | 1 | 1 | 92.5 | 13.42 | 13 |  | 20.07 | 21.66 | 94.49 | 0.83 | 5 |
| 926 | 21.13 | 1 | 2800 |  | 3 | 4 | 1 | 1 | 83 | 12.55 | 12.4 |  |  | 7.56 | 37.49 |  | 6 |
| 927 | 14.19 | 2 | 3500 |  | 3 | 2 | 2 | 2 | 75 | 8.1 | 11.8 | 264.62 | 10.31 | 6.19 | 44.64 | 1.15 | 4 |
| 928 | 23.89 | 1 | 2500 |  | 3 | 3 | 1 | 1 | 76 | 9.7 | 12.5 |  |  | 12.00 | 77.67 | 1.12 | 3 |
| 929 | 28.39 | 2 | 2800 |  | 3 | 3 | 1 | 1 | 84.5 | 10.7 | 13.2 |  |  | 11.67 | 51.57 | 1.26 | 3 |
| 930 | 6.7 | 1 | 3100 | 46 | 4 | 3 | 1 | 2 | 71 | 8.08 | 12.1 |  |  |  |  |  | 3 |
| 931 | 18.46 | 1 | 3400 |  | 4 | 3 | 1 | 1 | 78.2 | 9.25 | 11.8 | 434.65 | 9.11 | 7.18 | 56.29 | 1.01 | 4 |
| 932 | 7.39 | 1 | 3400 | 48 | 4 | 3 | 1 | 1 | 72 | 8.77 | 12.5 |  |  |  |  |  | 3 |
| 933 | 21.91 | 1 | 3500 | 48 | 3 | 2 | 2 | 2 | 86 | 10.725 | 12.6 |  |  |  |  | 1.14 | 1 |
| 934 | 32.26 | 2 | 2800 | 46 | 5 | 4 | 1 | 1 | 85 | 11.87 | 12.4 | 392.32 | 11.49 | 67.86 | 100.07 | 1.32 | 4 |
| 935 | 2.23 | 1 | 3000 |  | 2 | 2 | 1 | 1 | 55 | 5.3 | 11 |  |  |  |  |  | 0 |
| 936 | 11.14 | 2 | 2900 |  | 3 | 3 | 1 | 1 | 71 | 9.225 | 11 |  |  |  |  |  | 3 |
| 937 | 28.88 | 1 | 3000 |  | 3 | 2 | 1 | 1 | 86 | 13 | 12.4 | 365.45 | 10.43 | 9.99 | 59.18 | 1.26 | 3 |
| 938 | 30.49 | 1 | 2900 |  | 2 | 3 | 1 | 2 | 88.2 | 11.46 | 12.6 | 279.36 | 12.12 | 36.52 | 66.30 | 1.22 | 4 |
| 939 | 14.29 | 1 | 3000 |  | 3 | 3 | 1 | 1 | 74 | 7.9 | 12.8 | 128.95 | 19.73 | 18.19 | 67.62 | 0.95 | 3 |
| 940 | 6.14 | 2 | 3200 |  | 3 | 3 | 1 | 1 | 64 | 7 | 11.8 |  |  |  |  |  | 2 |
| 941 | 15.57 | 1 | 3700 |  | 3 | 3 | 1 | 1 | 83 | 12 | 13 | 434.10 | 58.19 | 17.11 | 122.13 | 1.35 | 5 |
| 942 | 7.29 | 1 | 3450 |  | 3 | 2 | 2 | 2 | 72 | 8.93 | 10.9 |  |  |  |  |  | 4 |
| 943 | 15.15 | 1 | 3000 |  | 3 | 3 | 1 | 1 | 76.5 | 9 | 11.3 | 185.99 | 14.17 | 10.38 | 65.35 | 1.05 | 4 |
| 944 | 4.44 | 1 | 2700 |  | 3 | 2 | 1 | 1 | 63 | 7.325 | 11.6 |  |  |  |  |  | 0 |
| 945 | 11.86 | 2 | 3150 |  | 3 | 3 | 1 | 1 | 69 | 7.87 | 12.4 |  |  |  |  |  | 3 |
| 946 | 28.78 | 2 | 4200 | 53 | 3 | 3 | 1 | 1 | 89.2 | 13.16 | 13.3 | 325.26 | 15.85 | 19.34 | 75.24 | 0.98 | 4 |
| 947 | 20.14 | 2 | 2600 | 47 | 4 | 2 | 1 | 1 | 75.5 | 8.8 | 13 |  |  |  |  | 1.02 | 2 |
| 948 | 25.89 | 1 | 3800 |  | 3 | 4 | 1 | 1 | 84 | 11.65 | 12.9 | 219.96 | 4.03 | 21.96 | 62.29 | 1.30 | 5 |
| 949 | 11.6 | 1 | 2900 |  | 3 | 3 | 2 | 1 | 71.5 | 8.5 | 12.4 |  |  |  |  |  | 3 |
| 950 | 28.78 | 2 | 3100 |  | 3 | 3 | 1 | 1 | 84.5 | 10.54 | 12.8 |  | 6.44 | 12.72 | 80.23 | 1.13 | 3 |
| 951 | 16.99 | 1 | 3000 | 49 | 3 | 3 | 1 | 1 | 73.5 | 8.6 | 13.2 | 307.20 | 5.68 | 9.25 | 70.02 | 1.08 | 5 |
| 952 | 15.05 | 1 | 3600 | 48.3 | 3 | 3 | 1 | 2 | 76.5 | 9.25 | 12.2 | 602.35 | 5.99 | 6.34 | 61.40 | 0.86 | 3 |
| 953 | 9.95 | 1 | 3650 | 50 | 3 | 3 | 2 | 1 | 74.3 | 9.75 | 7.7 |  |  |  |  |  | 1 |
| 954 | 32.43 | 2 | 3350 | 50 | 3 | 3 | 1 | 1 | 85.5 | 12.21 | 10.9 |  |  |  |  |  | 4 |
| 955 | 5.06 | 1 | 3550 | 50 | 3 | 3 | 1 | 2 | 65 | 8 | 9.8 |  |  |  |  |  | 1 |
| 956 | 7.85 | 2 | 3300 | 50 | 2 | 2 | 1 | 1 | 67 | 7.98 | 12.5 |  |  |  |  |  | 3 |
| 957 | 28.06 | 2 | 3000 | 45 | 3 | 3 | 1 | 1 | 88.5 | 12.15 | 10.4 | 394.42 | 5.33 | 22.06 | 47.17 | 1.08 | 4 |
| 958 | 29.01 | 2 | 3500 | 45 | 3 | 3 | 1 | 1 | 84.3 | 11.01 | 11.6 | 593.03 | 8.80 | 27.18 | 61.40 | 1.12 | 4 |
| 959 | 22.28 | 2 | 3600 | 48 | 2 | 3 | 1 | 1 | 86.5 | 11 | 12.7 |  |  | 16.81 | 58.44 | 1.03 | 3 |
| 960 | 2.17 | 1 | 3400 | 48 | 3 | 3 | 1 | 1 | 62 | 6.4 | 10.4 |  | 9.94 | 6.74 | 80.06 | 1.13 | 0 |
| 961 | 7.43 | 2 | 3800 | 55 | 3 | 3 | 1 | 1 | 70 | 7.25 | 11.5 |  |  |  |  |  | 2 |
| 962 | 21.55 | 2 | 3300 | 50 | 4 | 3 | 1 | 1 | 84.4 | 11.28 | 13.6 | 356.48 | 4.73 |  |  |  | 3 |
| 963 | 32.1 | 2 | 2500 | 48 | 3 | 3 | 1 | 1 | 90.3 | 12.23 | 13.3 | 539.35 | 5.03 | 18.07 | 80.49 | 1.14 | 5 |
| 964 | 20.3 | 2 | 3700 | 50 | 3 | 3 | 1 | 1 | 82.3 | 10.83 | 11.6 | 498.77 | 4.11 | 15.81 | 81.79 | 1.06 | 3 |
| 965 | 16.46 | 2 | 3350 | 50 | 4 | 3 | 1 | 1 | 83 | 11.21 | 14.4 | 568.14 | 2.35 |  |  | 1.20 | 1 |
| 966 | 18.6 | 1 | 3250 | 50 | 3 | 2 | 1 | 1 | 92 | 11.92 | 12.8 |  |  |  |  |  | 2 |
| 967 | 33.31 | 2 | 3200 | 48 | 3 | 2 | 1 | 1 | 82.5 | 13.41 | 13.3 |  | 72.96 | 37.28 | 59.49 | 1.26 | 2 |
| 968 | 25.49 | 2 | 3350 | 49 | 3 | 3 | 1 | 1 | 90 | 12.34 | 12.8 | 480.19 | 7.62 | 20.78 | 81.35 | 1.08 | 4 |
| 969 | 30.78 | 1 | 3450 | 50 | 4 | 4 | 1 | 1 | 90 | 13.25 | 11.9 |  | 72.82 | 22.44 | 60.57 | 1.14 | 4 |
| 970 | 15.31 | 1 | 3400 | 50 | 3 | 3 | 1 | 1 | 77.5 | 10.57 | 12.4 | 256.66 | 4.89 | 5.80 | 78.80 | 0.78 | 5 |
| 971 | 25.23 | 1 | 3150 | 50 | 2 | 4 | 1 | 1 | 85 | 12.46 | 12.8 | 689.90 | 14.10 | 34.09 | 94.72 | 1.28 | 5 |
| 972 | 16.79 | 2 | 3900 | 50 | 3 | 3 | 1 | 1 | 83.2 | 11.74 | 13.5 |  | 8.13 | 9.58 | 87.38 | 1.17 | 3 |
| 973 | 10.51 | 2 | 3200 | 48 | 4 | 5 | 1 | 1 | 71 | 9.43 | 10.2 |  |  |  |  |  | 3 |
| 974 | 25.86 | 2 | 3600 | 52 | 3 | 3 | 1 | 1 | 90 | 12.63 | 8.3 |  |  |  |  |  | 4 |
| 975 | 12.71 | 1 | 4100 | 53 | 2 | 2 | 1 | 1 | 79.7 | 10.96 | 10.1 |  |  |  |  |  | 3 |
| 976 | 6.05 | 1 | 2800 | 50 | 3 | 3 | 1 | 1 | 64.5 | 8.57 | 10.9 |  |  |  |  |  | 1 |
| 977 | 31.87 | 1 | 3150 | 50 | 3 | 3 | 1 | 1 | 90.2 | 11.76 | 11.9 |  | 75.99 | 24.73 | 60.02 | 1.10 | 4 |
| 978 | 14.13 | 2 | 3300 | 50 | 3 | 3 | 1 | 1 | 75.5 | 9.25 | 12 |  |  |  |  |  | 3 |
| 979 | 25.07 | 2 |  | 48 | 3 | 3 | 1 | 1 | 83.5 | 10.81 | 12.4 |  | 77.18 | 20.09 | 50.54 | 1.10 | 4 |
| 980 | 33.64 | 2 | 3350 | 50 | 3 | 1 | 1 | 1 | 91.5 | 11.8 | 11.2 |  |  | 26.29 | 67.75 | 1.16 | 3 |
| 981 | 9.23 | 2 | 3650 | 45 | 3 | 1 | 1 | 1 | 69 | 7.335 | 9.5 |  |  |  |  |  | 1 |
| 982 | 23.1 | 2 | 2900 |  | 3 | 2 | 1 | 1 | 78.2 | 9.07 | 12 | 301.46 | 2.10 | 17.24 | 50.74 | 0.95 | 4 |
| 983 | 19.98 | 2 | 3000 | 48 | 5 | 4 | 1 | 1 | 82.3 | 11.66 | 11.9 |  |  |  |  |  | 5 |
| 984 | 9.66 | 2 | 3200 | 50 | 4 | 5 | 1 | 2 | 66.5 | 7 | 11.7 |  |  |  |  |  | 2 |
| 985 | 5.52 | 1 | 3700 | 50 | 3 | 3 | 1 | 2 | 67.4 | 8.375 | 11.2 |  |  |  |  |  | 1 |
| 986 | 11.01 | 1 | 2700 |  | 4 | 3 | 1 | 2 | 72.6 | 8.973 | 10.4 |  |  |  |  |  | 2 |
| 987 | 33.91 | 1 | 3200 | 50 | 3 | 2 | 1 | 1 | 97.8 | 15.94 | 12.4 | 224.73 | 4.29 | 34.49 | 60.57 | 0.91 | 2 |
| 988 | 5.16 | 1 | 3600 | 55 | 3 | 4 | 1 | 1 | 68.2 | 7.21 | 10.8 |  |  |  |  |  | 1 |
| 989 | 4.44 | 1 | 1900 | 50 | 2 | 2 | 1 | 1 | 68 | 6.74 | 10.6 |  |  |  |  |  | 1 |
| 990 | 6.05 | 1 | 2550 | 50 | 2 | 2 | 1 | 1 | 65.1 | 8.44 | 12.1 |  |  |  |  |  | 0 |
| 991 | 8.25 | 2 | 4150 | 50 | 3 | 2 | 1 | 1 | 70 | 9.76 | 12.2 |  |  |  |  |  | 1 |
| 992 | 2.83 | 2 | 3650 | 50 | 2 | 2 | 1 | 1 | 60.1 | 6.59 | 11.8 |  |  |  |  |  | 0 |
| 993 | 34.37 | 1 | 3250 | 50 | 2 | 2 | 1 | 1 | 96.1 | 13.92 | 12.2 | 524.80 | 3.59 | 26.54 | 82.68 | 1.14 | 4 |
| 994 | 11.4 | 1 | 4200 | 52 | 3 | 3 | 1 | 1 | 76.1 | 9.43 | 11.9 |  |  |  |  |  | 5 |
| 995 | 5.68 | 2 | 3750 | 50 | 3 | 3 | 1 | 1 | 67.1 | 7.7 | 9.4 |  |  |  |  |  | 0 |
| 996 | 19.32 | 1 | 3350 | 50 | 4 | 3 | 1 | 1 | 83.1 | 11.91 | 12.5 | 321.23 | 4.61 | 11.65 | 68.74 |  | 4 |
| 997 | 14 | 2 | 2350 | 50 | 3 | 3 | 1 | 1 | 78 | 10.09 | 10.4 |  | 78.45 | 0.00 | 50.96 | 1.01 | 4 |
| 998 | 30.85 | 2 | 3300 | 50 | 3 | 3 | 1 | 1 | 84.2 | 10.37 | 12.2 |  | 5.29 | 2.26 | 63.41 | 1.09 | 4 |
| 999 | 18.96 | 1 | 2950 | 51 | 3 | 3 | 1 | 1 | 79.1 | 9.66 | 12.7 |  | 3.12 | 22.00 | 72.79 | 1.21 | 7 |
| 1000 | 5.13 | 2 | 3000 | 50 | 4 | 3 | 1 | 1 | 63.9 | 7.41 | 10.9 |  |  |  |  |  | 0 |
| 1001 | 28.45 | 2 | 2850 | 50 | 3 | 3 | 1 | 1 | 89.3 | 13.3 | 12.3 | 764.71 | 5.89 | 23.68 | 77.16 | 1.13 | 4 |
| 1002 | 24.08 | 1 | 3750 | 50 | 3 | 3 | 1 | 1 | 86.3 | 12.99 | 13.2 |  | 7.40 | 12.82 | 86.40 | 1.01 | 3 |
| 1003 | 11.53 | 2 | 3500 | 50 | 4 | 3 | 1 | 1 | 72.6 | 9.72 | 11.9 |  |  |  |  |  | 6 |
| 1004 | 4.8 | 2 | 3400 | 50 | 3 | 3 | 2 | 1 | 65.3 | 7.07 | 12.9 |  |  |  |  |  | 0 |
| 1005 | 3.98 | 1 | 3700 | 55 | 3 | 3 | 2 | 1 | 66.5 | 7.74 | 11.1 |  |  |  |  |  | 0 |
| 1006 | 34.53 | 2 | 2750 | 50 | 3 | 3 | 1 | 1 | 91.3 | 12.841 | 13.3 |  |  | 31.99 | 55.68 | 1.06 | 5 |
| 1007 | 32.76 | 1 | 3450 | 51 | 3 | 2 | 1 | 1 | 92.3 | 12.47 | 13.4 | 301.02 | 3.30 | 28.04 | 54.50 | 1.26 | 3 |
| 1008 | 3.68 | 2 | 3600 | 55 | 2 | 3 | 1 | 1 | 60 | 6.57 | 11.8 |  |  |  |  |  | 0 |
| 1009 | 17.81 | 1 | 3100 | 50 | 4 | 4 | 2 | 1 | 83.1 | 11.39 | 12.5 |  | 6.93 | 14.89 | 98.19 | 0.95 | 4 |
| 1010 | 25.56 | 2 | 3400 | 50 | 3 | 2 | 1 | 1 | 89.5 | 12.43 | 12.5 |  |  |  |  |  | 4 |
| 1011 | 34.17 | 2 | 3100 | 50 | 3 | 3 | 1 | 1 | 96.4 | 13.65 | 12.4 |  |  | 36.10 | 57.67 | 1.29 | 4 |
| 1012 | 15.11 | 1 | 3300 | 50 | 5 | 4 | 1 | 1 | 79.5 | 9.89 | 12.6 | 502.69 | 5.68 | 13.38 | 57.16 | 1.15 | 6 |
| 1013 | 15.01 | 2 | 3100 | 49 | 3 | 1 | 1 | 2 | 79.8 | 10.02 | 12.5 | 665.07 | 4.45 | 3.21 | 64.91 | 1.03 | 3 |
| 1014 | 7.33 | 1 | 2700 | 49 | 6 | 4 | 2 | 2 | 68.5 | 7.75 | 9.8 |  |  |  |  |  | 0 |
| 1015 | 33.87 | 2 | 3150 | 50 | 3 | 3 | 2 | 2 | 88.6 | 10.97 | 12.5 |  |  |  |  |  | 2 |
| 1016 | 8.38 | 1 | 3900 | 55 | 4 | 4 | 2 | 1 | 68.1 | 10.19 | 6 |  |  |  |  |  | 1 |
| 1017 | 30.69 | 1 | 3450 | 50 | 4 | 3 | 1 | 1 | 97.6 | 13.47 | 11.3 | 568.87 | 4.68 | 44.60 | 65.83 | 1.12 | 5 |
| 1018 | 7.82 | 1 | 3000 | 50 | 5 | 5 | 1 | 1 | 72.1 | 9.33 | 8.2 |  |  |  |  |  | 7 |
| 1019 | 9.13 | 2 | 2100 | 48 | 5 | 4 | 2 | 1 | 69.1 | 7.58 | 11.6 |  |  |  |  |  | 7 |
| 1020 | 9.13 | 2 | 2100 | 50 | 5 | 4 | 2 | 1 | 70 | 7.56 | 10.7 |  |  |  |  |  | 7 |
| 1021 | 27.33 | 1 | 4000 | 53 | 3 | 3 | 2 | 1 | 92.2 | 13.08 | 11.4 | 863.05 | 26.41 | 39.11 | 64.91 | 1.31 | 5 |
| 1022 | 9.26 | 1 | 3850 | 50 | 5 | 5 | 1 | 1 | 72.6 | 10.1 | 12.7 |  |  |  |  |  | 2 |
| 1023 | 5.36 | 1 | 3205 | 50 | 5 | 6 | 1 | 1 | 68.7 | 7.2 | 8.9 |  |  |  |  |  | 0 |
| 1024 | 5.72 | 1 | 3400 | 50 | 6 | 4 | 1 | 1 | 69 | 7.75 | 11.8 |  |  |  |  |  | 0 |
| 1025 | 29.96 | 2 | 2800 | 48 | 3 | 2 | 1 | 1 | 87.4 | 11.89 | 13.4 | 444.87 | 3.76 | 16.07 | 58.18 | 1.46 | 3 |
| 1026 | 29.9 | 1 | 3200 | 50 | 3 | 3 | 1 | 1 | 91.8 | 13.12 | 12.7 | 259.06 | 3.87 | 47.10 | 52.68 | 0.95 | 5 |
| 1027 | 12.06 | 1 | 3150 | 50 | 4 | 4 | 1 | 2 | 72 | 8.51 | 10.7 | 171.99 | 3.46 | 23.90 | 118.38 |  | 7 |
| 1028 | 35.06 | 2 | 2950 | 48 | 4 | 5 | 1 | 1 | 95.5 | 14.91 | 12.8 | 728.14 | 4.43 | 14.50 | 60.57 | 1.09 | 7 |
| 1029 | 8.11 | 2 | 3550 | 55 | 4 | 5 | 1 | 2 | 72.2 | 8.44 | 10.7 |  |  |  |  |  | 7 |
| 1030 | 28.25 | 2 | 3250 | 50 | 5 | 3 | 1 | 1 | 79.7 | 9.25 | 12.1 | 774.96 | 3.93 | 10.80 | 71.14 | 1.29 | 4 |
| 1031 | 4.93 | 1 | 3950 | 50 | 4 | 3 | 1 | 2 | 66 | 7.06 | 12.1 |  |  |  |  |  | 0 |
| 1032 | 14.52 | 1 | 4350 | 50 | 3 | 2 | 1 | 1 | 80.1 | 9.81 | 13.7 |  |  | 42.68 | 62.82 |  | 4 |
| 1033 | 1.54 | 1 | 3550 | 50 | 6 | 3 | 1 | 1 | 58.7 | 4.56 | 9.4 |  |  |  |  |  | 0 |
| 1034 | 13.9 | 1 | 3210 | 48 | 3 | 3 | 1 | 1 | 74.1 | 9.76 | 11.6 | 369.49 | 7.97 | 14.89 | 88.87 | 1.00 | 2 |
| 1035 | 8.8 | 1 | 3200 | 50 | 3 | 3 | 1 | 1 | 72.1 | 9.77 | 10.6 |  |  |  |  |  | 3 |
| 1036 | 21.03 | 2 | 4000 | 50 | 3 | 4 | 1 | 1 | 87.5 | 12.9 | 11 | 665.07 | 8.63 | 25.01 | 64.30 | 1.06 | 5 |
| 1037 | 2.86 | 2 | 3150 | 52 | 3 | 2 | 1 | 1 | 56 | 4.46 | 9.6 |  |  |  |  |  | 1 |
| 1038 | 25 | 1 | 3050 | 49 | 4 | 4 | 1 | 1 | 78 | 10.77 | 13.2 |  |  | 14.84 | 80.92 | 1.40 | 4 |
| 1039 | 11.53 | 1 | 3100 | 50 | 4 | 3 | 2 | 1 | 76.5 | 9.49 | 10.1 |  |  |  |  |  | 3 |
| 1040 | 2.04 | 1 | 2950 | 50 | 3 | 3 | 1 | 2 | 56.7 | 5.16 | 6.8 |  |  |  |  |  | 0 |
| 1041 | 29.34 | 2 | 3000 | 50 | 2 | 1 | 1 | 1 | 81 | 11.64 | 12.8 | 135.12 | 4.00 | 32.55 | 63.11 | 0.93 | 5 |
| 1042 | 2.2 | 1 | 2650 | 50 | 2 | 3 | 1 | 1 | 61.7 | 5.4 | 8.7 |  |  |  |  |  | 0 |
| 1043 | 18.37 | 1 | 3450 | 51 | 4 | 3 | 1 | 1 | 79.8 | 10.78 | 12.9 | 748.64 | 9.87 | 18.80 | 60.85 | 1.20 | 5 |
| 1044 | 4.27 | 1 | 2550 | 50 | 4 | 4 | 2 | 2 | 62.5 | 6.34 | 9.9 |  |  |  |  |  | 1 |
| 1045 | 26.74 | 1 | 3150 | 50 | 4 | 4 | 1 | 1 | 88 | 13 | 11.8 | 337.09 | 4.82 | 21.24 | 57.16 | 0.82 | 7 |
| 1046 | 7.69 | 1 | 3200 | 50 | 3 | 3 | 2 | 1 | 74 | 9.38 | 12.3 |  |  |  |  |  | 2 |
| 1047 | 3.52 | 1 | 3850 | 50 | 3 | 3 | 1 | 2 | 64 | 7.4 | 11.6 |  |  |  |  |  | 0 |
| 1048 | 25.59 | 2 | 3400 | 50 | 3 | 3 | 1 | 1 | 77 | 9.48 | 12.1 |  |  | 11.87 | 84.05 | 1.38 | 4 |
| 1049 | 15.08 | 1 | 2800 | 50 | 3 | 3 | 2 | 1 | 78.8 | 10.57 | 11 |  |  |  |  |  | 7 |
| 1050 | 7.52 | 2 | 3400 | 50 | 4 | 4 | 1 | 1 | 69.9 | 9.59 | 7.7 |  |  |  |  |  | 1 |
| 1051 | 21.88 | 2 | 3550 | 49 | 3 | 3 | 1 | 1 | 86.2 | 11.96 | 13.5 | 738.80 | 7.64 | 12.93 | 71.50 | 1.05 | 5 |
| 1052 | 32.69 | 2 | 2950 | 50 | 6 | 3 | 1 | 1 | 90.2 | 12.35 | 12.4 |  | 7.50 | 28.74 | 68.74 | 1.06 | 7 |
| 1053 | 32.59 | 1 | 3100 | 48 | 6 | 5 | 1 | 1 | 87 | 10.91 | 12.2 | 596.90 | 5.29 | 46.81 | 59.49 | 1.10 | 6 |
| 1054 | 22.24 | 1 | 2800 | 50 | 5 | 5 | 1 | 1 | 84.4 | 11.8 | 13.2 | 572.58 | 2.66 | 12.12 | 70.10 | 1.24 | 6 |
| 1055 | 29.9 | 2 | 3800 | 52 | 3 | 3 | 1 | 1 | 83.2 | 12.66 | 7.9 |  |  |  |  |  | 6 |
| 1056 | 25.23 | 1 | 3200 | 50 | 2 | 3 | 1 | 1 | 87 | 13.15 | 12 |  |  |  |  |  | 5 |
| 1057 | 25.66 | 1 | 3500 | 50 | 3 | 3 | 2 | 1 | 85.4 | 11.62 | 12 | 279.56 | 7.26 | 18.39 | 70.79 | 1.13 | 4 |
| 1058 | 20.44 | 2 | 3950 | 50 | 3 | 3 | 1 | 1 | 83 | 12.26 | 12.8 | 413.45 | 6.14 | 39.46 | 58.96 | 0.84 | 5 |
| 1059 | 28.16 | 1 | 3350 | 50 | 6 | 4 | 1 | 1 | 85.3 | 10.88 | 12 | 272.49 | 2.83 | 11.61 | 51.59 | 1.08 | 5 |
| 1060 | 7.46 | 2 | 2250 | 50 | 3 | 3 | 2 | 2 | 63.1 | 6.82 | 11.2 |  |  |  |  |  | 2 |
| 1061 | 32.36 | 1 | 3850 | 50 | 3 | 3 | 1 | 1 | 89.5 | 14.27 | 12.5 | 390.17 | 3.65 | 21.07 | 60.02 | 0.99 | 5 |
| 1062 | 29.7 | 2 | 2900 | 49 | 2 | 2 | 1 | 1 | 80 | 10.2 | 12.2 |  | 6.31 | 26.02 | 77.16 | 1.14 | 5 |
| 1063 | 20.63 | 1 | 3600 | 52 | 3 | 1 | 2 | 1 | 83 | 11.59 | 12.5 | 314.39 | 4.01 | 15.20 | 71.50 | 1.15 | 3 |
| 1064 | 13.96 | 2 | 3000 | 50 | 2 | 2 | 1 | 1 | 79.6 | 8.81 | 12.7 |  |  |  |  |  | 6 |
| 1065 | 7.13 | 1 | 3250 | 50 | 2 | 3 | 2 | 1 | 74.2 | 11.03 | 11.1 |  |  |  |  |  | 1 |
| 1066 | 22.97 | 2 | 3350 | 49 | 2 | 4 | 2 | 1 | 90.2 | 13.99 | 12.3 |  |  | 87.12 | 71.14 | 1.47 | 4 |
| 1067 | 12.52 | 1 | 3500 | 48 | 3 | 3 | 2 | 2 | 74.7 | 10.37 | 11.4 |  |  |  |  |  | 2 |
| 1068 | 10.35 | 2 | 2500 | 48 | 2 | 2 | 1 | 1 | 70.1 | 6.905 | 13.5 |  |  |  |  |  | 3 |
| 1069 | 6.51 | 1 | 3350 | 48 | 6 | 6 | 2 | 2 | 67.5 | 8.79 | 10.9 |  |  |  |  |  | 2 |
| 1070 | 20.3 | 2 | 3000 | 50 | 5 | 5 | 1 | 1 | 81 | 10.27 | 12 | 535.85 | 4.17 | 21.98 | 61.96 | 1.01 | 4 |
| 1071 | 8.18 | 1 | 3200 | 50 | 6 | 4 | 1 | 2 | 71 | 8.33 | 11.2 |  |  |  |  |  | 1 |
| 1072 | 8.8 | 2 | 3500 | 50 | 3 | 3 | 1 | 1 | 65.5 | 7.315 | 12.1 |  |  |  |  |  | 3 |
| 1073 | 7.92 | 1 | 3100 | 50 | 3 | 2 | 1 | 1 | 74 | 11.27 | 10.3 |  |  |  |  |  | 0 |
| 1074 | 12.19 | 2 | 2800 | 50 | 2 | 3 | 2 | 1 | 72.6 | 7.97 | 8.7 |  |  |  |  |  | 3 |
| 1075 | 34.66 | 2 | 3000 | 48 | 2 | 3 | 1 | 1 | 91 | 17.274 | 12.7 | 291.89 | 2.89 | 20.12 | 57.50 | 0.93 | 4 |
| 1076 | 31.57 | 1 | 3500 | 50 | 2 | 3 | 1 | 1 | 90.6 | 12.44 | 12.7 | 329.96 | 1.72 | 17.95 | 65.35 | 0.96 | 4 |
| 1077 | 15.05 | 1 | 2900 | 50 | 4 | 3 | 2 | 1 | 84.1 | 10.67 | 12.7 | 495.63 | 9.85 | 33.76 | 82.05 | 1.10 | 5 |
| 1078 | 20.7 | 1 | 3100 | 50 | 3 | 3 | 1 | 1 | 84.2 | 11.74 | 12.8 | 568.89 | 5.07 | 35.35 | 83.08 | 1.21 | 6 |
| 1079 | 20.04 | 1 | 3100 | 50 | 5 | 4 | 2 | 1 | 85.1 | 11.62 | 12 | 677.44 | 3.45 | 25.69 | 73.19 | 1.50 | 6 |
| 1080 | 7.79 | 1 | 3200 | 50 | 3 | 3 | 1 | 1 | 72.1 | 8.95 | 9.3 |  |  |  |  |  | 2 |
| 1081 | 31.31 | 2 | 3700 | 50 | 3 | 3 | 1 | 1 | 93.1 | 13.93 | 13.6 | 273.57 | 5.04 | 8.36 | 56.62 | 1.03 | 5 |
| 1082 | 2.63 | 2 | 2450 | 49 | 3 | 3 | 1 | 1 | 55.4 | 4.58 | 11 |  |  |  |  |  | 0 |
| 1083 | 34.63 | 1 | 4250 | 50 | 4 | 3 | 1 | 1 | 93.1 | 14.92 | 13.8 |  |  | 24.72 | 45.60 |  | 4 |
| 1084 | 5.39 | 2 | 3500 | 51 | 2 | 2 | 1 | 1 | 65.1 | 7.28 | 11.5 |  |  |  |  |  | 0 |
| 1085 | 27.1 | 2 | 3400 | 50 | 3 | 3 | 1 | 1 | 91 | 14.64 | 12.8 |  |  | 14.06 | 72.00 | 1.03 | 5 |
| 1086 | 5.91 | 2 | 3000 | 50 | 3 | 2 | 2 | 1 | 66.4 | 7.88 | 14.1 |  |  |  |  |  | 2 |
| 1087 | 27.76 | 1 | 2350 | 50 | 3 | 2 | 1 | 1 | 84 | 11.07 | 10.1 | 205.84 | 2.97 | 17.85 | 33.00 | 1.05 | 5 |
| 1088 | 17.31 | 2 | 3800 | 50 | 4 | 2 | 1 | 1 | 80.1 | 9.2 | 11.8 | 238.41 | 6.01 | 7.62 | 66.07 | 1.09 | 5 |
| 1089 | 20.44 | 1 | 4150 | 53 | 3 | 3 | 1 | 1 | 87.1 | 12.77 | 11.6 | 280.48 | 2.53 | 8.07 | 47.92 | 1.15 | 5 |
| 1090 | 14.03 | 2 | 3300 | 50 | 3 | 3 | 1 | 1 | 75.9 | 10.19 | 11.7 |  |  |  |  | 1.18 | 4 |
| 1091 | 15.74 | 1 | 3700 | 55 | 3 | 3 | 2 | 1 | 91.1 | 12.36 | 11.4 | 124.68 | 2.63 |  |  | 1.06 | 3 |
| 1092 | 17.71 | 2 | 3300 | 50 | 5 | 3 | 1 | 1 | 83 | 9.92 | 12.8 | 630.52 | 3.37 | 8.96 | 76.73 | 1.20 | 5 |
| 1093 | 26.87 | 2 | 3400 | 50 | 3 | 3 | 1 | 1 | 97 | 12.02 | 12.5 | 454.38 | 3.58 | 29.58 | 92.08 | 1.08 | 5 |
| 1094 | 29.73 | 2 | 3050 | 50 | 3 | 3 | 1 | 1 | 87 | 12.13 | 12.2 | 791.44 | 2.46 | 4.87 | 69.10 | 1.06 | 6 |
| 1095 | 4.44 | 1 | 2200 | 48 | 3 | 3 | 1 | 1 | 63 | 8.44 | 9.8 |  |  |  |  |  | 0 |
| 1096 | 18.73 | 1 | 4400 | 50 | 3 | 3 | 1 | 1 | 82.8 | 12.27 | 12.1 | 551.23 | 4.10 |  |  | 1.04 | 6 |
| 1097 | 6.05 | 2 | 3200 | 50 | 2 | 2 | 1 | 1 | 66.7 | 7.53 | 10.8 |  |  |  |  |  | 0 |
| 1098 | 24.28 | 1 | 3800 | 55 | 3 | 3 | 2 | 1 | 93.2 | 12.87 | 11.5 |  |  | 16.56 | 84.13 | 0.94 | 5 |
| 1099 | 21.45 | 2 | 2600 | 47 | 3 | 2 | 2 | 1 | 79 | 9.8 | 12.6 | 527.17 | 4.69 | 61.82 | 56.62 | 1.15 | 4 |
| 1100 | 29.34 | 2 | 3200 |  | 3 | 3 | 1 | 1 | 89.5 | 11.52 | 13.9 | 296.99 | 15.69 | 20.34 | 87.17 | 1.06 | 2 |
| 1101 | 22.31 | 1 | 3500 | 50 | 3 | 3 | 1 | 1 | 83 | 10.64 | 12.3 |  |  |  |  |  | 4 |
| 1102 | 9.26 | 1 | 3700 |  | 2 | 2 | 1 | 1 | 73.7 | 10.06 | 12.4 |  |  |  |  |  | 2 |
| 1103 | 6.28 | 1 | 3000 | 50 | 3 | 3 | 1 | 1 | 64.3 | 5.958 | 11.7 |  |  |  |  |  | 0 |
| 1104 | 10.41 | 1 | 2250 | 45 | 2 | 2 | 1 | 1 | 67.6 | 8.11 | 11.3 |  |  |  |  |  | 4 |
| 1105 | 11.3 | 1 | 2950 | 49 | 3 | 3 | 1 | 1 | 74.4 | 10.655 | 12.3 |  |  |  |  |  | 1 |
| 1106 | 6.08 | 2 | 3350 | 50 | 2 | 2 | 1 | 1 | 68.3 | 6.55 | 12.7 |  |  |  |  |  | 0 |
| 1107 | 33.35 | 1 | 3100 | 50 | 3 | 2 | 1 | 1 | 88.8 | 14.255 | 14.2 |  |  |  |  |  | 6 |
| 1108 | 13.04 | 1 | 2800 | 40 | 3 | 2 | 2 | 1 | 69.1 | 8.33 | 11.8 |  |  |  |  |  | 2 |
| 1109 | 13.04 | 1 | 2800 | 40 | 3 | 2 | 2 | 1 | 68.1 | 7.75 | 11.1 |  |  |  |  |  | 2 |
| 1110 | 15.67 | 2 | 2600 | 48 | 2 | 2 | 1 | 1 | 77.1 | 8.45 | 13.1 | 295.08 | 3.15 | 12.41 | 47.69 | 1.00 | 2 |
| 1111 | 15.67 | 2 | 3150 | 48 | 2 | 2 | 1 | 1 | 77.2 | 8.48 | 12.4 |  |  | 8.23 | 45.73 | 1.15 | 2 |
| 1112 | 10.71 | 2 | 3300 | 48 | 3 | 3 | 1 | 1 | 70.2 | 9.08 | 11.3 |  |  |  |  |  | 2 |
| 1113 | 6.31 | 1 | 3000 | 50 | 4 | 4 | 1 | 1 | 70 | 9.02 | 10.8 |  |  |  |  |  | 0 |
| 1114 | 24.97 | 2 | 2800 | 48 | 2 | 2 | 1 | 1 | 80.3 | 9.72 | 11.3 | 284.19 | 2.80 | 1.49 | 41.22 | 1.02 | 7 |
| 1115 | 21.91 | 1 | 2950 | 50 | 2 | 2 | 1 | 1 | 85.1 | 11.66 | 12.6 | 222.44 | 3.56 | 14.89 | 44.08 | 1.11 | 6 |
| 1116 | 2 | 1 | 2900 | 50 | 3 | 4 | 1 | 2 | 59.1 | 5.16 | 10.1 |  |  |  |  |  | 1 |
| 1117 | 18.73 | 2 | 3480 | 52 | 3 | 3 | 1 | 1 | 81.1 | 10.68 | 12.8 | 282.95 | 7.12 |  |  | 1.51 | 5 |
| 1118 | 28.65 | 1 | 4500 | 48 | 3 | 2 | 2 | 1 | 86.2 | 12.1 | 12.7 | 212.59 | 6.16 | 7.59 | 43.29 | 0.99 | 5 |
| 1119 | 27.17 | 1 | 3480 | 50 | 3 | 3 | 1 | 1 | 86.1 | 11.05 | 13.7 | 317.27 | 2.71 | 9.03 | 55.21 | 1.17 | 4 |
| 1120 | 34.07 | 1 | 2400 | 49 | 2 | 2 | 1 | 1 | 84.7 | 9.55 | 12.3 | 373.88 | 7.35 | 32.27 | 43.69 | 1.07 | 6 |
| 1121 | 16.36 | 2 | 3000 | 50 | 4 | 4 | 1 | 2 | 78.8 | 9.05 | 14.7 |  |  |  |  |  | 2 |
| 1122 | 20.9 | 2 | 3450 | 50 | 3 | 4 | 1 | 1 | 82.7 | 10.645 | 12.1 | 360.40 | 8.44 | 29.17 | 78.60 | 1.10 | 5 |
| 1123 | 7.43 | 1 | 2400 | 51 | 5 | 5 | 2 | 2 | 69 | 7.825 | 11.6 |  |  |  |  |  | 1 |
| 1124 | 10.94 | 1 | 3250 | 48 | 2 | 2 | 2 | 1 | 75.5 | 10.755 | 12.5 |  |  |  |  |  | 2 |
| 1125 | 25.79 | 2 | 3000 | 48 | 1 | 1 | 1 | 1 | 82.8 | 9.21 | 13.1 | 244.00 | 10.60 | 6.17 | 51.24 | 1.17 | 3 |
| 1126 | 13.08 | 1 | 3200 | 50 | 2 | 1 | 1 | 1 | 76.5 | 11.055 | 11.6 |  |  |  |  |  | 4 |
| 1127 | 19.35 | 2 | 3250 | 50 | 3 | 3 | 1 | 1 | 78.8 | 9.355 | 11.9 | 252.34 | 5.31 | 25.87 | 63.93 | 1.14 | 3 |
| 1128 | 9.66 | 2 | 3900 | 52 | 3 | 3 | 1 | 1 | 68.2 | 8.61 | 11.6 |  |  |  |  |  | 2 |
| 1129 | 14.13 | 2 | 3100 | 50 | 3 | 3 | 1 | 1 | 73.6 | 10.69 | 12.5 |  |  |  |  |  | 4 |
| 1130 | 20.86 | 1 | 4000 | 50 | 4 | 3 | 2 | 1 | 84.2 | 10.45 | 11.8 | 381.78 | 3.11 | 5.26 | 51.30 | 1.08 | 3 |
| 1131 | 27.4 | 2 | 3000 | 52 | 4 | 2 | 1 | 2 | 78.9 | 9.355 | 11.8 |  |  |  |  |  | 5 |
| 1132 | 13.11 | 2 | 3450 | 50 | 3 | 3 | 2 | 1 | 77.3 | 10.33 | 13 |  |  |  |  |  | 3 |
| 1133 | 35.42 | 2 | 3750 | 58 | 3 | 2 | 2 | 1 | 92.2 | 13.07 | 11.9 |  |  |  |  |  | 5 |
| 1134 | 15.54 | 2 | 3680 | 50 | 4 | 3 | 1 | 1 | 75.1 | 9.15 | 11.9 | 137.24 | 4.15 | 0.00 | 62.47 | 0.95 | 2 |
| 1135 | 4.63 | 2 | 3100 | 50 | 3 | 3 | 1 | 2 | 66.1 | 7.155 | 12.6 |  |  |  |  |  | 0 |
| 1136 | 8.94 | 1 | 2900 | 50 | 4 | 2 | 2 | 1 | 70.2 | 8.035 | 10.7 |  |  |  |  |  | 3 |
| 1137 | 26.35 | 2 | 3450 | 60 | 5 | 3 | 1 | 1 | 80.7 | 9.52 | 12.9 |  |  |  |  |  | 2 |
| 1138 | 31.7 | 1 | 3900 | 60 | 3 | 3 | 1 | 2 | 91.6 | 13.1 | 12 |  |  | 32.93 | 81.88 | 0.83 | 1 |
| 1139 | 18.33 | 2 | 2900 | 48 | 3 | 3 | 1 | 2 | 77.3 | 9.93 | 10.8 | 380.74 | 3.63 | 17.40 | 78.19 | 0.87 | 3 |
| 1140 | 23.98 | 1 | 3450 | 50 | 3 | 3 | 1 | 1 | 91 | 13.655 | 13.6 | 153.63 | 5.27 | 7.44 | 54.24 | 0.97 | 4 |
| 1141 | 33.18 | 1 | 3000 | 55 | 3 | 3 | 2 | 2 | 94.5 | 13.965 | 13.1 |  | 4.21 | 28.80 | 58.00 | 0.95 | 5 |
| 1142 | 17.68 | 1 | 3000 | 50 | 4 | 3 | 1 | 1 | 82 | 10.064 | 10.1 |  |  |  |  |  | 2 |
| 1143 | 13.67 | 1 | 3650 | 50 | 3 | 3 | 1 | 1 | 77.5 | 9.105 | 11.4 |  |  |  |  |  | 1 |
| 1144 | 33.31 | 1 | 3550 | 56 | 3 | 3 | 2 | 1 | 91.8 | 13.215 | 12.7 | 286.75 | 3.45 | 8.11 | 65.75 | 1.00 | 2 |
| 1145 | 28.78 | 2 | 2800 |  | 3 | 3 | 1 | 1 | 88.8 | 12.13 | 13.7 | 101.15 | 1.33 | 25.79 | 60.63 | 1.20 | 3 |
| 1146 | 18.46 | 2 | 3100 | 50 | 3 | 3 | 1 | 1 | 81.6 | 11.755 | 12.7 |  | 5.47 | 12.15 | 79.09 | 0.91 | 3 |
| 1147 | 8.25 | 2 | 3000 | 50 | 4 | 3 | 2 | 1 | 67.3 | 7.485 | 10.1 |  |  |  |  |  | 3 |
| 1148 | 31.31 | 2 | 2750 | 53 | 3 | 3 | 2 | 1 | 83.1 | 10.99 | 12.7 |  |  | 20.59 | 56.92 | 0.98 | 4 |
| 1149 | 5.36 | 1 | 3500 | 50 | 3 | 3 | 1 | 1 | 66.5 | 7.815 | 10.9 |  |  |  |  |  | 1 |
| 1150 | 7.2 | 2 | 3550 | 50 | 2 | 2 | 1 | 1 | 66.5 | 9.2 | 12.2 |  |  |  |  |  | 4 |
| 1151 | 9.63 | 1 | 3200 | 50 | 4 | 3 | 2 | 2 | 74.3 | 8.795 | 10.5 |  |  |  |  |  | 2 |
| 1152 | 27.7 | 2 | 3150 | 52 | 2 | 2 | 1 | 1 | 87.6 | 11.805 | 13.1 |  |  |  |  |  | 3 |
| 1153 | 6.18 | 2 | 3500 | 50 | 4 | 4 | 1 | 2 | 66.1 | 6.645 | 12 |  |  |  |  |  | 0 |
| 1154 | 19.55 | 1 | 3000 | 52 | 3 | 3 | 2 | 1 | 86.5 | 11 | 12 |  |  | 20.97 | 74.49 | 0.97 | 4 |
| 1155 | 9.49 | 2 | 3200 | 51 | 3 | 2 | 1 | 2 | 67.6 | 7.23 | 11.7 |  |  |  |  |  | 2 |
| 1156 | 30.26 | 2 | 3600 | 56 | 2 | 1 | 1 | 1 | 83.2 | 12.025 | 12.4 | 409.04 | 3.67 | 34.00 | 47.92 | 0.74 | 6 |
| 1157 | 12.42 | 2 | 3800 | 50 | 3 | 3 | 1 | 1 | 77.3 | 10.95 | 11.4 |  |  |  |  |  | 2 |
| 1158 | 17.18 | 2 | 2100 | 48 | 3 | 2 | 2 | 1 | 75 | 9.005 | 11.1 |  |  |  |  |  | 3 |
| 1159 | 17.18 | 2 | 2350 | 48 | 3 | 2 | 2 | 1 | 75.5 | 9.005 | 11.2 |  |  |  |  |  | 3 |
| 1160 | 27.43 | 1 | 3150 | 56 | 4 | 4 | 2 | 1 | 90.1 | 13.345 | 13.6 | 690.81 | 5.19 | 32.81 | 79.56 | 0.87 | 5 |
| 1161 | 8.64 | 2 | 3200 | 50 | 4 | 4 | 1 | 1 | 69.9 | 7.695 | 10 |  |  |  |  |  | 0 |
| 1162 | 19.94 | 2 | 3100 | 52 | 2 | 2 | 2 | 1 | 82.3 | 10.725 | 11.6 | 615.82 | 7.99 | 13.59 | 46.80 | 0.92 | 4 |
| 1163 | 20.76 | 2 | 3650 | 52 | 4 | 3 | 1 | 1 | 82 | 12.1 | 12.3 |  |  |  |  |  | 4 |
| 1164 | 6.93 | 1 | 3200 | 50 | 5 | 5 | 1 | 1 | 67.1 | 7.4 | 11 |  |  |  |  |  | 2 |
| 1165 | 34.3 | 2 | 2800 | 52 | 3 | 2 | 1 | 1 | 89.8 | 12.795 | 12 | 309.45 | 5.80 | 28.55 | 71.92 | 1.04 | 1 |
| 1166 | 1.12 | 2 | 3350 | 50 | 5 | 4 | 1 | 1 | 56.1 | 4.335 | 15 | 146.91 | 2.74 |  |  | 0.91 | 0 |
| 1167 | 23.46 | 2 | 3550 | 51 | 5 | 3 | 1 | 1 | 84.9 | 13.585 | 12.6 | 612.44 | 4.96 | 50.83 | 66.89 | 1.29 | 3 |
| 1168 | 17.02 | 1 |  |  | 2 | 2 | 1 | 1 | 82.3 | 11.705 | 13.2 |  |  | 20.39 | 64.88 |  | 4 |
| 1169 | 35.94 | 1 | 4000 |  | 3 | 3 | 1 | 1 | 98.1 | 14.275 | 13.3 |  |  | 17.78 | 56.62 |  | 3 |
| 1170 | 24.84 | 1 | 3350 | 50 | 3 | 3 | 1 | 1 | 87.6 | 11.16 | 14.3 |  |  |  |  |  | 6 |
| 1171 | 3.71 | 2 | 3100 | 51 | 3 | 3 | 1 | 2 | 63.9 | 7.105 | 11.8 |  |  |  |  |  | 0 |
| 1172 | 24.11 | 2 | 4300 | 50 | 4 | 4 | 1 | 1 | 88.6 | 10.855 | 12.9 | 574.15 | 7.97 |  |  | 1.36 | 6 |
| 1173 | 31.93 | 2 | 3500 | 50 | 3 | 3 | 1 | 1 | 95.7 | 13.37 | 12.2 | 491.73 | 4.28 |  |  | 1.00 | 7 |
| 1174 | 30.06 | 2 | 2950 | 50 | 3 | 2 | 1 | 1 | 85.1 | 11.65 | 13.1 | 260.47 | 3.07 |  |  | 1.14 | 3 |
| 1175 | 13.04 | 2 | 3600 | 55 | 3 | 2 | 1 | 1 | 76.2 | 9.805 | 11.4 |  |  |  |  |  | 4 |
| 1176 | 9.3 | 2 | 3500 | 55 | 4 | 3 | 2 | 1 | 68.6 | 8.425 | 12.9 |  |  |  |  |  | 0 |
| 1177 | 11.99 | 1 | 3200 | 48 | 3 | 3 | 2 | 1 | 73.1 | 8.895 | 12.5 |  |  |  |  |  | 2 |
| 1178 | 32.85 | 1 | 3000 | 50 | 2 | 3 | 1 | 1 | 89.5 | 12.655 | 12 | 277.21 | 4.87 | 13.17 | 40.19 | 1.30 | 2 |
| 1179 | 9.63 | 2 | 3450 | 50 | 3 | 2 | 1 | 1 | 76.1 | 7.665 | 10.4 | 631.35 | 5.23 |  |  | 1.12 | 2 |
| 1180 | 6.6 | 2 | 2750 | 50 | 4 | 5 | 2 | 2 | 63.8 | 6.785 | 12.4 |  |  |  |  |  | 1 |
| 1181 | 17.22 | 2 | 3900 | 50 | 3 | 3 | 2 | 1 | 77.8 | 9.62 | 12.8 | 675.64 | 7.60 | 46.30 | 51.15 | 1.12 | 6 |
| 1182 | 21.68 | 2 | 2900 | 50 | 3 | 2 | 2 | 1 | 79.6 | 9.55 | 13 |  |  |  |  |  | 3 |
| 1183 | 34.5 | 1 | 3400 | 55 | 2 | 1 | 1 | 1 | 80.1 | 9.2 | 12.2 | 367.34 | 4.96 | 20.31 | 55.06 | 1.36 | 6 |
| 1184 | 15.84 | 1 | 4050 | 50 | 3 | 2 | 2 | 1 | 78.4 | 11.7 | 14.3 |  |  |  |  |  | 4 |
| 1185 | 24.74 | 2 | 3400 | 50 | 2 | 1 | 2 | 1 | 83.9 | 12.545 | 13.2 | 271.56 | 5.49 | 15.64 | 35.19 | 0.98 | 3 |
| 1186 | 1.94 | 2 | 3300 | 50 | 2 | 2 | 1 | 1 | 56.1 | 4.65 | 10.6 |  |  |  |  |  | 0 |
| 1187 | 1.61 | 1 | 3500 | 50 | 3 | 3 | 2 | 2 | 56.4 | 5.675 | 11.7 |  |  |  |  |  | 0 |
| 1188 | 16.23 | 2 | 3600 | 50 | 3 | 3 | 1 | 1 | 89.8 | 9.545 | 11.6 | 143.70 | 9.23 | 13.83 | 75.21 | 1.13 | 2 |
| 1189 | 22.8 | 2 | 3850 | 50 | 4 | 4 | 2 | 1 | 84.3 | 11.095 | 12.2 |  |  |  |  |  | 4 |
| 1190 | 21.68 | 2 | 3500 | 50 | 3 | 3 | 1 | 1 | 83.7 | 10.285 | 13 | 131.40 | 6.44 | 12.09 | 54.80 | 0.97 | 3 |
| 1191 | 3.78 | 2 | 3500 | 50 | 3 | 3 | 1 | 1 | 61 | 7.885 | 10.5 |  |  |  |  |  | 0 |
| 1192 | 5.09 | 1 | 3250 | 51 | 3 | 3 | 1 | 1 | 67.6 | 6.815 | 11.7 |  |  |  |  |  | 0 |
| 1193 | 32.95 | 2 | 3050 | 50 | 3 | 3 | 1 | 1 | 91.8 | 12.785 | 12.1 | 292.96 | 7.35 | 35.29 | 53.32 | 1.23 | 5 |
| 1194 | 3.52 | 1 | 4000 | 50 | 3 | 3 | 1 | 2 | 66.8 | 8.975 | 11.6 | 197.94 | 8.94 | 11.34 | 52.37 | 0.80 | 0 |
| 1195 | 18.33 | 2 | 3250 | 50 | 3 | 3 | 1 | 1 | 81.5 | 12.865 | 11 | 506.15 | 7.41 | 10.21 | 52.61 | 0.96 | 7 |
| 1196 | 23.23 | 1 | 3450 | 50 | 3 | 3 | 1 | 1 | 82.7 | 9.14 | 10.8 |  |  | 25.40 | 55.31 |  | 2 |
| 1197 | 9.17 | 1 | 3450 | 50 | 3 | 4 | 1 | 1 | 81.2 | 11.875 | 12.5 | 412.43 | 2.09 | 12.85 | 38.97 | 1.18 | 6 |
| 1198 | 32.43 | 2 | 2500 | 46 | 3 | 4 | 1 | 1 | 88.5 | 11.5 | 12.4 | 352.11 | 3.09 | 189.19 | 46.76 | 1.03 | 2 |
| 1199 | 33.38 | 1 | 3750 | 50 | 4 | 4 | 1 | 1 | 78 | 12.435 | 13.3 | 384.65 | 11.69 | 19.18 | 58.24 | 1.30 | 4 |
| 1200 | 18 | 2 | 3200 |  | 4 | 3 | 1 | 1 | 81.1 | 9.94 | 13.1 |  |  |  |  | 1.32 | 3 |
| 1201 | 6.87 | 1 | 3850 |  | 3 | 3 | 1 | 2 | 71 | 11.235 | 13.9 |  |  |  |  |  | 0 |
| 1202 | 17.08 | 1 | 3200 |  | 3 | 2 | 1 | 1 | 81.1 | 12.03 | 11.5 |  |  | 18.18 | 54.55 |  | 4 |
| 1203 | 5.52 | 2 | 2750 | 50 | 4 | 5 | 1 | 2 | 65 | 7.545 | 12.1 |  |  |  |  |  | 0 |
| 1204 | 6.6 | 2 | 2550 |  | 2 | 2 | 2 | 2 | 64.4 | 7.045 | 12.1 |  |  |  |  |  | 2 |
| 1205 | 13.73 | 2 | 3350 |  | 1 | 1 | 1 | 1 | 78.6 | 9.335 | 12.2 |  |  |  |  |  | 2 |
| 1206 | 33.48 | 1 | 3700 |  | 3 | 3 | 1 | 1 | 95.1 | 15.709 | 12.6 |  |  | 26.17 | 58.00 | 1.16 | 7 |
| 1207 | 21.13 | 2 | 3250 |  | 4 | 3 | 1 | 1 | 80.1 | 9.705 | 12.3 |  |  |  |  |  | 3 |
| 1208 | 18 | 1 | 3500 |  | 1 | 2 | 1 | 1 | 79.1 | 10.22 | 11.2 | 456.78 | 5.17 | 3.47 | 62.16 | 0.81 | 4 |
| 1209 | 9.63 | 1 | 3400 |  | 2 | 2 | 1 | 2 | 73.4 | 10.585 | 11.5 |  |  |  |  |  | 1 |
| 1210 | 31.64 | 1 | 3600 |  | 2 | 2 | 1 | 1 | 86.2 | 10.825 | 12.2 | 377.13 | 3.54 | 26.35 | 39.42 | 0.91 | 2 |
| 1211 | 16.99 | 1 | 4050 |  | 3 | 2 | 1 | 1 | 76.6 | 10.215 | 11.7 |  |  |  |  |  | 2 |
| 1212 | 14.95 | 1 | 3400 | 61 | 3 | 3 | 1 | 1 | 76.4 | 10.41 | 12.1 |  |  | 0.00 | 62.79 | 0.96 | 5 |
| 1213 | 19.02 | 1 | 2600 | 50 | 5 | 5 | 1 | 1 | 81.5 | 9.7 | 13.3 | 449.60 | 2.41 | 27.73 | 74.77 | 0.75 | 4 |
| 1214 | 23.72 | 2 | 3750 |  | 3 | 3 | 1 | 1 | 80.2 | 11.065 | 11.6 | 433.22 | 4.58 | 6.50 | 59.73 | 0.79 | 4 |
| 1215 | 5.62 | 2 | 3120 |  | 3 | 2 | 1 | 1 | 63 | 6.855 | 9.6 |  |  |  |  |  | 0 |
| 1216 | 17.74 | 1 | 2600 | 47 | 4 | 3 | 1 | 1 | 76.3 | 9.24 | 12.1 | 447.23 | 3.97 | 20.62 | 98.40 | 1.07 | 4 |
| 1217 | 26.78 | 1 | 3450 | 52 | 3 | 3 | 1 | 1 | 85.9 | 10.465 | 13 |  |  |  |  |  | 3 |
| 1218 | 5.36 | 2 | 3700 | 53 | 6 | 4 | 1 | 1 | 62 | 6.61 | 10.3 |  |  |  |  |  | 0 |
| 1219 | 20.93 | 2 | 2400 | 50 | 3 | 3 | 2 | 2 | 82.1 | 9.715 | 12.6 | 237.15 | 3.80 | 23.37 | 73.14 | 0.81 | 5 |
| 1220 | 25.13 | 1 | 3700 | 50 | 4 | 6 | 2 | 2 | 89 | 12.96 | 12.6 | 301.02 | 5.59 | 12.13 | 101.67 | 1.04 | 5 |
| 1221 | 13.17 | 1 | 3250 | 48 | 3 | 3 | 1 | 1 | 70.8 | 7.915 | 12.9 | 526.86 | 7.14 | 38.02 | 104.41 | 1.17 | 6 |
| 1222 | 2.3 | 1 | 2600 |  | 2 | 2 | 1 | 2 | 55.5 | 5.875 | 10.7 |  |  |  |  |  | 1 |
| 1223 | 16.53 | 2 | 3350 |  | 4 | 3 | 1 | 1 | 78.3 | 9.425 | 12.4 | 509.30 | 1.84 | 9.09 | 62.16 | 1.11 | 5 |
| 1224 | 9.53 | 2 | 2800 | 48 | 3 | 3 | 1 | 1 | 67.1 | 7.62 | 11.3 |  |  |  |  |  | 3 |
| 1225 | 25.69 | 1 | 3200 |  | 3 | 3 | 1 | 1 | 84.3 | 11.195 | 11.5 |  |  | 4.77 | 62.47 | 0.89 | 3 |
| 1226 | 7.03 | 1 | 3000 |  | 3 | 3 | 1 | 1 | 66.1 | 8.215 | 11.7 |  |  |  |  |  | 1 |
| 1227 | 2.86 | 2 | 3100 |  | 1 | 1 | 1 | 1 | 54.5 | 5.215 | 12.5 |  |  |  |  |  | 0 |
| 1228 | 17.41 | 1 | 3700 | 50 | 6 | 4 | 1 | 1 | 78 | 10.19 | 11.5 |  |  | 12.66 | 65.54 | 1.25 | 4 |
| 1229 | 13.7 | 2 | 3700 | 53 | 6 | 5 | 1 | 1 | 77.1 | 9.46 | 8.9 | 385.17 | 14.56 | 28.74 | 114.58 | 1.28 | 5 |
| 1230 | 4.99 | 1 | 3850 | 55 | 3 | 3 | 1 | 1 | 66.7 | 8.1 | 11.3 |  |  |  |  |  | 1 |
| 1231 | 2 | 2 | 3300 | 51 | 5 | 6 | 2 | 1 | 57 | 5.601 | 8.3 |  |  |  |  |  | 0 |
| 1232 | 33.41 | 1 | 3700 | 50 | 6 | 3 | 1 | 1 | 91.9 | 13.8 | 12.6 | 372.87 | 6.27 | 29.56 | 65.54 | 1.39 | 4 |
| 1233 | 20.17 | 2 | 2950 | 50 | 5 | 4 | 1 | 1 | 90 | 13 | 12.8 |  | 4.39 | 23.89 | 45.98 | 0.98 | 7 |
| 1234 | 33.38 | 1 | 3400 | 51 | 4 | 3 | 1 | 1 | 93 | 13.12 | 13 | 295.35 | 1.25 | 20.51 | 47.94 |  | 4 |
| 1235 | 16.79 | 2 | 3050 | 50 | 2 | 3 | 1 | 1 | 81.5 | 11.205 | 12.6 | 760.66 | 5.71 | 4.64 | 104.41 |  | 6 |
| 1236 | 23.85 | 1 | 3150 | 50 | 3 | 3 | 1 | 1 | 85.6 | 11.905 | 12.9 |  |  | 16.56 | 67.48 |  | 4 |
| 1237 | 23.43 | 1 | 3850 | 51 | 3 | 3 | 1 | 1 | 90.5 | 12.625 | 12.2 | 278.30 | 4.07 | 18.03 | 58.28 |  | 5 |
| 1238 | 22.05 | 1 | 3200 | 49 | 4 | 3 | 1 | 1 | 84 | 11.035 | 11.6 | 505.33 | 5.66 | 24.28 | 50.14 |  | 4 |
| 1239 | 20.86 | 1 | 3150 | 50 | 6 | 5 | 1 | 1 | 86.5 | 11.835 | 13.5 | 624.70 | 6.02 | 40.85 | 79.87 |  | 6 |
| 1240 | 30.82 | 1 | 3200 | 50 | 5 | 5 | 2 | 1 | 88.6 | 11.73 | 12.2 | 342.33 | 4.15 | 14.96 | 72.34 |  | 4 |
| 1241 | 9 | 1 | 3280 | 51 | 2 | 3 | 2 | 2 | 69.6 | 7.9 | 10.4 |  |  |  |  |  | 3 |
| 1242 | 11.86 | 1 | 3200 | 45 | 3 | 3 | 1 | 1 | 71.1 | 9.65 | 13.1 |  |  |  |  |  | 3 |
| 1243 | 3.12 | 1 | 4050 | 55 | 3 | 3 | 1 | 1 | 60.6 | 7.76 | 11.3 |  |  |  |  |  | 0 |
| 1244 | 3.91 | 1 | 2905 | 50 | 3 | 4 | 1 | 1 | 62 | 6.62 | 9.8 |  |  |  |  |  | 0 |
| 1245 | 11.2 | 2 | 3550 | 55 | 3 | 2 | 1 | 1 | 74 | 10.01 | 11 |  |  |  |  |  | 3 |
| 1246 | 31.9 | 2 | 2900 | 50 | 4 | 3 | 2 | 1 | 93 | 11.675 | 12.2 |  |  | 33.67 | 73.94 |  | 4 |
| 1247 | 28.39 | 1 | 1360 | 50 | 2 | 2 | 1 | 1 | 87 | 11.765 | 12.7 |  |  | 12.43 | 36.96 |  | 4 |
| 1248 | 35.19 | 1 | 3200 | 50 | 2 | 2 | 1 | 1 | 91.5 | 12.585 | 12.7 | 150.49 | 4.31 | 20.63 | 47.94 |  | 5 |
| 1249 | 28.71 | 2 | 3500 | 50 | 3 | 4 | 1 | 1 | 86 | 10.405 | 11.9 | 530.30 | 10.32 | 35.99 | 62.50 |  | 3 |
| 1250 | 34.99 | 2 | 3400 | 50 | 3 | 3 | 1 | 1 | 87.5 | 11.315 | 12.3 | 634.54 | 5.41 | 25.52 | 58.57 |  | 4 |
| 1251 | 12.32 | 2 | 2535 | 50 | 3 | 3 | 2 | 1 | 71.9 | 8.815 | 13.1 |  |  |  |  |  | 4 |
| 1252 | 6.74 | 2 | 3100 | 50 | 3 | 3 | 1 | 1 | 68 | 9.64 | 12.7 |  |  |  |  |  | 0 |
| 1253 | 10.09 | 2 | 3000 | 49 | 3 | 3 | 1 | 1 | 73.7 | 8.65 | 12.1 |  |  |  |  |  | 2 |
| 1254 | 11.99 | 2 | 2900 | 50 | 3 | 3 | 2 | 1 | 84.5 | 11.265 | 12.1 | 176.62 | 3.48 | 6.55 | 43.10 |  | 6 |
| 1255 | 34.4 | 2 | 3000 | 49 | 2 | 2 | 2 | 1 | 86 | 11.01 | 11.1 | 341.38 | 2.92 | 7.07 | 77.19 |  | 4 |
| 1256 | 17.05 | 1 | 3550 | 50 | 3 | 2 | 1 | 1 | 80.3 | 10.455 | 13.2 | 632.07 | 5.36 | 25.05 | 42.71 |  | 3 |
| 1257 | 22.51 | 2 | 3500 | 52 | 3 | 2 | 1 | 1 | 81.2 | 11.425 | 13.5 | 337.09 | 22.18 | 30.29 | 80.05 |  | 6 |
| 1258 | 36.01 | 2 | 3000 | 50 | 2 | 1 | 1 | 1 | 86.2 | 11.39 | 12.2 | 261.07 | 6.15 | 23.48 | 57.50 |  | 5 |
| 1259 | 3.15 | 1 | 2900 | 50 | 3 | 2 | 1 | 1 | 70 | 7.725 | 13.6 |  |  |  |  |  | 3 |
| 1260 | 9.17 | 1 | 3700 | 55 | 3 | 3 | 1 | 1 | 69.1 | 8.54 | 13.1 |  |  |  |  |  | 1 |
| 1261 | 10.58 | 1 | 3800 | 50 | 2 | 2 | 1 | 1 | 74.1 | 10.685 | 12 |  |  |  |  |  | 2 |
| 1262 | 32.43 | 1 | 3000 | 50 | 2 | 2 | 1 | 1 | 91.5 | 12.65 | 12.5 |  |  |  |  |  | 3 |
| 1263 | 35.38 | 1 | 2500 | 50 | 3 | 2 | 1 | 1 | 92 | 12.605 | 11.9 | 264.30 | 4.95 | 16.70 | 60.91 |  | 5 |
| 1264 | 33.61 | 1 | 3800 | 50 | 2 | 2 | 1 | 1 | 94.5 | 13.93 | 11.5 | 327.69 | 9.15 | 13.06 | 43.29 |  | 4 |
| 1265 | 30.85 | 2 | 3000 | 49 | 3 | 3 | 1 | 1 | 86.7 | 12.155 | 11.7 | 201.59 | 1.56 | 19.83 | 100.57 |  | 6 |
| 1266 | 21.39 | 1 | 3650 | 51 | 2 | 3 | 1 | 1 | 85.1 | 11.235 | 13.7 |  | 6.78 | 19.85 | 59.96 |  | 6 |
| 1267 | 30.16 | 1 | 4000 | 55 | 3 | 2 | 1 | 1 | 88.9 | 11.845 | 13.4 |  |  | 11.91 | 40.86 |  | 4 |
| 1268 | 29.34 | 1 | 3000 | 57 | 3 | 2 | 1 | 1 | 84.1 | 11.405 | 10 |  |  |  |  |  | 4 |
| 1269 | 3.32 | 2 | 3500 | 50 | 4 | 3 | 2 | 1 | 61 | 6.21 | 11.5 |  |  |  |  |  | 0 |
| 1270 | 21.09 | 2 | 3350 | 50 | 3 | 3 | 1 | 1 | 82.1 | 9.985 | 12.1 |  |  |  |  |  | 5 |
| 1271 | 27.27 | 2 | 3100 | 45 | 4 | 3 | 1 | 1 | 80.1 | 10.24 | 13.2 | 147.69 | 2.13 | 10.61 | 42.64 |  | 6 |
| 1272 | 25.56 | 2 | 3600 | 49 | 2 | 1 | 1 | 1 | 83.6 | 11.25 | 11.8 |  |  | 9.21 | 42.13 |  | 3 |
| 1273 | 29.6 | 1 | 3400 | 49 | 3 | 3 | 1 | 1 | 89.5 | 11.985 | 10.9 | 152.06 | 3.09 | 22.18 | 45.88 |  | 5 |
| 1274 | 11.99 | 2 | 2900 | 50 | 3 | 3 | 1 | 1 | 74.5 | 9.45 | 12.9 |  |  |  |  |  | 6 |
| 1275 | 2.46 | 2 | 3100 | 50 | 2 | 2 | 1 | 1 | 59.2 | 5.19 | 8.6 |  |  |  |  |  | 0 |
| 1276 | 24.64 | 2 | 3500 | 53 | 3 | 3 | 1 | 1 | 83.1 | 10.83 | 13.5 |  |  | 32.31 | 50.59 |  | 6 |
| 1277 | 29.08 | 1 | 3100 | 49 | 3 | 2 | 1 | 1 | 84.5 | 9.8 | 12 |  |  | 28.35 | 73.18 |  | 6 |
| 1278 | 12.02 | 1 | 3150 | 50 | 3 | 1 | 1 | 2 | 72.9 | 9.55 | 10.9 |  |  |  |  |  | 6 |
| 1279 | 4.17 | 2 | 3050 | 50 | 3 | 4 | 1 | 2 | 63.2 | 6.275 | 13 |  |  |  |  |  | 0 |
| 1280 | 7.75 | 1 | 3400 | 50 | 4 | 4 | 2 | 1 | 70.6 | 7.47 | 11.2 |  |  |  |  |  | 3 |
| 1281 | 11.66 | 2 | 3200 | 44 | 3 | 3 | 1 | 2 | 72.1 | 9.1 | 11 |  |  |  |  |  | 3 |
| 1282 | 20.4 | 2 | 3000 | 48 | 3 | 3 | 1 | 1 | 77.3 | 10.155 | 10.9 | 363.41 | 1.82 | 7.01 | 69.80 |  | 5 |
| 1283 | 20.83 | 1 | 3400 | 51 | 4 | 3 | 1 | 1 | 81 | 10.785 | 11.7 |  |  | 60.62 | 67.68 |  | 5 |
| 1284 | 6.31 | 1 | 3300 | 53 | 3 | 3 | 1 | 2 | 73 | 7.97 | 12.1 |  |  |  |  |  | 0 |
| 1285 | 4.5 | 2 | 3900 | 50 | 2 | 2 | 2 | 2 | 66.2 | 6.325 | 13 |  |  |  |  |  | 0 |
| 1286 | 10.94 | 2 | 3900 | 55 | 3 | 3 | 1 | 2 | 77 | 10.265 | 12.7 |  |  |  |  |  | 6 |
| 1287 | 20.63 | 2 | 3000 | 49 | 3 | 2 | 1 | 2 | 86.2 | 12.095 | 11.4 |  |  |  |  |  | 3 |
| 1288 | 10.28 | 2 | 2950 | 50 | 3 | 2 | 1 | 1 | 69 | 6.985 | 10.7 |  |  |  |  |  | 3 |
| 1289 | 10.64 | 2 | 3700 | 55 | 3 | 3 | 1 | 1 | 79.4 | 9.55 | 11.1 |  |  |  |  |  | 5 |
| 1290 | 11.6 | 1 | 2800 | 48 | 2 | 2 | 1 | 1 | 71.9 | 7.96 | 10.1 |  |  |  |  |  | 3 |
| 1291 | 2.53 | 2 | 3600 | 55 | 3 | 3 | 1 | 1 | 61.8 | 5.95 | 9.3 |  |  |  |  |  | 1 |
| 1292 | 4.73 | 2 | 3600 | 51 | 2 | 1 | 2 | 1 | 65 | 7.84 | 12.6 |  |  |  |  |  | 0 |
| 1293 | 17.94 | 1 | 3200 | 50 | 1 | 1 | 1 | 1 | 82.1 | 13.5 | 13.1 | 557.06 | 6.57 | 22.37 | 60.52 |  | 5 |
| 1294 | 26.97 | 2 | 3200 | 50 | 4 | 3 | 1 | 1 | 88 | 12.885 | 13 |  |  | 23.33 | 57.69 |  | 5 |
| 1295 | 13.4 | 1 | 3000 | 50 | 2 | 2 | 1 | 1 | 75.9 | 10.315 | 12.4 |  |  |  |  |  | 3 |
| 1296 | 20.93 | 1 | 3750 | 52 | 3 | 2 | 1 | 1 | 85 | 13.405 | 12.8 |  |  | 6.99 | 41.18 |  | 6 |
| 1297 | 34.46 | 2 | 3000 | 50 | 2 | 2 | 1 | 1 | 89 | 14.045 | 13.2 | 312.39 | 8.23 | 7.24 | 45.41 |  | 3 |
| 1298 | 25.95 | 1 | 3500 | 50 | 2 | 2 | 1 | 1 | 80 | 9.84 | 12.2 |  |  |  |  |  | 6 |
| 1299 | 18.6 | 1 | 3000 | 50 | 3 | 2 | 1 | 2 | 79.5 | 8.895 | 14.8 |  |  | 23.94 | 81.40 |  | 2 |
| 1300 | 35.75 | 1 | 3000 | 50 | 3 | 3 | 1 | 1 | 92.6 | 12.565 | 12.4 |  |  | 20.28 | 72.00 |  | 4 |
| 1301 | 29.01 | 1 | 3225 | 50 | 3 | 3 | 2 | 1 | 90.7 | 13.26 | 13 |  |  | 12.51 | 61.72 |  | 4 |
| 1302 | 17.12 | 2 | 3950 | 54 | 2 | 2 | 2 | 2 | 76.3 | 11.125 | 11.9 |  |  | 13.08 | 54.80 |  | 5 |
| 1303 | 10.74 | 2 | 3250 | 48 | 3 | 3 | 2 | 1 | 68 | 7.64 | 11.7 |  |  |  |  |  | 5 |
| 1304 | 9.59 | 2 | 2950 | 50 | 4 | 3 | 1 | 2 | 70.1 | 9.405 | 8.6 |  |  |  |  |  | 1 |
| 1305 | 12.98 | 2 | 3500 | 50 | 3 | 2 | 1 | 1 | 75 | 10.035 | 9.8 |  |  |  |  |  | 2 |
| 1306 | 8.51 | 1 | 3600 | 55 | 4 | 4 | 2 | 2 | 71.2 | 9.185 | 12.5 |  |  | 37.30 | 54.24 |  | 3 |
| 1307 | 34.46 | 1 | 3100 | 50 | 3 | 3 | 1 | 1 | 91.1 | 11.35 | 12 |  |  | 8.18 | 53.81 |  | 4 |
| 1308 | 19.38 | 2 | 3350 | 50 | 3 | 3 | 2 | 1 | 80.3 | 10.25 | 13.3 |  |  |  |  |  | 4 |
| 1309 | 16.89 | 1 | 1806 | 50 | 2 | 2 | 1 | 1 | 68.2 | 6.565 | 12.7 |  |  |  |  |  | 1 |
| 1310 | 28.94 | 2 | 3000 | 50 | 3 | 3 | 2 | 1 | 87.1 | 13.09 | 11.2 | 271.25 | 5.66 | 24.07 | 47.52 |  | 2 |
| 1311 | 17.38 | 1 | 3000 | 50 | 3 | 3 | 1 | 2 | 80 | 8.37 | 11.7 |  |  | 20.01 | 56.35 |  | 3 |
| 1312 | 35.94 | 1 | 3500 | 50 | 4 | 3 | 1 | 1 | 91.2 | 12.38 | 11.3 |  |  |  |  |  | 6 |
| 1313 | 9.43 | 2 | 3550 | 55 | 4 | 3 | 1 | 2 | 70 | 7.6 | 12.9 |  |  |  |  |  | 3 |
| 1314 | 10.78 | 1 | 3150 | 50 | 3 | 3 | 1 | 2 | 73 | 10.11 | 11.8 |  |  |  |  |  | 3 |
| 1315 | 3.02 | 2 | 2900 | 48 | 3 | 3 | 1 | 1 | 60 | 6.665 | 10.3 |  |  |  |  |  | 0 |
| 1316 | 9.79 | 2 | 3000 | 50 | 3 | 3 | 1 | 1 | 70 | 7.38 | 12.1 |  |  |  |  |  | 4 |
| 1317 | 15.74 | 2 | 3500 | 50 | 3 | 3 | 1 | 1 | 79 | 9.355 | 10.6 | 200.89 | 6.69 | 22.07 | 45.09 |  | 4 |
| 1318 | 10.12 | 1 | 2950 | 50 | 3 | 3 | 1 | 1 | 73 | 9.425 | 12.2 |  |  |  |  |  | 3 |
| 1319 | 2.07 | 1 | 3200 | 50 | 3 | 3 | 1 | 1 | 60 | 5.59 | 9.2 |  |  |  |  |  | 1 |
| 1320 | 25.53 | 1 | 3000 | 50 | 3 | 3 | 1 | 1 | 81.5 | 11.15 | 12.1 | 209.41 | 4.16 | 12.37 | 43.38 |  | 4 |
| 1321 | 27.96 | 2 | 4400 | 68 | 3 | 3 | 1 | 1 | 91.5 | 13.085 | 13 |  |  |  |  |  | 3 |
| 1322 | 35.06 | 2 | 3000 | 50 | 3 | 3 | 1 | 1 | 92.6 | 13.44 | 13.1 | 518.01 | 3.44 | 20.87 | 59.44 |  | 3 |
| 1323 | 19.88 | 2 | 3050 | 50 | 3 | 3 | 2 | 1 | 81.1 | 10.69 | 13.8 | 326.30 | 8.11 | 26.97 | 56.07 |  | 0 |
| 1324 | 31.97 | 1 | 3250 | 50 | 3 | 4 | 1 | 1 | 90.1 | 11.65 | 12.3 | 568.14 | 4.75 | 15.59 | 67.84 |  | 6 |
| 1325 | 29.34 | 1 | 3000 | 50 | 3 | 3 | 1 | 1 | 84.6 | 11.3 | 13.6 |  |  | 0.00 | 36.70 |  | 5 |
| 1326 | 17.84 | 1 | 3400 | 54 | 3 | 3 | 2 | 1 | 76.3 | 10.06 | 11.9 | 608.65 | 3.92 | 4.90 | 62.81 |  | 5 |
| 1327 | 19.94 | 2 | 2950 | 52 | 3 | 3 | 1 | 1 | 82.6 | 11.28 | 11.9 | 673.82 | 5.06 | 20.27 | 113.34 |  | 5 |
| 1328 | 21.09 | 1 | 2250 | 50 | 2 | 2 | 1 | 1 | 79 | 9.71 | 13 | 337.56 | 5.97 | 8.93 | 177.44 |  | 3 |
| 1329 | 25.49 | 1 | 2250 | 50 | 1 | 3 | 1 | 1 | 84.5 | 11.65 | 12.3 | 396.56 | 8.53 | 25.94 | 53.10 |  | 6 |
| 1330 | 26.55 | 2 | 4300 | 50 | 3 | 2 | 2 | 1 | 84.2 | 11.525 | 12.9 |  |  | 15.72 | 41.15 |  | 3 |
| 1331 | 34 | 1 | 2900 | 50 | 3 | 3 | 1 | 1 | 91.2 | 12.065 | 12.9 | 441.34 | 4.36 | 11.17 | 67.68 |  | 5 |
| 1332 | 21.95 | 1 | 3300 | 50 | 3 | 3 | 1 | 2 | 84.7 | 12.695 | 12.8 | 550.69 | 4.64 | 15.41 | 75.17 |  | 5 |
| 1333 | 32.07 | 2 | 3600 | 50 | 2 | 2 | 2 | 1 | 85.4 | 11.27 | 12.2 | 269.19 | 5.70 | 4.79 | 51.04 |  | 7 |
| 1334 | 35.98 | 2 | 3500 | 50 | 2 | 3 | 1 | 2 | 91.2 | 12.78 | 12.6 | 361.91 | 8.15 |  |  |  | 7 |
| 1335 | 32.26 | 2 | 3000 | 50 | 5 | 4 | 1 | 1 | 86.1 | 11.875 | 13.1 | 369.46 | 1.94 | 7.43 | 33.94 |  | 7 |
| 1336 | 20.34 | 2 | 3150 | 50 | 3 | 3 | 1 | 1 | 80.3 | 9.225 | 13.3 |  |  | 15.90 | 91.92 |  | 7 |
| 1337 | 4.67 | 2 | 2850 | 53 | 2 | 4 | 1 | 1 | 66.5 | 7.49 | 11.8 | 157.75 | 4.31 | 3.58 | 55.05 |  | 0 |
| 1338 | 6.14 | 2 | 3450 | 50 | 2 | 2 | 2 | 1 | 66.6 | 7.485 | 11.9 | 155.21 | 2.77 | 5.62 | 47.84 |  | 0 |
| 1339 | 29.86 | 2 | 3300 | 50 | 3 | 4 | 1 | 1 | 93 | 13.03 | 12.2 | 310.79 | 3.27 | 10.11 | 52.17 |  | 4 |
| 1340 | 29.31 | 1 | 3800 | 53 | 4 | 3 | 1 | 1 | 89.3 | 12.97 | 12.4 | 261.07 | 6.95 | 9.50 | 49.08 |  | 5 |
| 1341 | 23 | 2 | 3600 | 50 | 4 | 3 | 2 | 1 | 79.1 | 10.168 | 9.8 | 515.31 | 4.06 | 69.67 | 62.81 |  | 7 |
| 1342 | 5.52 | 1 | 3000 | 50 | 3 | 2 | 2 | 1 | 64.8 | 9.185 | 10.9 |  |  | 25.80 | 55.05 |  | 0 |
| 1343 | 16.72 | 2 | 3150 | 50 | 3 | 3 | 2 | 1 | 75.1 | 9.29 | 12.4 |  |  |  |  |  | 3 |
| 1344 | 31.8 | 2 | 3600 | 51 | 3 | 2 | 1 | 1 | 94.2 | 13.675 | 13.3 | 153.95 | 4.86 | 24.89 | 55.05 |  | 4 |
| 1345 | 20.34 | 1 | 3700 | 50 | 3 | 3 | 1 | 2 | 78.8 | 9.17 | 12 |  |  | 13.93 | 71.27 |  | 6 |
| 1346 | 12.81 | 2 | 3550 | 51 | 2 | 2 | 1 | 2 | 74.3 | 8.13 | 12 |  |  |  |  |  | 5 |
| 1347 | 3.09 | 2 | 3800 | 50 | 3 | 3 | 1 | 2 | 70.1 | 8.775 | 10.3 |  |  |  |  |  | 2 |
| 1348 | 1.58 | 2 | 3100 | 50 | 3 | 4 | 1 | 1 | 54.1 | 5.165 | 9.6 |  |  |  |  |  | 1 |
| 1349 | 9.72 | 1 | 3300 | 50 | 3 | 3 | 1 | 1 | 72 | 9.065 | 11.8 |  |  |  |  |  | 4 |
| 1350 | 7.69 | 2 | 3100 | 50 | 3 | 3 | 2 | 1 | 66.1 | 8.875 | 13.3 |  |  |  |  |  | 1 |
| 1351 | 9.72 | 1 | 3600 | 50 | 3 | 3 | 1 | 1 | 69.6 | 8.335 | 10.8 | 394.96 | 4.24 | 8.70 | 79.42 |  | 1 |
| 1352 | 14.06 | 1 | 3400 | 50 | 2 | 3 | 2 | 1 | 78.1 | 12.125 | 11.4 |  |  |  |  |  | 5 |
| 1353 | 4.04 | 2 | 3050 | 50 | 3 | 2 | 1 | 1 | 59.1 | 6.675 | 11.1 |  |  |  |  |  | 0 |
| 1354 | 22.7 | 1 | 3150 | 49 | 3 | 2 | 2 | 1 | 83.4 | 11.2 | 12.2 | 401.95 | 3.45 | 11.07 | 111.77 |  | 5 |
| 1355 | 21.82 | 2 | 2850 | 49 | 3 | 3 | 1 | 1 | 80.2 | 9.71 | 13 |  |  |  |  |  | 4 |
| 1356 | 22.51 | 1 | 2900 | 50 | 2 | 2 | 2 | 1 | 76.4 | 8.775 | 11.8 | 348.61 | 3.53 | 16.76 | 85.06 |  | 6 |
| 1357 | 25 | 2 | 3350 |  | 2 | 1 | 1 | 1 | 88.4 | 11.42 | 12.1 | 322.14 | 5.84 | 17.78 | 68.73 |  | 3 |
| 1358 | 5.62 | 1 | 3950 | 50 | 2 | 2 | 1 | 1 | 67 | 7.02 | 10.1 | 217.37 | 4.08 |  |  |  | 1 |
| 1359 | 19.06 | 2 | 3400 | 57 | 3 | 2 | 1 | 1 | 76.5 | 8.57 | 11.3 | 653.87 | 3.94 | 4.60 | 90.82 |  | 6 |
| 1360 | 22.9 | 1 | 3600 | 49 | 3 | 3 | 1 | 2 | 86.1 | 13.25 | 15.3 |  |  | 12.74 | 55.30 |  | 3 |
| 1361 | 34.79 | 1 | 3400 | 50 | 3 | 3 | 1 | 1 | 95.9 | 15 | 14.2 | 220.66 | 4.94 | 18.87 | 70.16 |  | 7 |
| 1362 | 19.98 | 2 | 2515 | 47 | 3 | 4 | 2 | 1 | 80.1 | 9.94 | 11.7 | 787.46 | 9.12 | 21.64 | 76.83 |  | 3 |
| 1363 | 10.38 | 2 | 3400 | 49 | 2 | 3 | 1 | 1 | 69.6 | 8.2 | 11 |  |  |  |  |  | 6 |
| 1364 | 9.72 | 1 | 3950 | 55 | 3 | 3 | 1 | 1 | 75.1 | 9.695 | 12 |  |  |  |  |  | 3 |
| 1365 | 10.91 | 1 | 3300 | 50 | 2 | 3 | 1 | 1 | 74.2 | 9.88 | 12.4 |  |  |  |  |  | 1 |
| 1366 | 33.94 | 1 | 3750 | 50 | 3 | 3 | 1 | 1 | 95.1 | 14.685 | 11 | 238.68 | 4.76 | 14.20 | 70.16 |  | 2 |
| 1367 | 28.65 | 2 | 2700 |  | 2 | 2 | 2 | 1 | 86.1 | 11.56 | 13.1 | 250.32 | 4.72 | 5.78 | 49.51 |  | 4 |
| 1368 | 31.21 | 1 | 3400 | 49 | 2 | 1 | 1 | 1 | 89.1 | 11.715 | 12 | 242.92 | 7.83 | 8.08 | 63.74 |  | 7 |
| 1369 | 24.64 | 1 | 4100 | 54 | 3 | 3 | 1 | 1 | 84.9 | 12.58 | 11 |  |  | 10.83 | 64.08 |  | 5 |
| 1370 | 32.99 | 2 | 3350 | 51 | 2 | 3 | 2 | 1 | 93 | 13.93 | 13.6 |  |  |  |  |  | 5 |
